# Headache disorders and relevant sex and socioeconomic patterns in adolescents and young adults across 204 countries and territories: an updated global analysis

**DOI:** 10.1186/s10194-023-01648-4

**Published:** 2023-08-18

**Authors:** Rongguang Ge, Jie Chang, Yongjun Cao

**Affiliations:** 1 Medical College of Soochow University, Soochow University, Suzhou, China; 2 Department of Neurology, Second Affiliated Hospital of Soochow University, 1055 Sanxiang Road, 215123 Suzhou, China; 3 Department of Occupational and Environmental Health, School of Public Health, Medical College of Soochow University, Soochow University, Suzhou, China; 4 Jiangsu Key Laboratory of Preventive and Translational Medicine for Geriatric Disease, Soochow University, Suzhou, China

## Abstract

**Background:**

Primary headache disorders are a group of highly prevalent and disabling neurological diseases that mainly consist of migraine and tension-type headache (TTH). A previous study showed that the burden of headaches peaked at a working age that ranged from 15 to 49, particularly among females, affecting their productivity and severely damaging their social interactions.

**Methods:**

The latest dataset was retrieved from the Global Burden of Disease (GBD) Study 2019. Three indicators, including prevalence, incidence, and years lived with disability (YLDs), were adopted for evaluation. The overall and specific headache burdens were fully compared and analysed at global, regional, and national levels. The ratio of female YLD rates to male YLD rates due to headaches was calculated to estimate the sex pattern. Finally, we utilized the two-tailed Spearman test to explore the potential association between socioeconomic background and headaches among young people.

**Results:**

Globally, for overall headache disorders, a total of 2,049,979,883 prevalent cases (95% uncertainty interval (UI): 1,864,148,110 to 2,239,388,034), 601,229,802 incident cases (95% UI: 530,329,914 to 681,007,934), and 38,355,993 YLDs (95% UI: 7,259,286 to 83,634,503) were observed for those aged 10 to 54 in 2019. Sex differences were widely found for all headache types among adolescents and young adults, especially migraine. However, the most interesting finding was that the associations we tested between the socioeconomic environment and young headache patients were positive, regardless of region or specific country or territory.

**Conclusions:**

Overall, the global burden of headaches in adolescents and young adults largely increased from 1990 to 2019. Although slight declines were observed in sex differences, they remained significant and challenging. The positive correlations between headache and socioeconomic background among young people were relatively inconsistent with previous investigations, and several related hypotheses were proposed for explanation. Interdisciplinary actions involving education, policy- and law-making, and basic medical practice are desperately needed to further fight against the headache burden, promote gender equality in headache care, and eliminate the stigmatization of headache patients in student and working groups.

**Supplementary Information:**

The online version contains supplementary material available at 10.1186/s10194-023-01648-4.

## Introduction

Headache disorders, as highly prevalent non-communicable diseases in clinical practice, were reported to influence approximately three billion people worldwide in 2016 [1], among whom 1.04 billion cases could be attributed to migraine and 1.89 billion to tension-type headache (TTH). In 2019, the absolute number of patients suffering from migraine increased by nearly 8% to 1.13 billion [2], making more individuals face the direct threat of headache disorders and society shoulder heavier burdens associated with finance and the healthcare system.

Characterized by both chronic and recurrent onset, primary headache disorders were classified into four subtypes according to the International Classification of Headache Disorders, 3^rd^ edition (ICHD-3) published in 2018, including migraine, TTH, trigeminal autonomic cephalalgia, and other primary headaches [3]. Migraine is considered a long-term condition that features headache of moderate or severe intensity and a combination of typical characteristics, which mainly consists of aggravation by routine physical activity and associations with nausea, vomiting, photophobia, and phonophobia. On the other hand, TTH is defined as a disorder with clinical manifestations including hatband-like distributed headache but with relatively less pronounced associated features.

Notably, through previous investigations, significant age-specific and sex-related discrepancies have been widely observed in headache patients. Based on the findings from the Global Burden of Disease (GBD) Study 2016 [1], researchers suggested that headache disorders have become extremely frequent and disabling in females, particularly those aged between 15 and 49, resulting in a total of 20.3 million years lived with disability (YLDs) due to migraine and 2.9 million due to TTH. In fact, adolescents and young adults, as the main studying and working age group in the general population, are more likely to be jeopardized by the global prevalence of headache disorders, which may affect their productivity and create a constant need for healthcare service during the onset, placing burdens on caregivers and posting extra economic challenges to their daily lives [4–7]. However, although increasingly innovative and technology-oriented pharmacological and non-pharmacological therapies have been proposed, stigmatization remains the major barrier to better fighting the aggression of headache disorders worldwide. Usually, presented as stereotypes against headache patients in the mass media [8] and the ongoing “gendering” of the disease [9], stigmatization might originate from the ignorance of this disorder and stop patients from receiving timely and proper treatment, forcing them to tolerate the suffering or even leading to the aggravation of their symptoms.

Therefore, a full analysis and description of the overall disease status and its changing trend in age groups that are severely threatened by headache disorders are necessary. Fortunately, a previous study [10] based on the GBD Study performed a detailed investigation into the burden of headaches in those aged between 5 and 19 from 2007 to 2017, providing well-organized and systematic data for further evaluation. However, sadly, these findings may be outdated and insufficient in the current circumstances. As a result, in our present work, by utilizing the latest dataset retrieved from the GBD Study 2019, our research team analysed the overall headache burden and two major primary headache types, migraine and TTH, in the young population aged 10 to 54 at global, regional, and national levels. Furthermore, we also explored the sexual and socioeconomic patterns of headache disorder by age group and geographic location. Through this study, our research team aimed to comprehensively demonstrate the current status of headache disorder in young people and particularly reveal the potential gender and socioeconomic features by specific country and territory worldwide. Additionally, we sincerely hope our discoveries can be helpful to erase disease- and gender-related stigmatization in the future and contribute to better healthcare equality worldwide, providing necessary information and data for medical practitioners, policy-makers, and interdisciplinary researchers in the real world.

## Methods and materials

### Overview

Operated by the Institute for Health Metrics and Evaluation (IHME) and the University of Washington, the GBD Study (https://ghdx.healthdata.org/gbd-2019) provides the most systematic, comprehensive, and highly available assessment of published and contributed data on incidence, prevalence, and mortality for a mutually exclusive and collectively exhaustive list of illnesses and injuries [11, 12]. In GBD Study 2019, a total of 86,249 disease or injury-related data sources worldwide, including 31,499 sources reporting incidence, 19,773 reporting prevalence, 19,354 reporting mortality, and 26,631 reporting other metrics, were analysed by the research team [12]. Moreover, the study followed the Guidelines for Accurate and Transparent Health Estimates Reporting (GATHER).

In our study, all data used for analysis on headache disorders in adolescents and young adults, including migraine and TTH, were identified and retrieved by the GBD research team from related reviews, which were published up to the end of September 2017. The search strings were as follows: (((((("migraine disorders"[MeSH Terms] OR migraine[All Fields]) AND ((prevalence[Title/Abstract] OR incidence[Title/Abstract] OR remission[Title/Abstract] OR epidemiology[Title/Abstract]))))))) and ((((("headache"[MeSH Terms]) OR ("headache"[Title/Abstract] AND "tension"[Title/Abstract])) AND ("epidemiology"[Title/Abstract] OR "prevalence"[Title/Abstract] OR "incidence"[Title/Abstract] OR "remission"[Title/Abstract])))). After searching and screening, a total of 153 data sources were collected for primary headache disorder modelling. Only publications with great representativeness or convincing data points based on a large research population were considered, while medical claims data were excluded due to their lack of robustness and reliability. A more detailed and comprehensive description of the data input, citation, and disease modelling can be found at https://ghdx.healthdata.org/gbd-2019/data-input-sources.

### Disease definition and diagnosis

In terms of the definition of headache disorders, in the GBD Study 2019, the research team described migraine as a disabling primary neurological condition typically characterized by recurrent moderate or severe unilateral pulsatile headaches. However, migraines with or without aura were not distinguished by the researchers and only reported the overall migraine status and burden. TTH was labelled as a dull, non-pulsatile, diffuse, band-like (or vice-like) pain in the head or neck that was of mild to moderate intensity. The disease codes representing migraine and TTH were G43-G43.919 and G44.2-G44.229, G44.4-G44.41 in the International Classification of Diseases, 10^th^ revision (ICD-10), and 346–346.93 and 307.81, 339.1–339.12, 339.3 in the International Classification of Diseases, 9^th^ revision (ICD-9).

The diagnostic criteria strictly followed the ICHD-3, which states that a probable diagnosis of headache disorder must meet at least 4 of the 5 listed criteria, while a definite diagnosis must meet all criteria, as shown below [3]:

For migraine, the diagnostic criteria included 1. at least five attacks fulfilling criteria 2–5; 2. headache attacks lasting 4–72 h (untreated or unsuccessfully treated); 3. headache with at least two of the following four characteristics: a. unilateral location, b. pulsating quality, c. moderate or severe pain intensity, d. aggravation by or causing avoidance of routine physical activity; 4. at least one of the following during headache: a. nausea and/or vomiting, b. photophobia and phonophobia; and 5. not better accounted for by another ICHD-3 diagnosis.

For TTH, the diagnostic criteria included 1. at least 10 attacks fulfilling criteria 2–5; 2. lasting from 30 min to 7 days; 3. at least two of the following four characteristics: a. bilateral location, b. pressing or tightening (non-pulsating) quality, c. mild or moderate intensity, d. not aggravated by routine physical activity such as walking or climbing stairs; 4. both of the following: a. no nausea or vomiting, b. no more than one of photophobia or phonophobia; and 5. not better accounted for by another ICHD-3 diagnosis.

### Geographic classification

In the GBD Study 2019, a total of 204 countries and territories worldwide were included in the database. Subsequently, a total of 21 GBD regions were generated, consisting of Andean Latin America, Australasia, Caribbean, Central Asia, Central Europe, Central Latin America, Central Sub-Saharan Africa, East Asia, Eastern Europe, Eastern Sub-Saharan Africa, High-income Asia Pacific, High-income North America, North Africa and Middle East, Oceania, South Asia, Southeast Asia, Southern Latin America, Southern Sub-Saharan Africa, Tropical Latin America, Western Europe, and Western Sub-Saharan Africa.

### Sociodemographic index (SDI)

The sociodemographic index (SDI) is a joint assessment of the local socioeconomic environment by combining information on lagged distributions of per capita income, the average educational attainment among individuals aged 15 years and older, and the total fertility rate among individuals younger than 25 years [13]. In the GBD Study 2019, each geographic location has its corresponding SDI value, based on which the study team separated 204 countries and territories into 5 groups, including low (< 0.46), low-middle (0.46 ~ 0.60), middle (0.61 ~ 0.69), high-middle (0.70 ~ 0.81), and high (> 0.81) SDI [14].

### Indicators of disease status and burden

In our current paper, three main indicators were adopted to comprehensively evaluate the disease status and burden in adolescents and young adults across the world. The prevalence is described as the actual existing cases attributable to a specific disease or disability in the general population; when measured by rate, it represents the current patients per 100,000 population. The incidence refers to the newly diagnosed cases in a certain time and geographic background; when measured by rate, it can be considered the recently diagnosed patients per 100,000 population. Finally, the YLD is the main metric for assessing the life lost due to any short-term or long-term morbidity when measured by rate, which means the total healthy years lost to disease per 100,000 population.

### Data processing and disease modelling

For nonfatal disease, the disease model Bayesian meta-regression (DisMod-MR 2.1) modelling tool, which is software designed to generate a Bayesian geospatial disease model, was adopted to calculate headache incidence and prevalence [15]. By collecting all available high-quality epidemiological data, the DisMod-MR 2.1 modelling tool successfully performed the estimation of nonfatal disease burdens of migraine and TTH.

### Statistical analysis and results

Statistical analyses were performed as follows. First, we evaluated the overall headache burden at the global level and then furthered the analysis by region and specific country and territory. Next, the sex and age patterns of headache disorders in adolescents and young adults were observed by using detailed age groups and ratios of the YLDs for females to those for males. Finally, the data on SDI by region were extracted from the GBD Study, and a Spearman correlation analysis was conducted to fully assess their potential associations with headache burden in the young population.

The presentation of our findings was mainly based on visualization tools, including GraphPad Prism software (Boston, Massachusetts), ArcGIS software (Redlands, California), and Adobe Illustrator software (San Jose, California). GraphPad Prism software was used for the creation of line charts and bar graphs, while ArcGIS software was used to generate coloured world maps. Finally, all figure parts were combined with Adobe Illustrator software to obtain a full-size artwork.

## Results

### Global level

As shown in Figure S1 and Table 1, globally, regarding overall headache disorders in adolescents and young adults, a total of 2,049,979,883 prevalent cases (95% uncertainty interval (UI): 1,864,148,110 to 2,239,388,034), 601,229,802 incident cases (95% UI: 530,329,914 to 681,007,934), and 38,355,993 YLDs (95% UI: 7,259,286 to 83,634,503) were observed in 2019. Moreover, slight increases were found in the prevalence and YLD and the opposite in incidence. Per 100,000 population, the prevalence increased by 1.39% to 40,884.21 cases (95% UI: 37,178.03 to 44,661.71), the YLD increased by 3.94% to 764.96 years (95% UI: 144.78 to 1,667.98), and the incidence decreased by 0.71% to 11,990.75 cases (95% UI: 10,576.75 to 13,581.83) from 1990 to 2019.

**Table 1:** The global absolute numbers, rates, and change percents of rate of overall headache disorders, migraine, and TTH in adolescents and young adults

	**Prevalence (95% UI)**			**Incidence (95% UI)**			**YLD (95% UI)**		
	**Absolute number (2019)**	**Rate per 100,000 population (2019)**	**Change percent of rate (1990–2019)**	**Absolute number (2019)**	**Rate per 100,000 population (2019)**	**Change percent of rate (1990–2019)**	**Absolute number (2019)**	**Rate per 100,000 population (2019)**	**Change percent of rate (1990–2019)**
**Headache disorders**	2,049,979,883, (1,864,148,110, 2,239,388,034)	40,884.21, (37,178.03, 44,661.71)	1.39%	601,229,802, (530,329,914, 681,007,934)	11,990.75, (10,576.75, 13,581.83)	-0.71%	38,355,993, (7,259,286, 83,634,503)	764.96, (144.78, 1,667.98)	3.94%
**Migraine**	938,932,847, (809,364,423, 1,091,101,799)	18,725.81, (16,141.73, 21,760.62)	3.73%	72,479,349, (62,228,889, 82,376,780)	1,445.51, (1,241.08, 1,642.90)	-2.55%	34,934,884, (4,726,361, 80,644,048)	696.73, (94.26, 1,608.34)	4.01%
**TTH**	1,552,912,194, (1,331,676,025, 1,775,658,298)	30,970.83, (26,558.56, 35,413.22)	0.25%	528,750,453, (459,812,890, 608,377,209)	10,545.25, (9,170.38, 12,133.30)	-0.45%	3,421,109, (986,631, 12,058,300)	68.23, (19.68, 240.49)	3.32%

In terms of specific subtypes, the findings varied. For migraine in adolescents and young adults, a total of 938,932,847 prevalent cases (95% UI: 809,364,423 to 1,091,101,799), 72,479,349 incident cases (95% UI: 62,228,889 to 82,376,780), and 34,934,884 YLDs (95% UI: 4,726,361 to 80,644,048) were observed worldwide in 2019. Similarly, per 100,000 population, the prevalence increased by 3.73% to 18,725.81 cases (95% UI: 16,141.73 to 21,760.62), the YLD increased by 4.01% to 696.73 years (95% UI: 94.26 to 1,608.34), and the incidence decreased by 2.55% to 1,445.51 cases (95% UI: 1,241.08 to 1,642.90) from 1990 to 2019. For TTH in adolescents and young adults, a total of 1,552,912,194 prevalent cases (95% UI: 1,331,676,025 to 1,775,658,298), 528,750,453 incident cases (95% UI: 459,812,890 to 608,377,209), and 3,421,109 YLDs (95% UI: 986,631 to 12,058,300) were observed worldwide in 2019. Per 100,000 population, the prevalence increased by 0.25% to 30,970.83 cases (95% UI: 26,558.56 to 35,413.22), the YLD increased by 3.32% to 68.23 years (95% UI: 19.68 to 240.49), and the incidence decreased by 0.45% to 10,545.25 cases (95% UI: 9,170.38 to 12,133.30) from 1990 to 2019.

### Regional level

When analysing the overall headache disorders in adolescents and young adults by region, the prevalence rate ranged from 30,824.21 to 52,516.87 cases per 100,000 population, with the highest in Western Europe and the lowest in Eastern Sub-Saharan Africa. The incidence rate ranged from 8865.99 to 15,765.23 cases per 100,000 population, with the highest in high-income North America and the lowest in East Asia. Finally, the YLD rate ranged from 472.47 to 1,035.61 years per 100,000 population, with the highest in Western Europe and the lowest in Eastern Sub-Saharan Africa. More detailed data are available in Table S1.

Regarding the specific headache types in adolescents and young adults by region, for migraine, the prevalence rate ranged from 11,151.59 to 25,256.84 cases per 100,000 population, with the highest in Western Europe and the lowest in Eastern Sub-Saharan Africa. The incidence rate ranged from 1030.6 to 1672.82 cases per 100,000 population, with the highest in high-income North America and the lowest in Eastern Sub-Saharan Africa. The YLD rate ranged from 419.07 to 939.14 years per 100,000 population, with the highest in Western Europe and the lowest in Eastern Sub-Saharan Africa. For TTH, the prevalence rate ranged from 22,251.21 to 43,035.48 cases per 100,000 population, with the highest in high-income North America and the lowest in East Asia. The incidence rate ranged from 7681.95 to 14,092.41 cases per 100,000 population, with the highest in high-income North America and the lowest in East Asia. The YLD rate ranged from 53.4 to 126.01 years per 100,000 population, with the highest in Eastern Europe and the lowest in Eastern Sub-Saharan Africa. More detailed data are available in Tables S2 and S3.

### National level

**Fig. 1:**
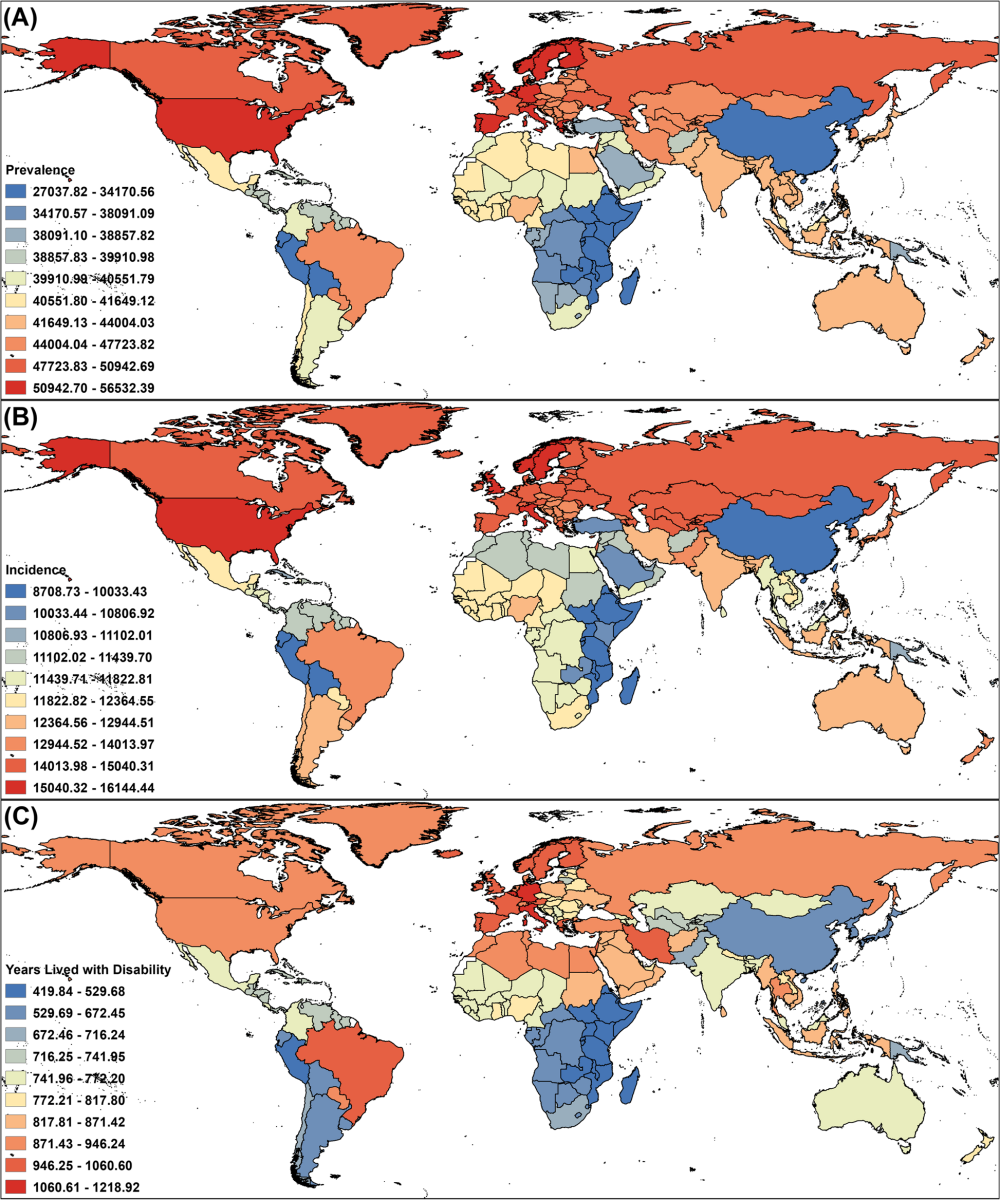
Rates of prevalence (**A**), incidence (**B**), and YLD (**C**) of overall headache disorders per 100,000 population in adolescents and young adults by country and territory in 2019

As shown in Fig. 1A-C and Table S4, in terms of the national burden of overall headache disorders in adolescents and young adults, the prevalence rate ranged from 27,037.82 to 56,532.39 cases per 100,000 population, with the highest in Italy and the lowest in Ethiopia. The incidence rate ranged from 8,708.73 to 16,144.44 cases per 100,000 population, with the highest in Norway and the lowest in North Korea. The YLD rate ranged from 419.84 to 1,218.92 years per 100,000 population, with the highest in Belgium and the lowest in Ethiopia.

Regarding specific headache types in adolescents and young adults by nation, for migraine, the prevalence rate ranged from 10,148.33 to 30,611.94 cases per 100,000 population, with the highest in Belgium and the lowest in Ethiopia. The incidence rate ranged from 945.07 to 1,787.19 cases per 100,000 population, with the highest in Norway and the lowest in Singapore. The YLD rate ranged from 375.58 to 1,126.25 years per 100,000 population, with the highest in Belgium and the lowest in Ethiopia. For TTH, the prevalence rate ranged from 20,196.26 to 45,354.64 cases per 100,000 population, with the highest in Norway and the lowest in Ethiopia. The incidence rate ranged from 7,518.47 to 14,357.25 cases per 100,000 population, with the highest in Norway and the lowest in North Korea. Finally, the YLD rate ranged from 44.25 to 129.18 years per 100,000 population, with the highest in the Russian Federation and the lowest in Ethiopia. More detailed data are available in Figures S2 and S3 and Tables S5 and S6.

### Sex and age patterns

**Fig. 2:**
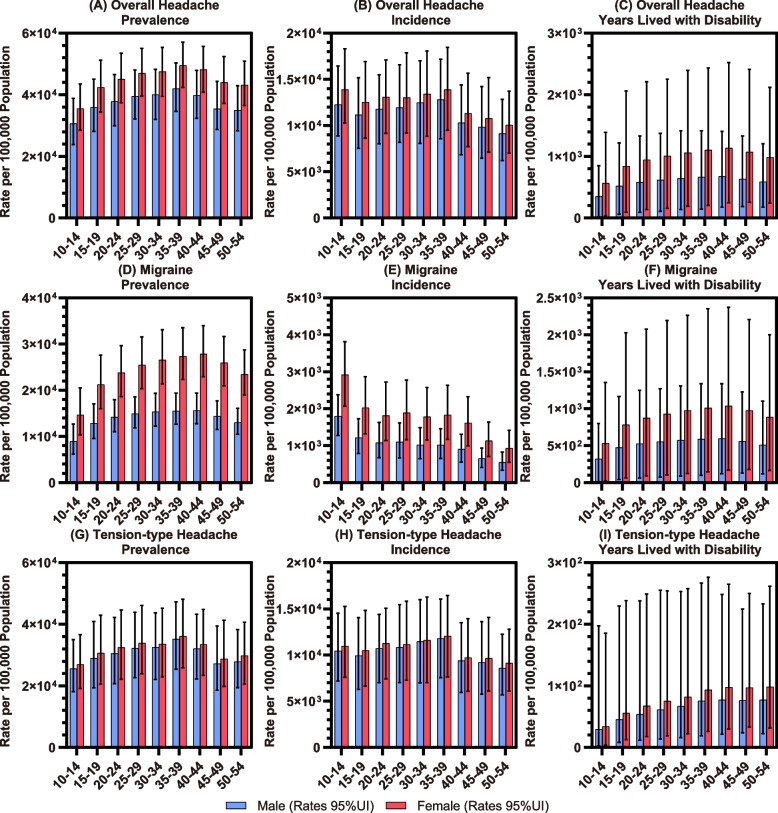
Rates of prevalence, incidence, and YLD of overall headache disorders (**A**-**C**), migraine (**D**-**F**), and TTH (**G**-**I**) per 100,000 population in adolescents and young adults by sex and age group in 2019

As shown in Fig. 2 and Tables S7-S9, when analysed by age group, the overall prevalence rate of headache disorders in adolescents and young adults reached its peak between the ages of 35 and 44, which is largely consistent with those of migraine and TTH. In terms of the incidence rate, slight increases were widely observed in the population aged from 20 to 39 and then turned into rapid declines with ageing. The YLD rate peaked between the ages of 40 and 44 for overall headache disorders, migraine, and TTH.

**Fig. 3:**
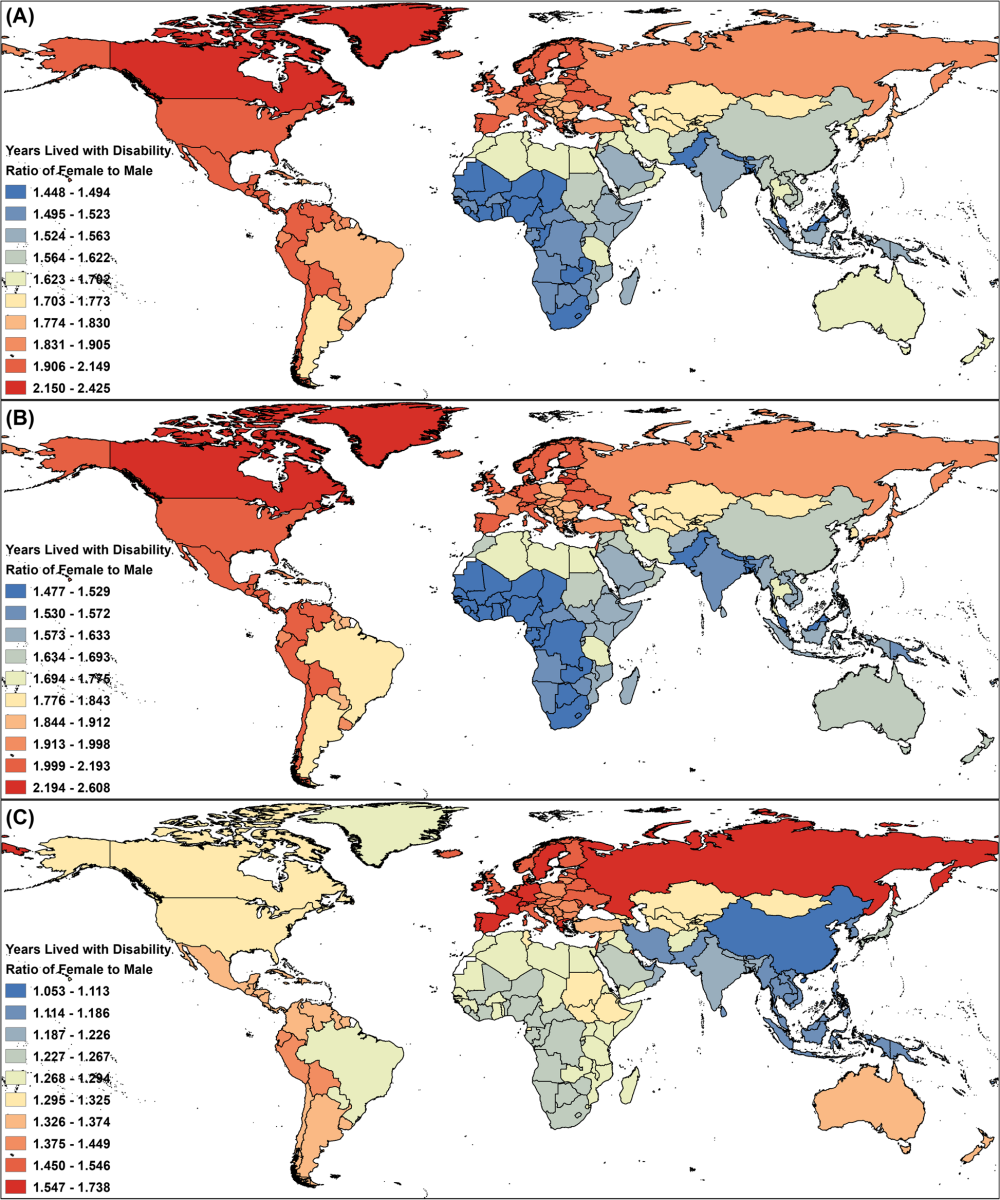
Ratios of female YLD rates to male YLD rates of overall headache disorders (**A**), migraine (**B**), and TTH (**C**) in adolescents and young adults by country and territory in 2019

Regarding sex differences, as presented in Figure S4 and Table S10, from 1990 to 2019, the ratio of females to males calculated by their YLD rates showed constant declines, regardless of the specific headache type. For overall headache disorders, the ratio of YLDs in females and YLDs in males decreased from approximately 1.7 in 1990 to 1.655 in 2019. For migraine, the ratio dropped from 1.751 in 1990 to 1.702 in 2019. For TTH, the ratio declined from 1.267 in 1990 to 1.247 in 2019. When further analysed by country and territory, according to Fig. 3 and Table S11, although the findings showed significant diversities, the ratios of female YLD rates to male YLD rates were all greater than 1. For overall headache disorders, the ratio of specific geographic locations worldwide in 2019 ranged from 1.448 to 2.425, with the highest in Canada and the lowest in Malaysia. For migraine, the ratio ranged from 1.477 to 2.608, with the highest ratio in Canada and the lowest in Malaysia. For TTH, the ratio relatively decreased compared with those of migraine and headache disorders and ranged from 1.053 to 1.738, with the highest in Spain and the lowest in China.

### Correlation with socioeconomic background

**Fig. 4:**
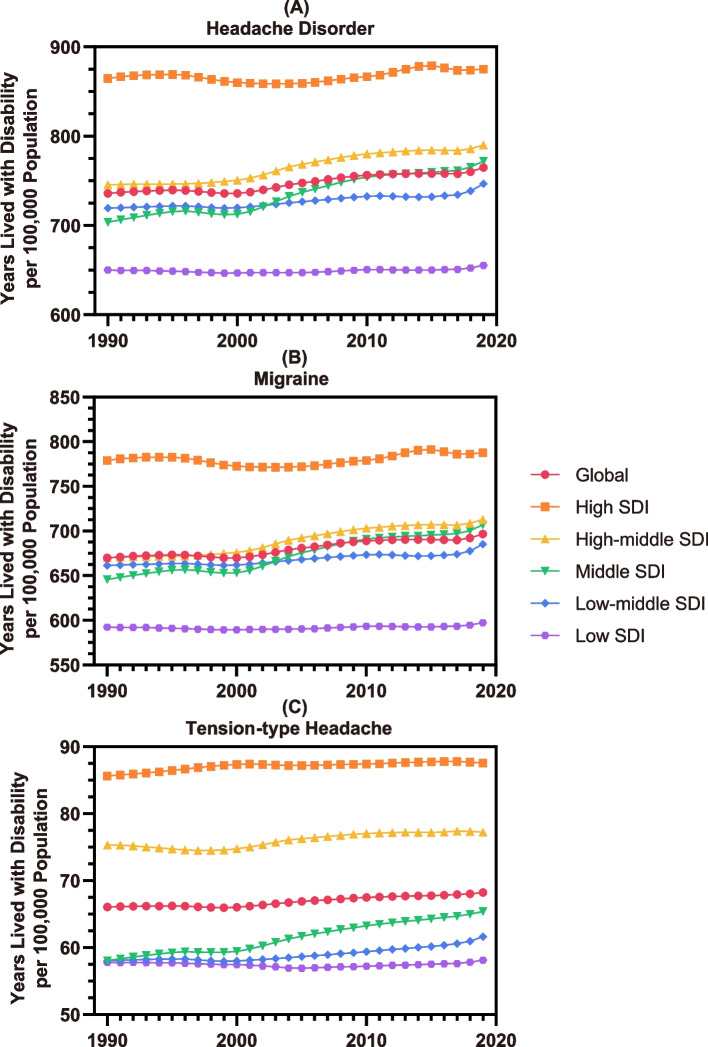
Rates of YLDs per 100,000 population of overall headache disorders (**A**), migraine (**B**), and TTH (**C**) in adolescents and young adults across five major SDI regions from 1990 to 2019

As shown in Fig. 4 and Tables S12-S14, rough analyses of the YLD rates in different regions with quintile-distributed SDIs suggested a positive correlation between the socioeconomic factor and headache burden in young people, with regions with higher SDIs generally showing greater YLD rates per 100,000 population, especially those categorized as having a high SDI.

**Fig. 5:**
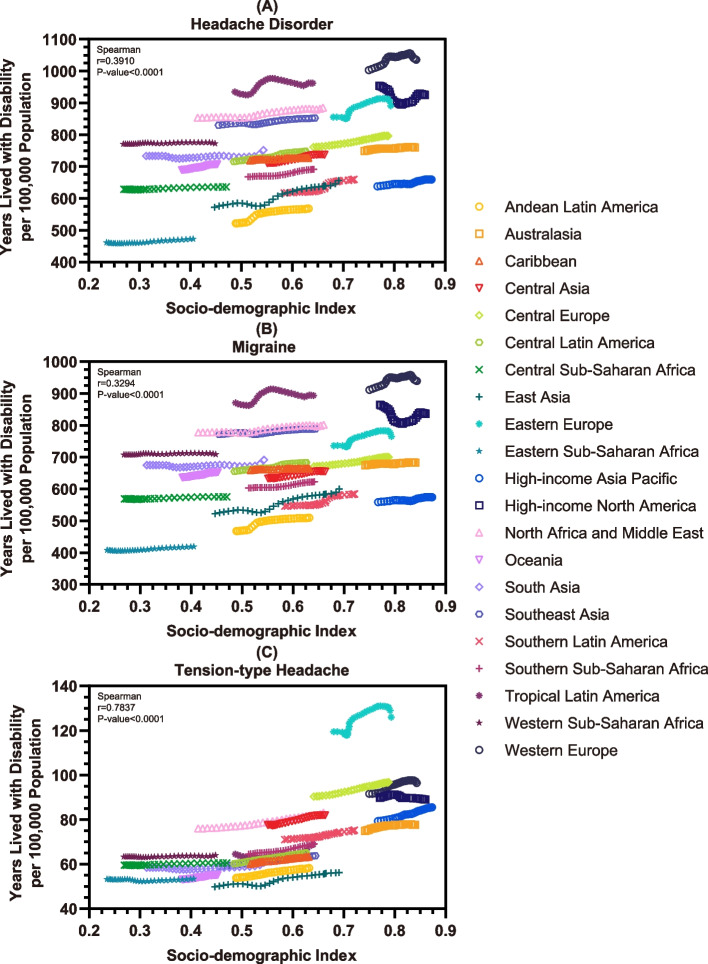
The associations between socio-demographic factor and overall headache disorders (**A**), migraine (**B**), and TTH (**C**) in adolescents and young adults and the results of Spearman test by region

When analysed more statistically by geographic region and nation. According to Fig. 5 and Tables S15-S17, through two-tailed Spearman tests, the disease burdens of headaches, including overall headache disorders, migraine, and TTH, at the regional level were proven to be positively associated (*r* = 0.3910, *r* = 0.3294, and *r* = 0.7837, respectively) with local socioeconomic environments, which were all statistically significant (*P* value < 0.0001). Moreover, as shown in Figure S5 and Table S18, the findings at the national level were also highly consistent, and the Spearman analysis suggested *r* = 0.4975 for overall headache disorders, *r* = 0.4223 for migraine, and *r* = 0.7150 for TTH. Similarly, all results showed statistical significance (*P* value < 0.0001).

## Discussion

In our current paper, by fully using the latest dataset collected from the GBD Study 2019, our research team has performed an updated analysis and description of headache disorders in adolescents and young adults worldwide, as well as relevant sex and socioeconomic patterns worldwide, providing better reinforcement and supplementary findings for previous publication and foundation for future research. In fact, a recent publication [16] shared a similar research topic with our current paper that targeted young headache patients and proposed informative conclusions on the rising trend of disease burden over the past decades. In comparison, our present work reported the overall and specific headache status in a larger age group, which was basically throughout the entire studying and working period. In addition, our research team focused on and explored more deeply the aspects of headache-associated gender and socioeconomic patterns in the young population at a detailed national level and acquired different findings from previous studies. Generally, our results suggested that from 1990 to 2019, primary headache disorders, including migraine and TTH, were significantly prevalent and disabling in the young population, regardless of global, regional, or national levels. Regarding the sex pattern, the female population undertook over 60% of the total healthy life years lost to disease-related disability, showing that they were more likely to become victims of headache disorders, although this sex difference observed in TTH was relatively less pronounced compared with those in overall headache disorders and migraine. The most interesting conclusion of our current work was the positive correlation found between headache burden and local socioeconomic background in adolescents and young adults, which conflicted with previous findings, and plausible reasons and hypotheses have been proposed.

Regarding the sex pattern widely reported in headache patients, according to several former investigations [17–19], sex hormones were believed to play important roles in the prevalence, frequency, and intensity of primary headache disorders, among which oestrogen was especially considered a significant trigger for onset. By evaluating the natural course of headache throughout the lifespan of female patients, hormonal changes, which were usually observed from puberty to pregnancy to menopause/post-menopause, were closely involved in the development of the disease. In addition, animal-based experiments [20, 21] also discovered the potential association between calcitonin gene-related peptide (CGRP) and hormones, the excitability and sensitization of the former of which could be regulated by the oestrous cycle in rat models [22]. While CGRP was found to be released during headache attacks and recognized as a direct inductor for this disorder [23]. Therefore, the sex-related discrepancy in headaches in adolescents and young adults in our current paper was understandable and in line with the conclusions from previous studies.

In terms of the socioeconomic features of headaches in the young population, positive correlations were proven between local socioeconomic background and headache burden, and those who lived in regions with greater social and economic development were more likely to face the threat and challenge posed by headache disorders. According to our results, Western Europe and high-income North America were identified as the leading two geographic regions that were severely impacted by the disease. Countries and territories located in these two regions suffered from far graver headache-induced disability and healthy life loss, particularly Germany, Belgium, and Italy. These findings were surprisingly inconsistent with previous investigations [24–27], and our research team provided three hypotheses listed as follows. First, the most plausible reason for this phenomenon is that the headache prevalence, incidence, and relevant impact were rarely studied and reported in low- and low-middle-SDI regions. In other words, the vast majority of our currently available publications and data concerned with headache disorders were conducted in developed and high, high-middle, or middle SDI regions. This potential explanation was supported by several bibliometric analyses [28–30]. Second, the lack of disease awareness cannot be ignored. Compared with those living in a better socioeconomic environment, patients under a poor socioeconomic background were less likely to receive proper education targeting primary headache disorders, leaving them simply ignorant or confused about this prevalent and disabling disease [31]. These behaviours made patients unable to visit a hospital or medical practitioner in a timely manner, neither being correctly diagnosed nor recorded. Finally, the shortage of medical resources in low- and low-middle-SDI regions also played a role. With a relatively under-invested national healthcare system and the absence of sound policy, patients living in these regions faced greater barriers to reaching high-quality medical services involving neurological specialists [31, 32]. Additionally, the unaffordability and inaccessibility of neuroimaging in resource-limited regions could not be resolved dramatically, making more effort and time poured into the training of primary care providers to identify headache disorders without medical imaging.

When both sex differences and socioeconomic background were combined for analysis, similar outcomes were observed: Western Europe and high-income North America showed greater ratios of female YLD rates to male YLD rates attributable to headache disorders in adolescents and young adults. The reasons and hypotheses for this finding were largely shared with those for the analysis by socioeconomic background solely, that scientific publications of sex patterns of headache in low and low-middle SDI regions were fewer. Moreover, local female patients, or even female medical practitioners, were comparatively less valued due to the economic constraint and specific cultural environment [33], leaving their heavy headache burdens not fully discovered and revealed.

Overall, in our current study, we fully analysed and described the general headache burden in adolescents and young adults at global, regional, and national levels and demonstrated the widely observed sex and socioeconomic differences in the young headache population in detail. However, patients aged from 10 to 54, as the age group with the best productivity and most frequent social activity, are increasingly becoming the main victims of harmful misconception and stigmatization against chronic headaches [34]. Forced by so-called “presenteeism”, working-age headache patients had to continue working while sick, which significantly reduced their efficiency and in turn constantly worsened their well-being, eventually leading to a vicious circle [35, 36]. Besides, it was also reported that unfriendliness and antagonism from colleagues were experienced by patients with chronic pain in the workplace [37], making them feel shameful and guilty about their disease. Another major concern was that the high prevalence and frequency of headache in females gradually came to be an origin of the ongoing “gendering” of the disease. For example, a study based on migraine advertising in the United States of America suggested that some pharmaceutical marketing practices tried to label migraine headaches “women’s disorder” [38], which as a result aggravated the gender bias and brought difficulties for female patients seeking help. Therefore, countermeasures for fighting against the growing gender inequality and disease stigmatization of headache disorders in adolescents and young adults are desperately needed. Sufficient recognition of the disease itself and basic respect for the patients, as well as policy and legal aspects [39], are all supposed to be further emphasized to ensure the welfare of the young headache population. Interdisciplinary studies should also be strongly encouraged. We sincerely hope our findings can be helpful in providing comprehensive and detailed data for the construction and operation of the measures mentioned above, which are dedicated to promoting gender equality and eliminating the stigmatization of headache disorders in adolescents and young adults in the future.

Our current study suffered from shared limitations with other previous GBD studies. First, and most importantly, this paper was fully based on the estimation acquired from related statistical calculations and disease modelling. Therefore, our findings may not necessarily be completely in line with real-world data and should be handled and assessed with caution. Second, the ICHD-3 was adopted in our current study for the definition and diagnosis of overall headache disorders and two subtypes, yet the definition and diagnostic criteria might be constantly updated and modified with a deeper understanding of the disease, leaving a major source of potential bias for this paper. Third, although we have included overall headache disorders, migraine, and TTH, some other relatively rare headache types, such as cluster headache, were not considered, which calls for further investigations on the horizon.

## Conclusion

Generally, the overall headache burden in adolescents and young adults worldwide has increased over the past three decades. Although the sex difference observed in the young headache population has slightly declined, it remains pronounced and challenging. For socioeconomic factors, positive correlations were widely found with the headache burden in young people, regardless of region or specific nation. Therefore, interdisciplinary actions that involve education, policy- and law-making, and medical practice are urgently needed to combat the growing threat of headaches, contribute to better gender equality and eliminate disease stigmatization.

### Supplementary Information

**Additional file 1.**

**Additional file 2.**

## Data Availability

The data used in analysis are presented within the article and supplementary files.

## Abbreviation

TTH: Tension-type headache
GBD: Global Burden of Disease
UI: Uncertainty interval
YLDs: Years lived with disability
ICHD-3: International Classification of Headache Disorders 3^rd^ edition
SDI: Socio-demographic index
IHME: Institute for Health Metrics and Evaluation
ICD-10: International Classification of Diseases 10^th^ revision
ICD-9: International Classification of Diseases 9^th^ revision
GATHER: Guidelines for Accurate and Transparent Health Estimates Reporting
DisMod-MR: Disease-model-Bayesian meta-regression
CGRP: Calcitonin gene-related peptide
