# Supplementary material for: Headache disorders and relevant sex and socioeconomic patterns in adolescents and young adults across 204 countries and territories: an updated global analysis
Source: J Headache Pain. 2023 Aug 18;24(1):110. doi: 10.1186/s10194-023-01648-4 (PMC10436621; doi:10.1186/s10194-023-01648-4)
Supplement: Supplementary file 1 — Additional file 1. [file 10194_2023_1648_MOESM1_ESM.pdf]

Supplementary Figure

- ✧ **Supplementary Figure 1.** Global changing trends of prevalence (A), incidence (B), and YLD (C) of overall headache disorders, migraine, and TTH in adolescents and young adults from 1990 to 2019
- ✧ **Supplementary Figure 2.** Rates of prevalence (A), incidence (B), and YLD (C) of migraine per 100,000 population in adolescents and young adults by country and territory in 2019
- ✧ **Supplementary Figure 3.** Rates of prevalence (A), incidence (B), and YLD (C) of TTH per 100,000 population in adolescents and young adults by country and territory in 2019
- ✧ **Supplementary Figure 4.** Global changing trends of ratios of female YLD rates to male YLD rates of overall headache disorders, migraine, and TTH in adolescents and young adults from 1990 to 2019
- ✧ **Supplementary Figure 5.** The associations between socio-demographic factor and overall headache disorders (A), migraine (B), and TTH (C) burden in adolescents and young adults in 2019 and the results of Spearman test by nation

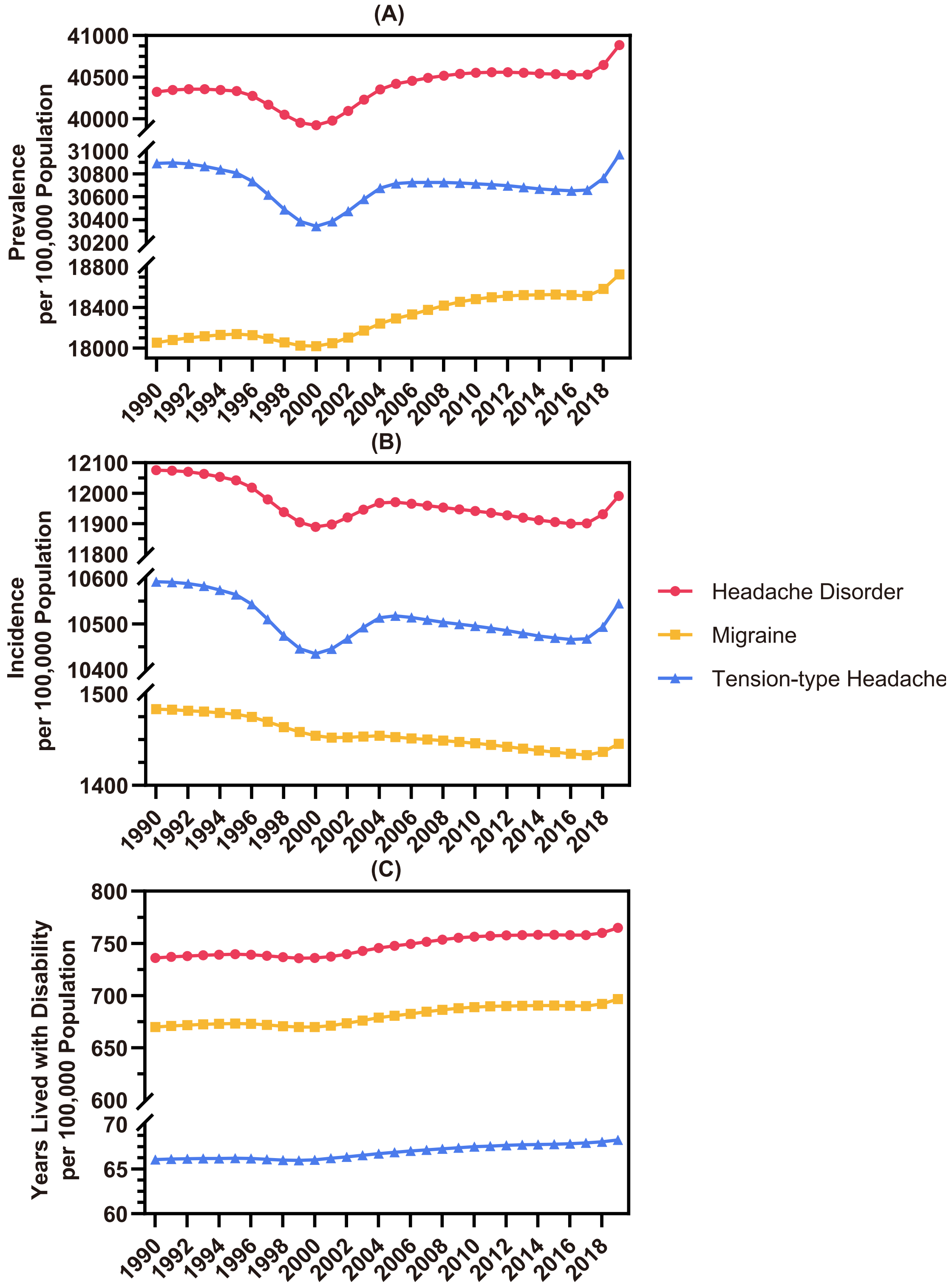

Supplementary Figure 1. Global changing trends of prevalence (A), incidence (B), and YLD (C) of overall headache disorders, migraine, and TTH in adolescents and young adults from 1990 to 2019

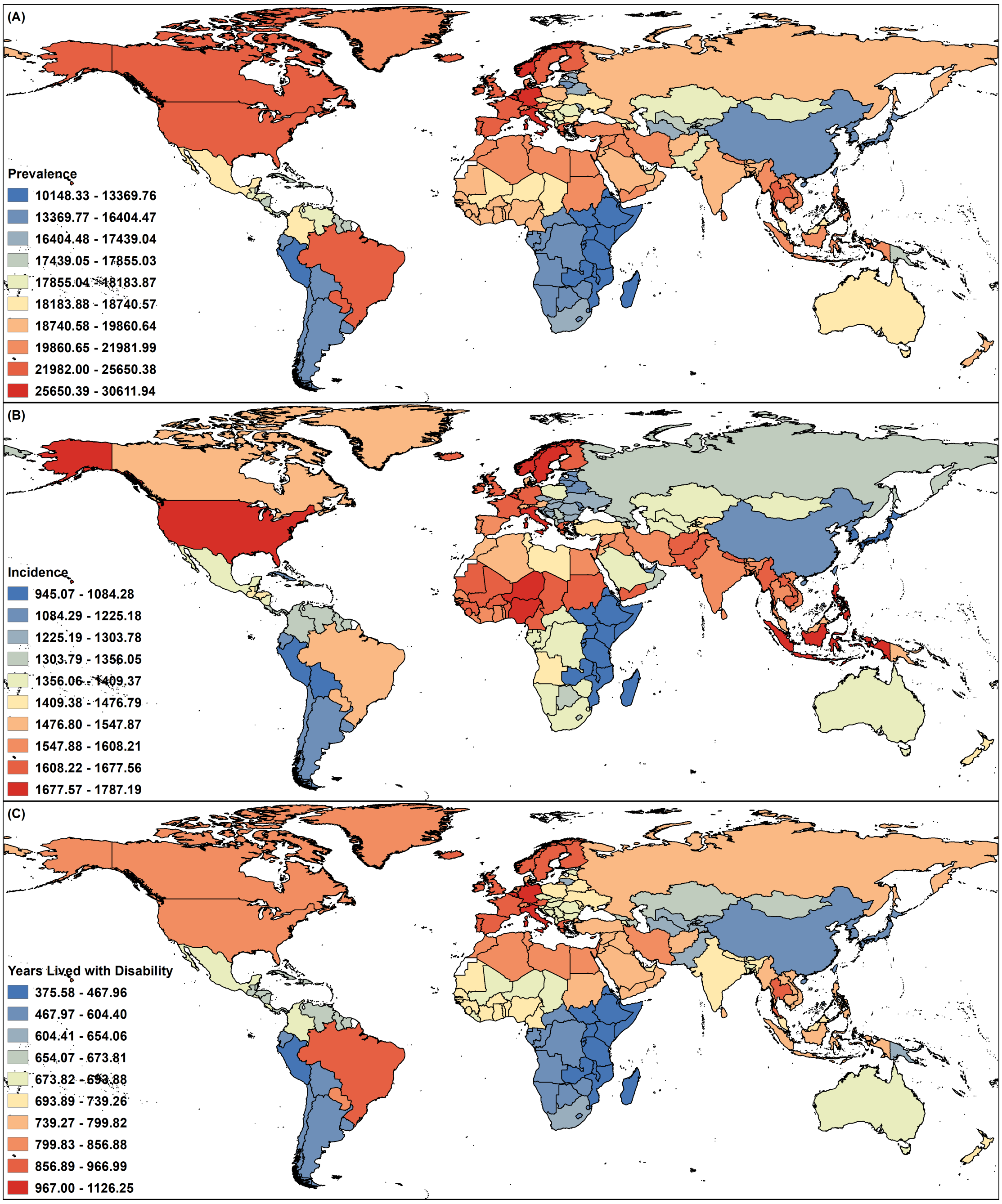

Supplementary Figure 2. Rates of prevalence (A), incidence (B), and YLD (C) of migraine per 100,000 population in adolescents and young adults by country and territory in 2019

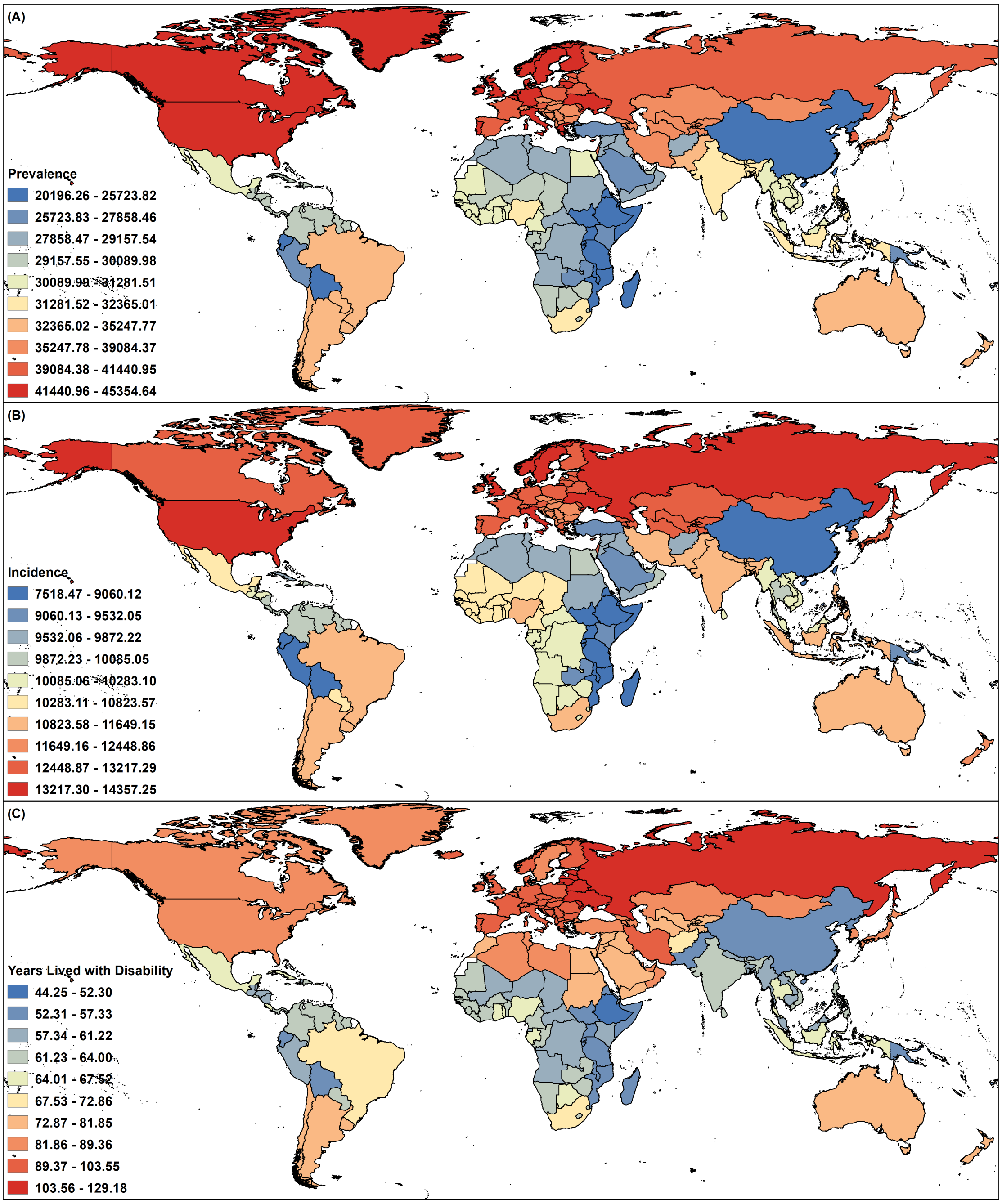

Supplementary Figure 3. Rates of prevalence (A), incidence (B), and YLD (C) of TTH per 100,000 population in adolescents and young adults by country and territory in 2019

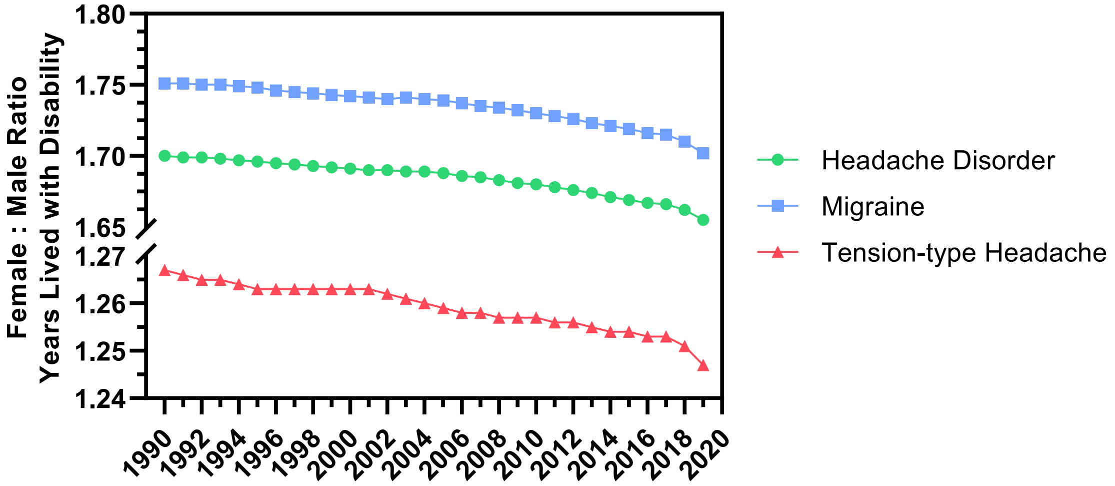

Supplementary Figure 4. Global changing trends of ratios of female YLD rates to male YLD rates of overall headache disorders, migraine, and TTH in adolescents and young adults from 1990 to 2019

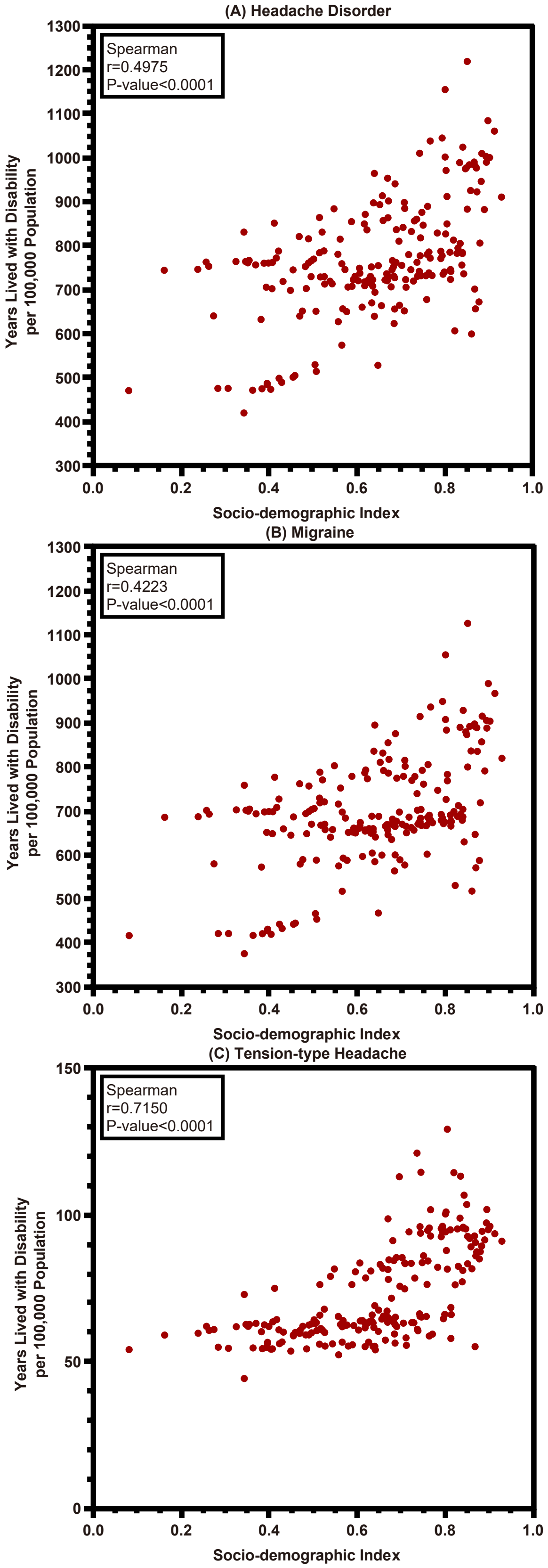

Supplementary Figure 5. The associations between socio-demographic factor and overall headache disorders (A), migraine (B), and TTH (C) burden in adolescents and young adults in 2019 and the results of Spearman test by nation
