# Supplementary material for: Headache disorders and relevant sex and socioeconomic patterns in adolescents and young adults across 204 countries and territories: an updated global analysis
Source: J Headache Pain. 2023 Aug 18;24(1):110. doi: 10.1186/s10194-023-01648-4 (PMC10436621; doi:10.1186/s10194-023-01648-4)
Supplement: Supplementary file 2 — Additional file 2. [file 10194_2023_1648_MOESM2_ESM.pdf]

Supplementary Table

- ✧ **Supplementary Table 1.** Rates of prevalence, incidence, and YLD per 100,000 population of overall headache disorders in adolescents and young adults by geographic region in 2019
- ✧ **Supplementary Table 2.** Rates of prevalence, incidence, and YLD per 100,000 population of migraine in adolescents and young adults by geographic region in 2019
- ✧ **Supplementary Table 3.** Rates of prevalence, incidence, and YLD per 100,000 population of TTH in adolescents and young adults by geographic region in 2019
- ✧ **Supplementary Table 4.** Rates of prevalence, incidence, and YLD per 100,000 population of overall headache disorders in adolescents and young adults by country and territory in 2019
- ✧ **Supplementary Table 5.** Rates of prevalence, incidence, and YLD per 100,000 population of migraine in adolescents and young adults by country and territory in 2019
- ✧ **Supplementary Table 6.** Rates of prevalence, incidence, and YLD per 100,000 population of TTH in adolescents and young adults by country and territory in 2019
- ✧ **Supplementary Table 7.** Global absolute numbers and rates of prevalence, incidence, and YLD of overall headache disorders in adolescents and young adults by sex and age group in 2019
- ✧ **Supplementary Table 8.** Global absolute numbers and rates of prevalence, incidence, and YLD of migraine in adolescents and young adults by sex and age group in 2019
- ✧ **Supplementary Table 9.** Global absolute numbers and rates of prevalence, incidence, and YLD of TTH in adolescents and young adults by sex and age group in 2019
- ✧ **Supplementary Table 10.** Global Changing trends of ratios of female YLD rates to male YLD rates of overall headache disorders, migraine, and TTH in adolescents and young adults from 1990 to 2019
- ✧ **Supplementary Table 11.** Ratios of female YLD rates to male YLD rates of overall headache disorders, migraine, and TTH in adolescents and young adults by country and territory in 2019
- ✧ **Supplementary Table 12.** The YLD rate of overall headache disorders in adolescents and young adults in five major socio-demographic regions from 1990 to 2019
- ✧ **Supplementary Table 13.** The YLD rate of migraine in adolescents and young adults in five major socio-demographic regions from 1990 to 2019
- ✧ **Supplementary Table 14.** The YLD rate of TTH in adolescents and young adults in five major socio-demographic regions from 1990 to 2019
- ✧ **Supplementary Table 15.** The associations between overall headache disorders burden in adolescents and young adults and socio-demographic factor by regions from 1990 to 2019
- ✧ **Supplementary Table 16.** The associations between migraine burden in adolescents and young adults and socio-demographic factor by regions from 1990 to 2019
- ✧ **Supplementary Table 17.** The associations between TTH burden in adolescents and young adults and socio-demographic factor by regions from 1990 to 2019
- ✧ **Supplementary Table 18.** The associations between burden of overall headaches, migraine, and TTH in adolescents and young adults and socio-demographic factor by country and territory in 2019

Supplementary Table 1. Rates of prevalence, incidence, and YLD per 100,000 population of overall headache disorders in adolescents and young adults by geographic region in 2019

Headache Disorders	Prevalence Rate			Incidence Rate			Years Lived with Disability Rate		
	Mean	Upper Limit	Lower Limit	Mean	Upper Limit	Lower Limit	Mean	Upper Limit	Lower Limit
Andean Latin America	34,022.96	38,168.04	30,104.61	9,828.64	11,310.78	8,517.80	567.76	1,216.65	126.92
Australasia	42,719.67	47,013.57	38,599.29	12,939.10	14,854.01	11,405.29	760.31	1,644.52	165.72
Caribbean	39,626.43	43,966.86	35,347.86	11,338.60	12,918.54	9,913.35	727.42	1,613.10	130.96
Central Asia	46,010.72	50,840.52	41,264.76	14,247.62	16,285.93	12,400.51	736.97	1,561.41	156.17
Central Europe	45,921.84	50,339.32	41,567.85	13,944.24	15,867.82	12,230.09	796.34	1,667.32	215.31
Central Latin America	40,353.14	44,381.30	36,372.40	11,750.81	13,317.72	10,333.82	748.12	1,655.33	136.46
Central Sub-Saharan Africa	37,703.78	42,153.54	33,323.00	11,527.94	13,271.90	9,939.30	635.93	1,394.28	120.57
East Asia	32,853.36	35,977.19	29,930.98	8,865.99	10,156.24	7,758.39	656.56	1,433.48	132.18
Eastern Europe	48,546.91	52,838.50	44,358.28	14,906.75	17,057.77	13,035.56	891.19	1,782.94	312.14
Eastern Sub-Saharan Africa	30,824.21	34,682.95	27,080.44	9,773.96	11,228.73	8,417.18	472.47	993.09	115.3
High-income Asia Pacific	43,865.08	47,801.55	40,158.84	13,614.55	15,635.91	11,958.44	659.6	1,380.29	177.23
High-income North America	51,249.51	55,151.30	47,201.75	15,765.23	17,894.74	13,809.30	925.12	2,008.85	175.17
North Africa and Middle East	41,226.10	45,390.10	37,322.63	11,449.20	13,050.97	10,048.61	885.05	1,945.59	196.11
Oceania	38,206.27	42,504.93	34,019.11	11,013.23	12,605.23	9,595.68	707.2	1,612.86	109.86
South Asia	42,404.27	46,208.16	38,591.41	12,934.32	14,580.54	11,397.37	751.93	1,663.86	110.57
Southeast Asia	42,956.73	46,997.85	38,978.34	12,225.38	13,903.16	10,776.71	852.31	1,971.31	126.95
Southern Latin America	40,389.62	44,736.97	36,142.78	12,581.57	14,437.11	10,955.35	659.7	1,386.16	158.65
Southern Sub-Saharan Africa	39,713.50	43,526.85	36,042.32	12,081.76	13,738.15	10,558.48	691.58	1,480.69	146.67
Tropical Latin America	47,337.01	51,476.67	43,748.71	13,125.21	14,904.17	11,534.29	962.21	2,210.89	133.77
Western Europe	52,516.87	57,025.25	47,853.35	15,025.52	17,169.82	13,171.12	1,035.61	2,269.76	198.06
Western Sub-Saharan Africa	41,267.24	45,390.08	37,100.79	12,467.09	14,174.77	10,887.42	772.4	1,720.69	127.09

Back to Top

Supplementary Table 2. Rates of prevalence, incidence, and YLD per 100,000 population of migraine in adolescents and young adults by geographic region in 2019									
**Migraine**	**Prevalence Rate**			**Incidence Rate**			**Years Lived with Disability Rate**		
	**Mean**	**Upper Limit**	**Lower Limit**	**Mean**	**Upper Limit**	**Lower Limit**	**Mean**	**Upper Limit**	**Lower Limit**
Andean Latin America	13,512.15	16,291.17	11,297.37	1,043.75	1,219.84	879.26	509.55	1,125.73	83.92
Australasia	18,552.44	21,796.96	15,729.62	1,381.83	1,607.02	1,169.00	682.56	1,545.44	110.66
Caribbean	17,789.58	21,222.69	14,962.55	1,326.66	1,547.99	1,117.30	664.13	1,544.34	88.75
Central Asia	17,731.94	20,912.71	15,069.54	1,392.30	1,627.20	1,159.99	655	1,484.70	107.71
Central Europe	18,506.45	21,486.82	15,885.81	1,316.38	1,521.61	1,131.63	699.6	1,560.75	143.28
Central Latin America	18,175.84	21,393.00	15,439.47	1,379.54	1,582.41	1,175.46	683	1,582.63	89.98
Central Sub-Saharan Africa	15,644.64	18,926.69	12,938.15	1,405.10	1,652.08	1,152.59	575.41	1,309.18	81.63
East Asia	15,980.89	18,591.35	13,799.60	1,184.04	1,350.48	1,032.32	600.37	1,374.15	86.32
Eastern Europe	18,822.65	21,752.72	16,374.04	1,294.69	1,482.67	1,134.63	765.18	1,603.45	208.09
Eastern Sub-Saharan Africa	11,151.59	13,270.94	9,358.21	1,030.60	1,196.75	847.65	419.07	939.57	78.51
High-income Asia Pacific	15,134.25	17,478.67	13,063.86	1,035.29	1,186.83	893.66	574.1	1,290.86	119.75
High-income North America	22,885.47	26,494.90	19,867.81	1,672.82	1,889.86	1,451.27	836.17	1,916.41	110.73
North Africa and Middle East	20,504.08	23,988.98	17,522.88	1,524.95	1,761.24	1,288.64	802.03	1,848.36	132.25
Oceania	17,750.32	21,036.85	14,837.68	1,515.58	1,781.23	1,256.74	652.12	1,563.52	73.28
South Asia	19,230.05	22,215.83	16,530.70	1,589.46	1,799.91	1,368.63	691.28	1,604.83	75.19
Southeast Asia	21,005.49	24,565.60	17,985.34	1,648.37	1,878.10	1,415.20	788.53	1,935.90	83.36
Southern Latin America	15,575.45	18,417.92	13,104.64	1,213.48	1,417.36	1,008.42	584.45	1,304.21	107.64
Southern Sub-Saharan Africa	16,843.16	19,722.35	14,391.08	1,373.77	1,576.16	1,168.19	622.51	1,401.52	99.1
Tropical Latin America	24,297.39	28,908.58	20,813.95	1,528.09	1,715.54	1,339.31	893.38	2,144.61	86.76
Western Europe	25,256.84	29,406.73	21,788.36	1,645.29	1,892.35	1,419.35	939.14	2,176.86	133.5
Western Sub-Saharan Africa	19,178.61	22,879.24	16,165.61	1,671.49	1,935.45	1,391.42	708.41	1,650.41	87.27

Back to Top

Supplementary Table 3. Rates of prevalence, incidence, and YLD per 100,000 population of TTH in adolescents and young adults by geographic region in 2019									
**TTH**	**Prevalence Rate**			**Incidence Rate**			**Years Lived with Disability Rate**		
	**Mean**	**Upper Limit**	**Lower Limit**	**Mean**	**Upper Limit**	**Lower Limit**	**Mean**	**Upper Limit**	**Lower Limit**
Andean Latin America	25,921.19	30,594.76	21,466.35	8,784.89	10,193.89	7,504.09	58.21	230.81	17.49
Australasia	33,826.81	39,487.00	28,772.78	11,557.27	13,425.81	10,032.46	77.76	248.36	22.94
Caribbean	29,743.95	34,870.78	24,710.77	10,011.94	11,566.94	8,622.62	63.29	247.83	17.94
Central Asia	38,842.73	45,443.75	32,983.63	12,855.32	14,892.61	11,068.46	81.97	276.62	21.83
Central Europe	38,216.88	44,023.05	32,715.05	12,627.86	14,581.09	10,937.57	96.74	294.03	30.02
Central Latin America	30,473.10	35,316.24	25,751.56	10,371.27	11,913.79	8,977.00	65.11	253.73	18.61
Central Sub-Saharan Africa	29,133.40	34,622.62	24,129.84	10,122.85	11,820.76	8,588.87	60.53	238.5	17
East Asia	22,251.21	25,821.48	19,047.98	7,681.95	8,968.60	6,581.44	56.19	192.08	18.07
Eastern Europe	41,123.46	46,715.35	35,681.41	13,612.06	15,789.11	11,732.37	126.01	360.32	42.91
Eastern Sub-Saharan Africa	24,077.61	28,658.70	19,920.05	8,743.37	10,174.76	7,469.76	53.4	204.84	15.66
High-income Asia Pacific	38,508.49	43,779.72	33,604.62	12,579.26	14,599.58	10,891.19	85.5	277.27	24.18
High-income North America	43,035.48	48,793.17	37,454.91	14,092.41	16,186.16	12,189.67	88.95	298.56	23.91
North Africa and Middle East	29,424.51	34,379.57	24,800.18	9,924.25	11,484.08	8,554.12	83.02	235.21	27.53
Oceania	27,779.23	33,160.91	22,899.06	9,497.65	11,030.33	8,072.39	55.08	225.47	14.95
South Asia	32,437.83	37,279.73	27,906.58	11,344.86	12,959.29	9,837.90	60.65	245.07	15.51
Southeast Asia	31,605.40	36,434.98	27,008.21	10,577.01	12,190.79	9,160.90	63.78	248.41	17.15
Southern Latin America	33,104.03	38,848.60	27,726.55	11,368.09	13,204.41	9,729.58	75.25	243.65	21.53
Southern Sub-Saharan Africa	30,946.77	35,683.08	26,321.82	10,707.99	12,349.00	9,201.09	69.07	245.33	20.62
Tropical Latin America	35,182.70	40,011.84	30,618.81	11,597.13	13,350.50	10,045.12	68.83	271.17	18.1
Western Europe	42,375.95	48,383.14	36,386.52	13,380.23	15,472.06	11,538.25	96.47	320.28	27.21
Western Sub-Saharan Africa	30,978.22	36,133.22	26,152.79	10,795.60	12,487.84	9,264.19	64	252.85	18.01

Back to Top

Supplementary Table 4. Rates of prevalence, incidence, and YLD per 100,000 population of overall headache disorders in adolescents and young adults by country and territory in 2019									
**Location**	**Prevalence Rate**			**Incidence Rate**			**Years Lived with Disability Rate**		
	**Mean**	**Upper Limit**	**Lower Limit**	**Mean**	**Upper Limit**	**Lower Limit**	**Mean**	**Upper Limit**	**Lower Limit**
Afghanistan	39,813.10	44,426.45	35,338.55	11,436.07	13,056.11	9,853.80	831.06	1,883.72	170.53
Albania	45,198.67	49,861.46	40,294.55	13,768.38	15,752.20	12,096.96	766.29	1,625.63	200.97
Algeria	40,715.83	45,046.66	36,677.00	11,232.70	12,829.59	9,824.76	893.41	1,954.16	198.82
American Samoa	37,871.85	42,151.03	33,674.11	10,856.41	12,473.76	9,462.19	705.81	1,617.79	108.98
Andorra	51,818.13	56,837.93	46,739.73	14,431.02	16,636.56	12,600.56	1,003.08	2,185.28	200.01
Angola	37,641.69	42,102.80	33,386.09	11,532.22	13,299.52	9,948.61	639.99	1,411.92	121.05
Antigua and Barbuda	39,748.47	44,013.56	35,432.12	11,253.28	12,844.06	9,819.71	740.91	1,653.22	139.02
Argentina	40,251.77	44,614.17	35,914.21	12,594.12	14,466.73	10,934.35	652.00	1,373.48	158.51
Armenia	46,515.32	51,422.51	41,818.45	14,170.33	16,285.09	12,286.29	762.14	1,622.01	167.01
Australia	42,540.52	46,920.39	38,278.15	12,843.64	14,782.19	11,299.90	755.99	1,643.08	162.68
Austria	50,745.51	55,688.87	45,715.93	14,553.01	16,754.70	12,636.44	977.14	2,074.97	224.54
Azerbaijan	46,322.29	51,111.52	41,553.00	14,177.79	16,289.44	12,326.10	751.91	1,617.84	165.17
Bahamas	39,662.21	44,028.88	35,387.90	11,305.66	12,883.05	9,885.69	735.65	1,631.27	133.07
Bahrain	40,217.60	44,646.11	35,988.99	11,047.12	12,782.36	9,616.39	846.86	1,795.01	206.58
Bangladesh	42,106.63	46,728.84	37,650.15	12,455.27	14,233.53	10,815.19	752.95	1,710.16	104.79
Barbados	39,670.36	44,014.48	35,367.36	11,197.47	12,818.66	9,785.55	741.95	1,646.56	141.43
Belarus	46,944.06	51,869.20	42,320.41	14,099.75	16,174.56	12,279.72	817.80	1,650.61	276.29
Belgium	54,848.79	59,482.41	50,159.47	14,761.50	16,920.76	12,916.91	1,218.92	2,787.45	189.25
Belize	39,526.98	43,974.85	35,064.21	11,470.99	13,096.57	9,979.53	720.60	1,641.55	123.45
Benin	40,729.51	45,223.87	36,184.82	12,110.39	13,807.92	10,502.10	761.45	1,725.40	120.69
Bermuda	39,624.48	43,854.92	35,452.36	11,093.32	12,705.55	9,697.98	745.91	1,631.03	148.13
Bhutan	42,145.55	46,689.16	37,706.83	12,481.12	14,260.07	10,859.71	745.27	1,706.72	102.41
Bolivia (Plurinational State of)	33,922.74	38,261.57	29,873.97	9,833.58	11,340.06	8,532.99	574.12	1,228.09	124.71
Bosnia and Herzegovina	45,323.22	50,115.44	40,643.63	13,628.27	15,611.06	11,986.42	780.00	1,616.57	211.65
Botswana	38,857.82	43,204.39	34,727.65	11,616.84	13,326.51	10,055.90	670.28	1,464.28	137.41
Brazil	47,408.87	51,549.37	43,841.65	13,149.88	14,939.86	11,555.94	964.31	2,218.11	134.46
Brunei Darussalam	42,153.80	46,718.93	37,701.06	13,374.35	15,408.51	11,603.98	606.92	1,291.65	149.29
Bulgaria	45,310.90	50,046.89	40,653.76	13,577.04	15,566.25	11,879.48	785.71	1,631.82	214.02
Burkina Faso	40,764.73	45,218.06	36,203.66	12,097.73	13,797.02	10,504.87	763.11	1,724.36	120.83
Burundi	31,782.52	36,012.87	27,692.42	10,017.09	11,513.45	8,583.16	476.10	998.08	115.04
Cabo Verde	41,837.41	46,192.33	37,503.82	12,188.97	13,922.72	10,564.04	788.44	1,744.27	138.46
Cambodia	42,184.13	46,776.35	37,835.50	11,912.61	13,623.39	10,351.25	821.07	1,907.02	111.34
Cameroon	40,835.34	45,285.00	36,413.57	12,130.22	13,855.45	10,556.46	760.94	1,707.17	120.54
Canada	50,371.67	55,033.24	45,534.30	14,622.02	16,690.00	12,743.60	922.72	2,032.83	169.10

Back to Top

Central African Republic	37,830.00	42,281.63	33,485.90	11,506.01	13,260.49	9,935.56	640.39	1,394.12	122.64
Chad	40,260.56	44,927.41	35,552.03	12,052.59	13,748.54	10,394.50	746.32	1,691.13	115.29
Chile	40,727.32	45,214.21	36,560.34	12,556.82	14,454.36	10,944.91	678.01	1,426.90	161.21
China	32,860.13	36,006.09	29,966.28	8,870.67	10,167.97	7,757.09	656.41	1,435.91	132.49
Colombia	39,984.88	44,370.22	35,644.46	11,355.81	12,942.21	9,943.50	751.83	1,678.03	131.53
Comoros	32,264.54	36,305.91	28,318.47	9,980.10	11,476.50	8,659.76	501.43	1,043.38	130.87
Congo	38,224.37	42,537.47	34,098.65	11,527.04	13,257.77	9,977.13	656.81	1,424.44	129.89
Cook Islands	38,454.75	42,516.34	34,429.36	10,805.80	12,395.75	9,475.65	738.46	1,691.96	121.10
Costa Rica	39,910.98	44,154.58	35,548.95	11,367.37	12,968.80	9,942.42	745.45	1,653.29	132.14
Cote d'Ivoire	41,078.93	45,496.06	36,659.71	12,143.30	13,835.60	10,588.92	761.90	1,716.73	126.89
Croatia	45,282.83	49,867.87	40,805.26	13,664.66	15,646.85	11,986.86	777.54	1,627.26	209.37
Cuba	39,497.07	43,865.71	35,151.34	11,080.76	12,780.74	9,693.82	736.34	1,625.61	144.92
Cyprus	52,454.01	57,407.44	47,352.68	14,784.02	16,978.76	12,826.45	1,024.13	2,219.90	192.22
Czechia	45,308.58	50,040.29	40,580.36	13,573.66	15,569.17	11,900.43	782.87	1,613.58	213.51
Democratic People's Republic of Korea	31,982.22	35,531.66	28,563.11	8,708.73	9,967.84	7,604.07	627.50	1,382.19	120.53
Democratic Republic of the Congo	37,673.64	42,132.91	33,267.86	11,527.59	13,268.61	9,940.78	632.65	1,390.72	120.71
Denmark	50,942.69	55,752.49	45,951.63	14,553.31	16,806.75	12,684.29	882.54	1,933.11	176.90
Djibouti	32,474.87	36,491.82	28,595.61	10,033.43	11,540.04	8,680.45	504.99	1,054.17	133.55
Dominica	39,390.88	43,680.64	35,078.46	11,289.65	12,881.85	9,882.46	719.57	1,596.45	131.40
Dominican Republic	39,593.53	43,919.64	35,321.38	11,399.17	13,014.40	9,934.13	723.29	1,612.16	125.47
Ecuador	33,804.31	37,813.26	30,109.13	9,615.47	11,041.23	8,366.18	639.82	1,418.28	122.92
Egypt	42,820.79	46,910.76	38,730.82	11,641.68	13,241.56	10,190.49	913.50	2,019.30	187.48
El Salvador	39,968.61	44,413.62	35,545.20	11,439.70	13,040.22	9,949.41	746.02	1,652.35	126.25
Equatorial Guinea	37,427.45	42,079.29	33,055.44	11,533.29	13,259.90	9,909.16	623.08	1,371.62	117.33
Eritrea	31,928.08	36,053.11	27,864.91	10,004.03	11,508.84	8,598.79	484.30	1,009.35	120.16
Estonia	46,784.09	51,648.58	42,255.49	14,088.28	16,161.15	12,258.99	805.36	1,627.41	271.53
Eswatini	38,258.66	42,680.19	34,014.79	11,591.15	13,374.57	10,001.17	650.13	1,418.35	124.36
Ethiopia	27,037.82	30,449.47	23,825.43	8,822.45	10,220.05	7,614.27	419.84	906.58	92.95
Fiji	38,381.72	42,488.98	34,453.17	10,933.10	12,519.53	9,563.63	722.45	1,639.98	116.16
Finland	51,502.68	56,331.95	46,502.31	14,675.34	16,805.86	12,816.48	984.06	2,140.83	184.11
France	50,054.20	54,724.59	45,476.88	14,615.76	16,667.49	12,809.17	988.84	2,130.79	215.32
Gabon	38,416.79	42,677.45	34,162.57	11,539.23	13,250.52	10,005.78	663.81	1,446.93	133.74
Gambia	40,842.93	45,437.87	36,361.17	12,129.07	13,840.89	10,516.14	760.67	1,715.19	120.53
Georgia	46,171.10	51,011.23	41,507.77	14,013.97	16,109.78	12,184.01	755.16	1,596.78	173.24
Germany	53,367.27	57,828.60	48,597.92	14,693.50	16,925.89	12,845.06	1,084.22	2,393.93	196.59
Ghana	41,378.80	45,787.66	37,078.29	12,156.54	13,864.38	10,586.45	780.76	1,742.78	132.80
Greece	52,429.27	57,313.86	47,359.72	14,578.14	16,710.06	12,717.18	1,044.79	2,306.36	199.70

Back to Top

Greenland	49,898.15	54,491.85	45,041.76	14,655.05	16,722.30	12,791.07	889.53	1,977.99	159.00
Grenada	39,412.55	43,753.35	35,021.11	11,263.33	12,887.57	9,848.19	720.70	1,625.08	132.92
Guam	38,123.59	42,284.91	34,124.16	10,791.24	12,374.07	9,438.75	723.38	1,645.89	118.64
Guatemala	39,785.17	44,372.08	35,307.29	11,543.16	13,177.32	9,956.71	729.76	1,634.21	115.52
Guinea	40,781.00	45,356.55	36,215.85	12,121.23	13,835.04	10,512.19	764.37	1,718.87	126.99
Guinea-Bissau	41,042.97	45,609.45	36,550.32	12,171.66	13,895.08	10,576.80	766.83	1,721.07	124.31
Guyana	39,599.46	44,004.16	35,188.80	11,394.58	13,017.62	9,935.92	718.23	1,611.68	124.84
Haiti	39,740.75	44,264.47	35,285.16	11,532.68	13,103.92	10,013.37	719.09	1,622.70	120.31
Honduras	39,725.48	44,145.82	35,261.33	11,533.25	13,207.15	9,969.47	729.79	1,632.85	118.85
Hungary	45,372.01	50,010.74	40,633.78	13,570.21	15,536.19	11,922.24	787.35	1,642.21	211.86
Iceland	51,389.90	56,206.75	46,425.00	14,736.11	16,805.46	12,871.22	981.86	2,153.12	176.81
India	42,430.43	46,176.47	38,634.37	12,944.51	14,626.35	11,397.83	759.22	1,674.20	116.59
Indonesia	43,329.07	47,101.65	39,616.65	12,572.26	14,267.48	11,154.45	857.40	1,987.84	129.06
Iran (Islamic Republic of)	46,538.39	50,571.02	42,717.38	12,708.28	14,515.41	11,096.48	953.55	2,028.05	226.48
Iraq	40,336.55	44,965.91	36,096.63	11,334.61	12,924.46	9,871.50	863.73	1,925.40	183.38
Ireland	51,467.75	56,401.41	46,471.39	14,688.95	16,780.59	12,818.73	990.51	2,164.83	186.24
Israel	50,854.51	55,623.19	45,726.13	14,822.66	16,892.78	12,991.71	971.34	2,144.50	168.56
Italy	56,532.39	60,804.41	52,063.66	16,037.79	18,353.99	14,070.07	1,155.09	2,627.47	198.03
Jamaica	39,694.97	44,076.05	35,287.94	11,373.94	12,981.99	9,937.39	730.76	1,647.40	130.71
Japan	43,045.36	46,888.97	39,407.81	13,756.58	15,818.97	12,079.74	656.84	1,378.40	182.15
Jordan	40,130.04	44,607.83	35,957.50	11,293.26	12,885.93	9,877.98	856.65	1,907.38	184.47
Kazakhstan	46,164.42	50,942.17	41,504.86	14,184.76	16,289.52	12,315.88	745.81	1,593.63	161.96
Kenya	33,334.20	36,794.31	29,971.97	10,557.92	12,027.17	9,140.37	514.33	1,084.55	131.83
Kiribati	38,363.24	42,705.54	34,213.04	11,068.50	12,631.37	9,652.84	712.90	1,622.24	107.74
Kuwait	40,821.83	45,372.57	36,778.71	11,102.01	12,756.10	9,710.53	883.15	1,919.60	199.41
Kyrgyzstan	45,843.56	50,716.42	41,031.39	14,308.03	16,367.16	12,451.35	730.52	1,556.29	153.25
Lao People's Democratic Republic	42,062.77	46,562.85	37,579.10	11,910.61	13,613.30	10,350.93	815.85	1,891.25	113.31
Latvia	46,788.30	51,558.96	42,220.71	14,041.34	16,139.73	12,233.94	812.97	1,628.27	273.53
Lebanon	40,842.16	45,189.08	36,692.31	11,223.81	12,844.85	9,823.58	898.41	1,982.94	194.70
Lesotho	38,427.44	42,856.26	34,278.02	11,587.12	13,296.64	10,014.29	651.35	1,414.30	126.76
Liberia	40,974.14	45,406.82	36,463.46	12,094.13	13,792.58	10,539.44	756.63	1,699.65	126.42
Libya	40,678.16	44,942.29	36,538.74	11,174.27	12,776.04	9,805.51	885.00	1,955.68	202.11
Lithuania	47,723.82	52,199.13	43,106.84	14,049.70	16,313.38	12,266.56	736.15	1,486.08	230.47
Luxembourg	50,940.21	55,978.55	46,009.02	14,639.76	16,828.70	12,734.60	989.93	2,108.03	221.83
Madagascar	31,902.69	36,069.68	27,849.76	9,994.32	11,479.07	8,587.31	487.48	1,036.63	120.18
Malawi	31,622.97	35,891.33	27,529.43	10,001.63	11,505.53	8,554.19	475.15	1,010.75	114.95
Malaysia	41,044.06	45,511.52	36,733.45	11,742.65	13,482.70	10,190.32	762.47	1,756.79	116.75
Maldives	42,160.61	46,913.46	37,816.92	11,777.98	13,625.28	10,067.54	814.82	1,868.13	122.14
Mali	40,425.94	45,065.37	35,846.62	12,057.66	13,758.94	10,416.55	753.11	1,695.30	118.58
Malta	51,982.05	56,752.14	46,995.07	14,672.11	16,824.92	12,767.78	1,001.85	2,178.39	192.94
Marshall Islands	38,295.61	42,614.54	34,328.46	11,007.63	12,608.13	9,585.45	713.41	1,630.84	111.31

Mauritania	40,745.65	45,225.19	36,231.52	12,083.70	13,793.03	10,504.01	765.67	1,718.20	125.69
Mauritius	42,399.66	46,768.07	38,044.26	11,615.55	13,287.79	10,134.59	841.81	1,946.53	124.43
Mexico	40,863.26	44,541.91	37,145.78	12,092.22	13,648.17	10,608.08	754.97	1,654.45	143.29
Micronesia (Federated States of)	38,000.02	42,244.41	33,761.85	10,939.54	12,558.28	9,536.63	706.37	1,608.99	110.28
Monaco	51,639.50	56,706.56	46,589.26	14,461.72	16,626.46	12,632.55	1,000.27	2,184.09	199.41
Mongolia	46,236.26	51,003.31	41,540.07	14,194.05	16,349.41	12,303.51	749.00	1,603.95	165.33
Montenegro	45,242.34	49,981.93	40,498.89	13,721.27	15,716.36	12,038.78	771.11	1,608.45	203.36
Morocco	40,551.79	44,814.71	36,490.75	11,227.35	12,805.59	9,829.07	884.17	1,952.18	192.54
Mozambique	31,667.38	35,921.80	27,556.26	9,998.50	11,510.05	8,544.13	475.74	1,010.73	116.24
Myanmar	42,263.82	46,793.44	37,942.63	11,822.81	13,518.94	10,322.29	831.25	1,922.07	117.47
Namibia	38,355.37	42,732.96	34,163.55	11,561.25	13,286.19	10,008.72	660.61	1,436.89	131.79
Nauru	38,135.87	42,578.62	33,930.58	11,057.94	12,670.34	9,607.98	709.12	1,617.39	108.80
Nepal	42,836.15	47,313.49	38,277.08	12,571.57	14,321.16	10,976.41	787.87	1,785.91	114.37
Netherlands	53,523.27	58,653.22	48,326.10	14,618.24	16,878.23	12,579.92	946.24	2,126.31	160.51
New Zealand	43,729.67	47,458.26	40,151.36	13,477.29	15,245.87	11,869.32	784.67	1,672.49	177.43
Nicaragua	39,691.74	44,047.08	35,294.33	11,472.95	13,081.00	9,975.42	729.09	1,627.21	121.51
Niger	40,129.32	44,898.85	35,450.69	12,044.40	13,757.47	10,385.56	744.39	1,688.93	114.43
Nigeria	41,775.15	45,541.90	38,099.44	12,865.98	14,607.77	11,262.88	784.21	1,727.73	135.00
Niue	38,180.53	42,296.44	34,197.38	10,765.21	12,315.25	9,434.67	723.33	1,647.50	120.64
North Macedonia	45,376.50	50,140.28	40,693.65	13,700.53	15,739.96	11,977.91	777.19	1,631.03	210.24
Northern Mariana Islands	37,983.58	42,147.93	33,925.78	10,436.31	12,037.33	9,088.22	732.36	1,672.74	124.25
Norway	55,186.54	59,438.86	50,950.56	16,144.44	18,430.48	13,963.98	1,060.60	2,337.46	175.16
Oman	39,995.29	44,728.12	35,611.63	11,224.96	13,035.75	9,598.23	829.10	1,776.52	194.32
Pakistan	42,394.53	46,264.96	38,576.47	13,275.66	15,045.38	11,658.88	698.59	1,581.28	79.87
Palau	38,230.29	42,312.71	34,259.34	10,571.16	12,204.29	9,222.54	730.80	1,623.05	130.84
Palestine	40,173.86	44,746.55	35,830.52	11,394.26	12,963.93	9,896.69	854.94	1,934.69	178.74
Panama	39,527.62	43,864.66	35,194.03	11,364.99	12,968.25	9,938.04	727.34	1,613.90	129.20
Papua New Guinea	38,222.33	42,546.71	34,010.29	11,026.84	12,630.69	9,598.58	705.94	1,608.89	109.26
Paraguay	45,121.50	49,770.27	40,674.64	12,364.55	14,129.54	10,746.51	897.45	2,079.25	112.56
Peru	34,170.56	38,316.88	30,009.81	9,937.37	11,438.10	8,618.65	528.25	1,108.06	127.85
Philippines	43,036.23	46,836.91	39,223.73	12,694.12	14,389.28	11,180.74	836.05	1,948.63	121.62
Poland	47,179.53	51,347.32	43,308.96	14,587.17	16,635.14	12,767.38	826.96	1,754.55	230.36
Portugal	51,804.91	56,738.90	46,781.50	14,544.95	16,688.28	12,716.89	1,010.38	2,207.62	196.27
Puerto Rico	39,681.72	44,068.23	35,336.60	11,209.27	12,843.30	9,782.82	741.66	1,638.50	138.03
Qatar	39,429.45	44,199.83	35,116.06	11,065.79	12,976.77	9,378.11	794.84	1,676.33	195.95
Republic of Korea	45,779.81	49,836.32	41,792.70	13,382.82	15,443.80	11,586.05	672.45	1,424.36	168.27
Republic of Moldova	46,981.00	51,950.25	42,368.54	14,166.10	16,284.85	12,298.95	810.37	1,628.13	269.58
Romania	45,138.22	49,877.69	40,461.60	13,570.38	15,564.02	11,920.29	780.35	1,615.07	212.05

Back to Top

Russian Federation	48,719.75	53,095.73	44,470.50	14,970.81	17,173.66	13,109.49	911.91	1,811.38	320.59
Rwanda	32,034.18	36,160.67	28,070.06	10,024.78	11,494.71	8,638.13	489.40	1,033.67	123.14
Saint Kitts and Nevis	39,588.41	43,842.43	35,279.74	11,248.40	12,845.20	9,826.66	732.16	1,629.07	135.39
Saint Lucia	39,649.79	43,972.38	35,416.50	11,233.79	12,820.13	9,797.04	732.62	1,636.73	134.25
Saint Vincent and the Grenadines	39,478.43	43,924.68	35,160.45	11,277.19	12,851.46	9,865.27	723.30	1,602.88	132.25
Samoa	37,668.15	41,919.32	33,361.16	10,969.90	12,575.76	9,520.43	694.40	1,595.11	106.45
San Marino	51,753.14	56,504.60	46,804.29	14,627.48	16,757.45	12,818.77	1,009.81	2,213.48	192.80
Sao Tome and Principe	41,010.95	45,440.37	36,560.20	12,097.06	13,783.01	10,565.61	769.55	1,716.25	130.97
Saudi Arabia	38,547.63	42,863.30	34,739.65	10,806.92	12,344.44	9,410.01	849.83	1,809.31	199.44
Senegal	40,790.47	45,259.55	36,248.67	12,086.78	13,801.24	10,502.12	760.41	1,708.64	126.62
Serbia	45,223.95	49,987.24	40,509.44	13,707.75	15,708.87	12,007.41	771.81	1,614.91	202.97
Seychelles	42,132.91	46,461.44	37,893.00	11,598.23	13,330.54	10,086.14	832.56	1,914.97	124.41
Sierra Leone	40,994.09	45,497.10	36,489.09	12,137.79	13,873.83	10,530.16	764.21	1,718.35	123.61
Singapore	41,649.12	46,444.26	37,299.49	13,160.50	15,220.12	11,360.04	599.58	1,228.52	173.05
Slovakia	45,527.30	50,348.87	40,845.23	13,664.90	15,667.34	11,916.23	785.52	1,637.90	210.46
Slovenia	45,327.15	50,068.42	40,611.47	13,569.52	15,549.07	11,871.72	782.32	1,628.95	212.35
Solomon Islands	38,046.05	42,407.43	33,883.58	11,040.52	12,637.48	9,577.21	702.40	1,607.34	108.25
Somalia	31,681.63	35,976.47	27,556.53	10,007.76	11,501.27	8,542.49	470.75	985.01	112.45
South Africa	40,323.01	44,023.38	36,791.33	12,288.29	13,987.63	10,742.28	706.52	1,508.25	155.06
South Sudan	31,505.59	35,842.59	27,467.55	9,928.81	11,432.53	8,521.50	471.78	992.93	117.84
Spain	51,298.05	56,245.15	46,420.28	14,518.87	16,682.91	12,576.17	1,037.98	2,259.37	221.54
Sri Lanka	42,268.37	46,730.91	37,892.01	11,745.08	13,439.42	10,243.83	836.47	1,938.20	122.91
Sudan	40,228.04	44,863.51	35,857.02	11,416.32	13,000.38	9,902.84	864.34	1,934.08	176.19
Suriname	39,496.99	43,898.33	35,186.00	11,312.97	12,877.13	9,898.72	722.78	1,620.91	130.17
Sweden	51,882.74	55,866.75	47,987.84	15,797.43	18,066.83	13,880.01	976.55	2,156.59	166.64
Switzerland	48,258.53	52,929.47	43,605.29	14,397.36	16,448.22	12,467.04	910.83	1,960.36	192.06
Syrian Arab Republic	40,282.13	44,656.68	36,063.17	11,247.96	12,871.55	9,774.62	871.42	1,965.28	184.12
Taiwan (Province of China)	33,482.26	37,223.04	30,063.43	8,761.32	10,079.51	7,621.27	701.53	1,559.05	128.35
Tajikistan	45,675.34	50,630.88	40,820.99	14,372.07	16,446.27	12,483.63	719.18	1,573.11	146.09
Thailand	44,004.03	48,591.43	39,630.78	11,603.97	13,321.73	10,110.41	940.89	2,194.22	133.83
Timor-Leste	41,335.35	46,097.35	36,578.30	11,931.00	13,622.32	10,317.52	785.12	1,850.66	101.94
Togo	41,125.24	45,489.03	36,745.18	12,108.29	13,797.73	10,567.60	772.20	1,735.54	127.91
Tokelau	38,091.09	42,219.31	34,035.12	10,889.39	12,487.51	9,528.13	716.24	1,626.78	114.84
Tonga	37,974.32	42,260.14	33,784.65	10,977.61	12,581.53	9,540.76	708.26	1,618.05	108.68
Trinidad and Tobago	39,675.35	43,961.37	35,364.86	11,284.49	12,935.74	9,879.33	731.86	1,613.90	135.17
Tunisia	40,801.45	45,012.50	36,668.75	11,157.99	12,741.78	9,740.64	902.00	1,951.72	205.48

Back to Top

Turkey	38,619.16	42,764.86	34,908.71	10,779.32	12,351.81	9,430.81	876.00	1,895.12	217.24
Turkmenistan	45,798.06	50,635.48	41,056.05	14,212.82	16,231.50	12,373.45	727.18	1,535.55	154.06
Tuvalu	38,022.34	42,306.96	33,942.07	10,926.45	12,542.59	9,551.87	707.73	1,618.29	111.27
Uganda	31,525.35	35,809.35	27,353.95	9,988.80	11,513.26	8,535.75	473.68	1,011.85	116.88
Ukraine	48,623.56	52,814.42	44,662.16	15,040.31	17,278.31	13,096.44	860.35	1,745.20	290.29
United Arab Emirates	39,823.74	44,793.80	35,459.73	11,041.99	13,025.76	9,279.04	806.09	1,704.18	207.77
United Kingdom	51,968.07	56,011.95	47,729.96	15,654.41	17,947.68	13,798.98	975.02	2,111.06	190.94
United Republic of Tanzania	32,072.37	36,196.55	28,000.47	10,013.75	11,505.32	8,591.17	498.76	1,057.50	122.75
United States of America	51,344.20	55,333.79	47,401.02	15,888.42	18,016.46	13,912.78	925.38	2,013.08	175.10
United States Virgin Islands	39,571.90	43,800.66	35,273.22	11,198.31	12,836.85	9,775.75	740.65	1,655.96	141.58
Uruguay	40,425.73	44,690.04	36,268.98	12,544.90	14,410.58	10,964.27	664.77	1,398.43	158.96
Uzbekistan	45,913.80	50,853.72	41,107.98	14,293.73	16,335.20	12,442.61	730.52	1,542.10	152.08
Vanuatu	38,071.17	42,471.63	33,881.33	11,072.75	12,663.57	9,621.38	702.71	1,600.62	107.09
Venezuela (Bolivarian Republic of)	39,691.24	43,993.73	35,362.11	11,338.61	12,878.84	9,925.26	737.64	1,660.04	131.18
Viet Nam	42,514.15	46,894.24	38,204.96	11,745.25	13,492.66	10,200.38	849.63	1,949.24	124.16
Yemen	40,143.03	44,923.77	35,721.49	11,466.61	13,037.42	9,933.87	851.57	1,910.71	176.27
Zambia	33,584.88	38,019.89	29,280.76	10,254.22	11,846.15	8,792.60	529.68	1,104.17	142.19
Zimbabwe	38,026.06	42,568.33	33,832.16	11,557.70	13,311.03	9,976.33	651.60	1,426.43	125.79

Back to Top

Supplementary Table 5. Rates of prevalence, incidence, and YLD per 100,000 population of migraine in adolescents and young adults by country and territory in 2019									
Location	Prevalence Rate			Incidence Rate			Years Lived with Disability Rate		
	Mean	Upper Limit	Lower Limit	Mean	Upper Limit	Lower Limit	Mean	Upper Limit	Lower Limit
Afghanistan	19,772.41	23,842.01	16,349.11	1,641.69	1,925.51	1,343.49	758.20	1,802.00	113.64
Albania	17,949.40	20,987.68	15,291.26	1,319.52	1,541.47	1,114.48	675.08	1,506.23	132.17
Algeria	20,670.47	24,336.31	17,485.00	1,506.10	1,756.41	1,259.04	810.33	1,865.00	132.36
American Samoa	17,646.66	20,846.80	14,833.58	1,498.26	1,758.23	1,236.72	650.30	1,533.15	72.83
Andorra	24,205.69	28,768.52	20,555.56	1,514.46	1,780.11	1,287.29	905.82	2,075.16	137.89
Angola	15,681.85	18,994.45	12,964.97	1,424.76	1,667.20	1,169.12	579.36	1,316.67	81.53
Antigua and Barbuda	18,006.58	21,475.41	15,213.58	1,288.91	1,510.60	1,095.68	675.46	1,574.42	93.74
Argentina	15,355.48	18,160.44	12,904.14	1,216.79	1,423.24	1,007.82	577.20	1,298.95	105.62
Armenia	18,239.24	21,637.09	15,549.32	1,356.05	1,582.07	1,136.63	676.70	1,526.41	115.42
Australia	18,457.82	21,769.95	15,577.62	1,370.18	1,604.42	1,148.48	678.82	1,546.87	111.62
Austria	23,077.07	27,546.95	19,557.76	1,524.18	1,777.09	1,296.50	873.60	1,959.68	149.43
Azerbaijan	17,998.72	21,371.41	15,321.91	1,353.45	1,574.86	1,127.70	667.47	1,509.02	110.66
Bahamas	17,916.80	21,369.02	15,135.36	1,319.76	1,541.39	1,117.94	671.06	1,565.74	94.12
Bahrain	19,264.69	22,664.10	16,320.68	1,282.63	1,519.63	1,077.45	760.75	1,710.68	138.35
Bangladesh	19,204.77	22,738.28	16,029.68	1,554.08	1,821.05	1,299.40	693.88	1,635.02	71.30
Barbados	17,959.35	21,387.20	15,229.93	1,271.52	1,489.49	1,082.84	675.66	1,582.15	93.63
Belarus	17,439.04	20,573.59	14,891.75	1,203.51	1,414.15	1,015.48	703.26	1,480.00	179.75
Belgium	30,611.94	35,980.68	26,029.82	1,740.23	2,023.61	1,489.76	1,126.25	2,664.54	126.76
Belize	17,675.23	21,306.38	14,692.23	1,398.40	1,642.13	1,163.52	659.88	1,561.55	83.13
Benin	18,912.50	22,904.46	15,616.90	1,636.40	1,924.70	1,340.47	699.44	1,649.92	82.01
Bermuda	17,909.78	21,276.47	15,276.50	1,225.18	1,435.82	1,039.42	677.47	1,563.69	100.16
Bhutan	19,013.28	22,476.31	15,883.57	1,523.81	1,796.80	1,273.55	686.51	1,618.58	67.41
Bolivia (Plurinational State of)	13,812.63	16,619.08	11,491.09	1,077.64	1,263.48	907.22	517.49	1,157.38	83.25
Bosnia and Herzegovina	18,221.95	21,321.46	15,523.23	1,291.31	1,508.41	1,099.14	685.80	1,531.86	138.14
Botswana	16,404.47	19,564.13	13,628.67	1,333.74	1,572.57	1,113.71	604.40	1,366.11	94.50
Brazil	24,349.12	28,942.02	20,862.05	1,527.67	1,712.68	1,339.84	895.25	2,148.68	87.16
Brunei Darussalam	14,185.75	16,874.99	11,987.04	1,084.28	1,278.10	906.35	530.73	1,197.63	101.63
Bulgaria	18,242.75	21,290.68	15,538.34	1,273.89	1,487.87	1,088.51	690.16	1,525.83	142.38
Burkina Faso	18,959.22	22,948.19	15,670.01	1,640.32	1,931.59	1,345.04	701.08	1,649.01	85.25
Burundi	11,250.18	13,560.96	9,304.82	1,040.77	1,220.22	845.66	421.17	939.30	79.91
Cabo Verde	19,314.36	23,073.08	16,178.87	1,498.90	1,768.82	1,252.35	720.56	1,679.14	94.46
Cambodia	20,369.22	24,423.96	17,079.89	1,649.51	1,942.51	1,371.44	761.50	1,868.67	77.80
Cameroon	18,930.07	22,909.38	15,664.04	1,619.87	1,907.08	1,329.64	698.48	1,640.95	83.31
Canada	22,549.11	26,353.58	19,324.38	1,532.11	1,791.01	1,293.74	835.47	1,935.70	113.38

Back to Top

Central African Republic	15,774.53	19,026.92	13,069.82	1,398.96	1,644.64	1,148.20	579.47	1,324.10	83.55
Chad	18,647.63	22,685.09	15,320.93	1,677.56	1,991.81	1,366.88	686.63	1,625.02	79.36
Chile	16,101.00	19,137.47	13,556.98	1,205.09	1,409.87	1,009.64	601.71	1,332.75	108.90
China	15,971.43	18,609.62	13,811.85	1,183.17	1,348.38	1,030.68	600.13	1,373.26	86.56
Colombia	18,319.25	21,757.28	15,460.09	1,333.17	1,553.44	1,122.80	688.06	1,598.49	89.38
Comoros	11,591.09	13,865.45	9,652.82	991.69	1,157.37	817.19	442.31	975.58	88.78
Congo	16,023.15	19,207.88	13,326.82	1,362.98	1,596.47	1,128.62	592.81	1,349.77	88.76
Cook Islands	18,355.30	21,598.11	15,537.42	1,462.32	1,708.58	1,226.61	679.76	1,602.06	80.79
Costa Rica	18,143.94	21,623.07	15,365.68	1,330.39	1,565.64	1,119.86	681.17	1,586.01	92.58
Cote d'Ivoire	18,926.58	22,827.44	15,748.29	1,579.22	1,862.97	1,302.66	698.35	1,638.76	84.67
Croatia	18,129.64	21,305.22	15,376.32	1,287.47	1,501.75	1,089.80	683.31	1,530.55	142.30
Cuba	17,764.77	21,096.23	15,153.30	1,208.53	1,412.96	1,022.13	669.12	1,545.63	95.48
Cyprus	24,931.96	29,661.52	20,973.98	1,611.25	1,898.77	1,365.08	928.32	2,131.94	131.23
Czechia	18,247.13	21,326.29	15,551.04	1,282.54	1,499.62	1,095.17	687.65	1,503.42	140.71
Democratic People's Republic of Korea	15,376.83	18,183.87	13,071.51	1,190.26	1,391.76	999.94	575.18	1,332.94	76.11
Democratic Republic of the Congo	15,593.96	18,878.51	12,893.17	1,402.90	1,650.04	1,149.92	572.46	1,305.20	80.78
Denmark	21,140.24	24,964.89	17,961.97	1,530.92	1,780.41	1,291.69	791.08	1,817.29	119.71
Djibouti	11,663.22	13,932.51	9,714.20	975.33	1,142.30	811.09	445.07	984.77	92.27
Dominica	17,544.20	20,894.73	14,770.01	1,303.47	1,521.55	1,095.05	656.34	1,526.27	87.50
Dominican Republic	17,675.28	21,168.51	14,787.14	1,344.36	1,576.82	1,128.28	661.01	1,547.98	85.13
Ecuador	15,600.39	18,881.25	13,107.60	1,154.18	1,358.23	966.81	584.60	1,337.59	85.29
Egypt	21,242.34	24,655.69	18,336.26	1,579.43	1,812.71	1,336.36	831.65	1,936.20	125.90
El Salvador	18,298.28	21,864.44	15,352.74	1,386.54	1,638.68	1,159.84	683.72	1,592.59	85.47
Equatorial Guinea	15,319.73	18,653.56	12,555.89	1,384.54	1,636.66	1,129.20	563.64	1,288.67	78.45
Eritrea	11,336.46	13,645.03	9,386.17	1,017.65	1,197.49	827.15	428.08	950.28	83.11
Estonia	17,181.32	20,233.16	14,649.39	1,199.96	1,408.49	1,009.89	692.16	1,460.35	177.11
Eswatini	16,060.82	19,369.77	13,281.66	1,381.25	1,630.50	1,139.47	587.69	1,340.50	83.39
Ethiopia	10,148.33	11,879.46	8,647.46	971.58	1,117.16	818.83	375.58	852.76	65.95
Fiji	18,038.42	21,281.49	15,167.72	1,476.79	1,730.56	1,235.56	665.12	1,579.20	79.74
Finland	24,080.29	28,555.92	20,342.13	1,631.51	1,915.31	1,383.56	892.11	2,055.08	124.23
France	23,471.36	27,587.39	20,019.04	1,635.21	1,897.09	1,386.98	889.94	2,021.00	144.97
Gabon	16,228.80	19,464.54	13,477.67	1,361.39	1,600.41	1,127.59	599.35	1,358.87	91.66
Gambia	18,980.15	23,003.34	15,693.35	1,629.40	1,918.92	1,333.63	698.71	1,651.59	83.74
Georgia	18,050.02	21,466.41	15,396.67	1,325.78	1,542.20	1,112.36	669.72	1,505.57	114.81
Germany	27,046.83	31,880.96	23,198.20	1,621.03	1,889.87	1,373.25	989.26	2,314.55	130.10
Ghana	19,307.38	23,275.13	16,058.10	1,575.33	1,858.43	1,302.85	715.37	1,676.62	92.16
Greece	25,650.38	30,355.09	21,808.47	1,610.64	1,893.46	1,367.32	948.68	2,192.19	134.87

Back to Top

Greenland	21,981.99	25,748.16	18,728.78	1,544.10	1,791.15	1,298.10	805.24	1,881.43	106.19
Grenada	17,540.04	20,916.42	14,745.35	1,283.52	1,503.27	1,077.68	657.28	1,543.41	87.40
Guam	17,927.72	21,066.86	15,135.95	1,444.97	1,686.05	1,212.82	665.48	1,565.40	79.85
Guatemala	17,977.24	21,666.05	14,986.19	1,426.20	1,688.38	1,178.26	669.85	1,573.53	79.22
Guinea	19,021.31	23,037.12	15,718.15	1,652.61	1,942.72	1,353.33	702.51	1,670.70	84.33
Guinea-Bissau	19,110.27	23,111.13	15,828.33	1,626.18	1,912.31	1,341.89	704.21	1,664.44	84.84
Guyana	17,750.73	21,329.14	14,821.18	1,351.24	1,580.04	1,130.97	656.73	1,539.18	82.73
Haiti	17,855.03	21,548.76	14,846.39	1,424.53	1,666.43	1,184.93	659.04	1,547.37	82.67
Honduras	17,939.79	21,648.75	14,940.30	1,433.26	1,693.27	1,184.36	670.02	1,570.39	81.53
Hungary	18,319.78	21,436.76	15,600.14	1,284.92	1,495.49	1,094.73	692.01	1,530.79	140.86
Iceland	24,022.31	28,474.52	20,212.11	1,665.11	1,950.15	1,409.77	891.24	2,069.98	118.36
India	19,379.27	22,352.04	16,706.15	1,586.89	1,794.67	1,371.26	697.31	1,611.61	78.72
Indonesia	21,073.03	24,382.49	18,142.42	1,682.62	1,892.89	1,461.13	791.88	1,938.28	86.72
Iran (Islamic Republic of)	21,811.99	25,101.45	18,875.73	1,567.04	1,770.95	1,368.74	854.89	1,925.87	155.94
Iraq	20,229.68	24,167.39	16,929.19	1,568.36	1,836.17	1,302.15	785.71	1,818.33	119.80
Ireland	24,211.50	28,686.00	20,481.98	1,668.39	1,969.97	1,413.79	897.67	2,070.09	126.60
Israel	23,816.85	28,232.12	19,981.75	1,762.72	2,074.90	1,481.47	883.45	2,049.22	114.81
Italy	28,256.27	32,833.01	24,524.66	1,751.81	1,994.11	1,535.74	1,054.76	2,487.02	135.72
Jamaica	17,831.32	21,346.31	14,951.53	1,328.94	1,559.36	1,119.49	667.59	1,567.67	88.13
Japan	14,968.87	17,260.18	12,920.41	1,035.46	1,177.71	899.45	570.84	1,264.84	121.03
Jordan	19,860.64	23,628.94	16,691.94	1,531.99	1,784.57	1,270.88	778.19	1,792.82	123.31
Kazakhstan	17,948.25	21,216.21	15,301.60	1,378.92	1,603.50	1,152.23	662.36	1,499.05	108.20
Kenya	11,941.36	14,000.72	10,138.51	1,070.87	1,236.96	906.31	453.76	1,008.63	89.67
Kiribati	17,919.91	21,261.02	14,965.32	1,536.45	1,804.70	1,276.18	657.62	1,567.34	72.18
Kuwait	20,540.96	24,234.09	17,564.02	1,403.79	1,661.46	1,187.73	799.82	1,837.23	135.71
Kyrgyzstan	17,582.69	20,825.93	14,892.16	1,414.49	1,660.11	1,169.96	649.90	1,467.72	104.85
Lao People's Democratic Republic	20,238.44	24,316.58	16,918.46	1,658.56	1,952.10	1,372.26	756.79	1,847.63	75.35
Latvia	17,316.61	20,378.56	14,798.23	1,196.06	1,407.60	1,004.62	698.58	1,462.51	177.96
Lebanon	20,866.94	24,556.87	17,679.36	1,507.15	1,760.29	1,261.95	814.83	1,884.66	136.41
Lesotho	16,077.71	19,326.47	13,309.27	1,355.55	1,596.69	1,122.50	588.06	1,342.01	87.31
Liberia	18,972.32	22,871.38	15,745.57	1,591.93	1,871.95	1,313.44	693.60	1,623.48	84.81
Libya	20,532.49	24,138.48	17,357.06	1,462.93	1,704.81	1,234.14	801.55	1,823.40	133.37
Lithuania	15,802.91	18,464.01	13,554.73	1,128.13	1,312.55	947.86	629.40	1,326.79	156.29
Luxembourg	23,398.33	27,844.08	19,916.39	1,552.53	1,817.76	1,324.34	888.11	2,007.37	147.78
Madagascar	11,357.67	13,677.63	9,412.38	1,024.01	1,199.82	831.74	430.93	965.34	83.80
Malawi	11,197.72	13,558.45	9,238.88	1,051.51	1,235.17	847.98	420.75	939.78	77.56
Malaysia	18,586.00	22,220.70	15,624.47	1,523.97	1,795.83	1,271.81	701.45	1,717.99	81.01
Maldives	20,012.51	23,954.49	16,794.14	1,503.66	1,775.47	1,262.04	752.15	1,817.23	82.35
Mali	18,740.57	22,780.74	15,415.70	1,653.64	1,955.77	1,350.53	692.49	1,646.43	81.73
Malta	24,446.13	29,048.43	20,582.77	1,592.70	1,862.42	1,354.81	907.62	2,083.71	127.28
Marshall Islands	17,853.06	21,136.83	14,957.81	1,506.64	1,765.80	1,255.39	657.36	1,549.98	73.86

Back to Top

Mauritania	18,946.07	22,917.15	15,660.90	1,625.61	1,912.54	1,332.70	702.84	1,653.26	83.11
Mauritius	20,834.40	24,767.73	17,528.76	1,544.43	1,809.32	1,310.10	778.72	1,883.45	85.93
Mexico	18,249.25	21,282.94	15,627.41	1,397.46	1,574.68	1,203.73	687.45	1,596.02	95.03
Micronesia (Federated States of)	17,648.98	20,879.57	14,803.69	1,507.29	1,767.68	1,241.15	651.09	1,530.88	72.97
Monaco	24,243.90	28,733.00	20,596.26	1,546.37	1,811.99	1,312.83	904.15	2,086.05	133.50
Mongolia	18,004.20	21,292.91	15,340.16	1,373.35	1,595.95	1,151.72	665.38	1,507.28	110.81
Montenegro	18,033.43	21,125.32	15,378.04	1,317.57	1,537.50	1,117.21	678.56	1,506.70	132.37
Morocco	20,553.19	24,218.44	17,384.73	1,514.20	1,761.78	1,265.86	802.61	1,858.53	129.42
Mozambique	11,266.44	13,652.78	9,304.68	1,055.05	1,239.62	851.45	421.23	941.03	77.64
Myanmar	20,587.42	24,589.92	17,276.21	1,631.55	1,914.35	1,371.17	770.59	1,875.14	78.77
Namibia	16,140.84	19,395.78	13,379.78	1,365.05	1,607.00	1,129.55	596.64	1,358.10	89.63
Nauru	17,693.42	21,016.66	14,792.52	1,534.65	1,804.54	1,269.88	654.06	1,555.61	71.88
Nepal	20,093.41	23,922.40	16,794.62	1,633.37	1,907.56	1,350.44	727.06	1,703.31	75.43
Netherlands	23,260.54	27,428.14	19,821.63	1,596.35	1,857.47	1,343.32	856.88	1,984.68	103.93
New Zealand	19,085.85	22,227.10	16,532.23	1,447.52	1,640.97	1,249.60	703.63	1,571.33	114.67
Nicaragua	17,840.30	21,349.04	14,949.15	1,387.89	1,635.06	1,155.03	667.87	1,547.43	84.05
Niger	18,541.70	22,597.53	15,189.72	1,685.97	2,004.03	1,369.98	685.35	1,638.77	79.20
Nigeria	19,450.80	22,943.27	16,621.93	1,725.44	1,969.43	1,465.28	718.42	1,673.32	91.29
Niue	18,011.70	21,188.54	15,240.08	1,442.90	1,690.03	1,212.15	665.22	1,556.16	80.39
North Macedonia	18,110.52	21,169.64	15,428.86	1,293.20	1,513.07	1,099.35	683.41	1,527.68	137.62
Northern Mariana Islands	18,123.10	21,382.96	15,331.73	1,376.20	1,612.74	1,146.80	673.03	1,599.71	82.72
Norway	26,479.61	30,442.64	23,098.28	1,787.19	2,019.17	1,562.15	966.99	2,232.04	114.87
Oman	18,924.39	22,391.52	15,848.03	1,326.09	1,583.14	1,094.96	746.97	1,701.50	127.70
Pakistan	18,154.90	21,293.11	15,549.94	1,626.51	1,847.52	1,387.19	645.01	1,522.35	54.60
Palau	18,090.82	21,337.77	15,339.11	1,348.97	1,577.49	1,139.02	670.28	1,564.69	84.49
Palestine	20,095.12	24,098.15	16,727.37	1,614.91	1,882.18	1,334.52	778.72	1,832.35	116.19
Panama	17,722.28	21,146.39	14,928.95	1,341.40	1,577.84	1,125.80	664.70	1,550.17	86.80
Papua New Guinea	17,741.92	21,055.87	14,803.74	1,518.28	1,786.51	1,260.25	651.07	1,564.36	72.44
Paraguay	22,702.69	27,730.13	18,803.73	1,540.98	1,793.32	1,312.58	835.81	2,027.69	74.75
Peru	12,327.48	14,898.40	10,280.39	974.93	1,138.39	819.86	467.96	1,038.50	84.83
Philippines	20,644.06	24,000.76	17,735.98	1,729.99	1,947.39	1,484.56	773.01	1,896.92	81.82
Poland	19,152.96	22,023.22	16,596.98	1,369.87	1,561.13	1,195.41	725.93	1,624.73	150.25
Portugal	24,535.14	29,086.39	20,832.53	1,584.69	1,864.32	1,348.61	914.41	2,103.05	133.27
Puerto Rico	17,985.99	21,420.39	15,237.72	1,280.44	1,501.23	1,088.48	675.66	1,570.07	94.51
Qatar	17,973.61	21,308.75	15,014.92	1,183.00	1,424.56	970.98	712.35	1,585.92	126.15
Republic of Korea	15,675.43	18,349.72	13,450.76	1,044.66	1,220.15	879.36	587.43	1,313.39	110.98
Republic of Moldova	17,354.73	20,433.89	14,761.87	1,211.09	1,421.34	1,021.99	697.36	1,454.15	176.46
Romania	18,113.74	21,231.36	15,436.83	1,283.69	1,491.57	1,093.30	685.61	1,507.98	141.73

Back to Top

Russian Federation	19,188.65	22,286.17	16,667.24	1,317.85	1,510.15	1,154.59	782.73	1,646.14	216.75
Rwanda	11,472.02	13,818.08	9,505.36	1,027.79	1,204.22	842.03	432.71	958.34	83.46
Saint Kitts and Nevis	17,764.53	21,182.99	14,998.37	1,280.41	1,496.35	1,088.50	666.94	1,549.90	93.85
Saint Lucia	17,842.10	21,277.90	15,077.38	1,275.18	1,497.93	1,083.07	667.56	1,553.50	94.73
Saint Vincent and the Grenadines	17,632.14	21,033.90	14,868.68	1,303.78	1,527.95	1,100.88	659.75	1,530.37	89.41
Samoa	17,298.02	20,580.43	14,403.83	1,532.16	1,793.74	1,259.61	640.40	1,533.74	71.79
San Marino	24,601.75	29,125.58	20,826.54	1,635.42	1,915.46	1,387.92	915.39	2,108.95	127.27
Sao Tome and Principe	18,972.77	22,847.76	15,785.55	1,582.18	1,864.04	1,304.36	705.31	1,658.13	85.35
Saudi Arabia	19,518.69	23,062.81	16,469.02	1,371.59	1,616.18	1,160.90	768.33	1,736.54	135.07
Senegal	18,898.47	22,838.23	15,633.53	1,608.21	1,891.80	1,317.31	697.87	1,635.33	86.46
Serbia	18,021.77	21,115.09	15,369.39	1,312.67	1,530.65	1,113.63	679.00	1,513.34	135.74
Seychelles	20,484.83	24,326.22	17,236.46	1,524.28	1,782.02	1,292.49	769.30	1,850.85	84.48
Sierra Leone	19,027.17	22,986.73	15,755.54	1,610.26	1,894.85	1,323.60	701.55	1,651.36	85.50
Singapore	13,369.76	15,932.11	11,315.18	945.07	1,115.04	793.04	517.94	1,138.58	117.75
Slovakia	18,319.22	21,402.13	15,612.91	1,293.56	1,510.22	1,102.18	690.61	1,550.65	140.24
Slovenia	18,183.87	21,239.23	15,494.40	1,268.15	1,482.22	1,080.15	686.81	1,524.98	140.44
Solomon Islands	17,587.70	20,895.02	14,752.99	1,539.85	1,803.84	1,267.95	648.04	1,535.99	73.51
Somalia	11,089.98	13,423.99	9,167.61	1,034.16	1,215.62	837.09	416.70	934.03	78.68
South Africa	17,170.28	20,003.34	14,782.72	1,369.13	1,555.35	1,175.64	634.93	1,415.97	103.38
South Sudan	11,151.18	13,444.00	9,226.93	1,037.54	1,218.42	839.86	417.12	920.74	80.52
Spain	24,739.85	29,330.87	21,097.00	1,565.52	1,844.97	1,331.91	936.19	2,118.47	150.74
Sri Lanka	20,662.13	24,577.19	17,347.02	1,600.84	1,872.74	1,351.91	774.51	1,877.48	80.18
Sudan	20,264.09	24,280.25	16,851.73	1,640.05	1,912.77	1,353.63	788.12	1,840.77	119.91
Suriname	17,702.07	21,082.82	14,920.13	1,326.36	1,544.41	1,114.30	659.65	1,542.30	89.58
Sweden	24,170.73	28,072.56	20,811.81	1,698.64	1,920.73	1,484.39	889.15	2,066.96	112.30
Switzerland	21,913.56	26,177.86	18,641.37	1,495.22	1,745.12	1,269.83	819.76	1,853.81	129.13
Syrian Arab Republic	20,405.08	24,281.95	17,102.40	1,569.35	1,836.62	1,292.00	792.88	1,860.35	126.81
Taiwan (Province of China)	17,311.47	20,583.77	14,603.68	1,231.50	1,448.82	1,027.08	646.47	1,495.41	86.16
Tajikistan	17,367.45	20,588.36	14,663.24	1,431.18	1,684.15	1,183.06	640.16	1,460.17	100.55
Thailand	23,433.07	27,487.64	19,908.40	1,579.00	1,840.07	1,341.38	875.78	2,115.50	90.03
Timor-Leste	19,511.26	23,426.80	16,155.92	1,720.62	2,044.59	1,407.52	729.22	1,820.62	66.83
Togo	19,131.32	23,020.83	15,951.76	1,589.60	1,868.56	1,309.05	707.93	1,662.96	86.88
Tokelau	17,814.00	20,991.58	15,000.12	1,485.32	1,737.06	1,231.85	659.60	1,550.10	77.06
Tonga	17,650.68	20,898.65	14,803.18	1,530.60	1,794.90	1,259.53	653.08	1,544.77	71.38
Trinidad and Tobago	17,795.81	21,219.69	15,059.92	1,286.05	1,508.46	1,089.77	666.49	1,546.48	91.60
Tunisia	20,814.29	24,473.30	17,647.21	1,475.67	1,718.13	1,246.72	817.32	1,874.25	138.18

Back to Top

Turkey	20,012.38	23,418.91	17,054.37	1,421.42	1,659.75	1,204.21	792.26	1,811.43	143.82
Turkmenistan	17,417.58	20,554.66	14,767.47	1,376.61	1,610.40	1,144.70	645.77	1,464.75	106.16
Tuvalu	17,627.27	20,857.25	14,735.35	1,487.06	1,746.56	1,234.42	652.01	1,535.54	74.81
Uganda	11,147.10	13,542.72	9,186.30	1,054.12	1,242.13	849.06	419.51	942.42	76.08
Ukraine	18,325.38	21,197.10	15,964.94	1,261.61	1,441.08	1,106.40	739.26	1,579.31	192.68
United Arab Emirates	18,166.03	21,541.53	15,295.72	1,155.57	1,415.40	945.33	718.60	1,602.48	142.25
United Kingdom	23,837.92	27,673.35	20,671.69	1,669.09	1,888.68	1,461.82	879.81	1,998.35	129.75
United Republic of Tanzania	11,701.96	14,004.85	9,700.47	1,056.08	1,256.17	860.48	442.62	985.94	82.54
United States of America	22,921.82	26,549.45	19,926.48	1,687.98	1,904.21	1,466.09	836.25	1,912.55	110.88
United States Virgin Islands	17,940.53	21,353.03	15,235.40	1,293.19	1,509.43	1,098.46	674.59	1,575.54	95.60
Uruguay	15,707.24	18,452.99	13,283.21	1,214.14	1,426.75	1,020.40	589.12	1,327.73	108.45
Uzbekistan	17,616.67	20,844.87	14,929.64	1,409.37	1,652.11	1,169.88	649.68	1,468.86	106.22
Vanuatu	17,621.17	20,930.19	14,739.57	1,547.87	1,815.20	1,276.92	648.40	1,544.69	72.41
Venezuela (Bolivarian Republic of)	17,935.87	21,295.92	15,169.72	1,337.48	1,564.32	1,135.60	673.81	1,560.63	89.13
Viet Nam	20,851.21	24,771.18	17,508.58	1,570.16	1,842.09	1,325.91	786.46	1,904.37	85.19
Yemen	20,142.79	24,116.24	16,778.12	1,660.55	1,936.06	1,372.34	776.62	1,794.55	113.23
Zambia	12,253.40	14,881.04	10,161.25	1,083.66	1,281.20	889.62	466.73	1,030.55	94.88
Zimbabwe	15,940.93	19,209.75	13,199.17	1,401.98	1,649.74	1,153.27	589.57	1,340.35	86.21

Back to Top

Supplementary Table 6. Rates of prevalence, incidence, and YLD per 100,000 population of TTH in adolescents and young adults by country and territory in 2019									
**Location**	**Prevalence Rate**			**Incidence Rate**			**Years Lived with Disability Rate**		
	**Mean**	**Upper Limit**	**Lower Limit**	**Mean**	**Upper Limit**	**Lower Limit**	**Mean**	**Upper Limit**	**Lower Limit**
Afghanistan	28,134.83	33,622.05	23,104.93	9,794.39	11,388.18	8,301.90	72.86	216.23	23.18
Albania	37,675.14	43,983.32	31,735.09	12,448.86	14,394.50	10,779.28	91.21	283.45	27.30
Algeria	28,511.98	33,903.60	23,900.46	9,726.60	11,291.72	8,347.46	83.07	232.08	27.95
American Samoa	27,472.25	32,610.64	22,782.58	9,358.15	10,883.58	8,034.26	55.51	213.22	15.44
Andorra	42,021.04	48,611.62	35,441.40	12,916.56	15,116.68	11,121.52	97.26	329.63	27.26
Angola	29,041.75	34,453.09	23,981.05	10,107.46	11,786.98	8,576.84	60.63	235.63	17.19
Antigua and Barbuda	29,770.38	34,923.88	24,770.52	9,964.37	11,494.46	8,568.97	65.45	244.64	19.00
Argentina	33,089.43	38,845.40	27,744.57	11,377.33	13,200.00	9,711.43	74.80	243.91	21.23
Armenia	39,084.37	45,844.56	33,080.67	12,814.29	14,926.88	10,987.93	85.44	284.83	23.77
Australia	33,635.93	39,446.41	28,459.79	11,473.46	13,351.17	9,932.12	77.17	248.58	22.73
Austria	41,308.41	47,932.81	35,008.09	13,028.83	15,177.58	11,165.41	103.55	328.07	31.09
Azerbaijan	39,010.14	45,658.47	33,102.86	12,824.34	14,920.02	11,025.36	84.44	283.76	22.96
Bahamas	29,737.57	34,864.67	24,770.86	9,985.90	11,529.05	8,615.41	64.59	241.89	18.60
Bahrain	29,080.27	34,766.71	24,227.92	9,764.49	11,510.63	8,286.61	86.11	237.98	29.06
Bangladesh	31,944.08	37,694.38	26,724.57	10,901.18	12,672.90	9,316.27	59.07	242.47	14.48
Barbados	29,707.64	34,820.27	24,712.81	9,925.95	11,494.42	8,529.12	66.30	243.24	19.74
Belarus	40,049.88	46,255.68	33,836.36	12,896.23	14,987.83	11,094.71	114.54	334.13	37.52
Belgium	41,725.95	48,303.72	35,375.03	13,021.27	15,106.60	11,191.46	92.68	316.50	25.70
Belize	29,699.98	35,144.12	24,528.37	10,072.58	11,682.46	8,629.89	60.72	245.87	17.13
Benin	30,397.78	36,136.44	25,088.53	10,473.99	12,197.02	8,933.46	62.01	248.85	17.04
Bermuda	29,703.24	35,003.09	24,778.19	9,868.14	11,506.43	8,474.13	68.45	240.48	20.53
Bhutan	32,158.06	37,909.33	26,787.62	10,957.30	12,751.46	9,346.62	58.76	244.05	14.97
Bolivia (Plurinational State of)	25,470.66	30,548.20	21,013.43	8,755.94	10,184.02	7,481.31	56.63	229.94	17.08
Bosnia and Herzegovina	37,604.45	43,797.64	31,833.97	12,336.95	14,277.96	10,674.21	94.20	286.96	28.98
Botswana	30,089.98	35,582.34	25,207.68	10,283.10	12,011.88	8,738.11	65.89	240.31	19.37
Brazil	35,247.77	40,070.21	30,746.31	11,622.22	13,373.20	10,071.94	69.06	272.06	18.16
Brunei Darussalam	36,858.16	43,220.05	31,095.78	12,290.07	14,315.86	10,620.00	76.19	281.02	20.10
Bulgaria	37,533.65	43,820.70	31,568.30	12,303.15	14,305.22	10,612.36	95.56	289.41	29.61
Burkina Faso	30,382.96	36,076.47	25,018.29	10,457.40	12,158.47	8,914.41	62.03	254.21	17.10
Burundi	25,155.52	30,180.44	20,570.18	8,976.32	10,474.54	7,581.09	54.93	215.05	15.92
Cabo Verde	31,647.06	37,312.39	26,421.02	10,690.07	12,453.20	9,147.79	67.88	256.04	19.70
Cambodia	30,960.94	36,501.51	25,756.24	10,263.10	11,917.46	8,716.03	59.57	251.08	15.47
Cameroon	30,571.70	36,323.00	25,180.69	10,510.34	12,221.73	8,967.60	62.46	245.91	17.02
Canada	41,940.37	48,380.36	35,411.46	13,089.91	15,146.29	11,189.43	87.24	294.08	23.33

Back to Top

Central African Republic	29,157.54	34,648.04	24,158.73	10,107.05	11,786.51	8,596.11	60.92	243.82	17.43
Chad	29,926.57	35,818.66	24,492.07	10,375.03	12,105.10	8,803.27	59.69	253.14	16.32
Chile	33,149.06	38,918.98	27,804.24	11,351.73	13,216.47	9,795.42	76.30	241.50	21.83
China	22,270.26	25,819.29	19,085.63	7,687.50	8,972.07	6,583.36	56.28	192.10	18.10
Colombia	29,802.59	35,101.55	24,711.26	10,022.64	11,560.82	8,637.73	63.77	250.58	18.00
Comoros	25,502.72	30,309.55	21,050.98	8,988.40	10,480.50	7,664.30	59.12	214.90	17.94
Congo	29,560.17	35,015.87	24,795.07	10,164.06	11,828.10	8,647.24	64.00	236.60	18.49
Cook Islands	27,648.20	32,535.44	22,924.24	9,343.48	10,890.08	8,039.88	58.70	217.67	16.51
Costa Rica	29,874.23	35,225.68	24,826.28	10,036.98	11,588.13	8,647.85	64.28	249.53	18.73
Cote d'Ivoire	30,890.20	36,578.49	25,629.39	10,564.07	12,290.01	8,989.33	63.55	251.94	17.54
Croatia	37,566.01	43,598.83	31,721.23	12,377.19	14,387.79	10,732.26	94.23	286.43	29.06
Cuba	29,625.79	34,660.97	24,509.15	9,872.22	11,552.85	8,477.86	67.23	248.50	20.23
Cyprus	42,505.08	49,201.01	35,806.23	13,172.77	15,350.37	11,268.36	95.80	321.63	26.56
Czechia	37,492.72	43,908.52	31,450.19	12,291.11	14,254.07	10,597.73	95.22	290.09	29.58
Democratic People's Republic of Korea	21,610.34	25,634.74	17,941.84	7,518.47	8,735.43	6,428.73	52.32	188.79	16.45
Democratic Republic of the Congo	29,125.41	34,656.34	24,098.41	10,124.69	11,834.17	8,588.02	60.18	240.45	16.78
Denmark	43,296.80	49,851.16	36,535.13	13,022.39	15,181.96	11,185.76	91.47	315.86	24.51
Djibouti	25,723.82	30,464.11	21,245.49	9,058.09	10,565.14	7,714.14	59.91	212.33	18.58
Dominica	29,676.80	34,824.95	24,671.57	9,986.19	11,589.79	8,620.51	63.23	244.38	18.27
Dominican Republic	29,798.45	35,182.31	24,645.82	10,054.82	11,576.84	8,641.91	62.28	249.05	17.71
Ecuador	23,596.76	28,033.56	19,713.62	8,461.29	9,876.01	7,233.95	55.22	222.25	17.27
Egypt	30,973.25	36,054.64	25,925.38	10,062.25	11,618.01	8,627.54	81.85	245.30	26.19
El Salvador	29,772.68	35,233.88	24,561.20	10,053.17	11,658.53	8,610.76	62.30	247.54	17.18
Equatorial Guinea	29,093.07	34,938.08	24,010.42	10,148.75	11,925.10	8,563.60	59.43	227.24	16.55
Eritrea	25,292.72	30,149.60	20,743.09	8,986.37	10,460.89	7,609.23	56.22	211.42	16.82
Estonia	39,948.41	45,990.23	33,671.72	12,888.31	14,978.80	11,088.06	113.21	333.22	36.41
Eswatini	29,567.64	35,230.07	24,697.43	10,209.90	11,934.96	8,662.74	62.44	230.53	17.85
Ethiopia	20,196.26	24,190.02	16,684.47	7,850.87	9,172.03	6,684.07	44.25	176.57	13.13
Fiji	27,827.80	33,149.11	23,012.88	9,456.31	11,005.22	8,096.65	57.33	217.54	15.47
Finland	41,760.42	48,199.44	35,369.10	13,043.83	15,096.89	11,229.10	91.96	312.84	24.85
France	40,132.78	46,655.30	33,968.58	12,980.56	14,998.91	11,225.52	98.90	310.84	29.11
Gabon	29,639.34	35,001.20	24,778.99	10,177.84	11,929.04	8,679.83	64.46	238.73	19.14
Gambia	30,503.12	36,263.00	25,088.85	10,499.66	12,221.21	8,953.03	61.97	250.05	17.43
Georgia	38,748.54	45,080.35	32,960.95	12,688.19	14,760.58	10,906.11	85.44	285.27	23.57
Germany	42,960.28	49,460.92	36,741.19	13,072.47	15,218.92	11,231.65	94.96	308.59	26.69
Ghana	31,027.90	36,784.76	25,704.03	10,581.21	12,310.27	9,038.22	65.39	244.23	18.20
Greece	41,929.48	48,464.57	35,449.05	12,967.50	15,098.79	11,175.03	96.11	322.61	27.26

Back to Top

Greenland	41,735.73	47,981.23	35,303.02	13,110.94	15,148.09	11,248.94	84.29	289.10	21.42
Grenada	29,695.76	34,842.95	24,514.07	9,979.81	11,563.35	8,595.82	63.42	246.11	18.32
Guam	27,575.07	32,625.06	22,776.48	9,346.28	10,885.93	8,042.47	57.90	217.37	16.34
Guatemala	29,775.90	35,452.62	24,518.52	10,116.96	11,723.11	8,624.31	59.91	252.99	16.26
Guinea	30,375.23	36,160.61	25,007.63	10,468.62	12,182.18	8,931.55	61.86	250.68	17.10
Guinea-Bissau	30,671.32	36,442.67	25,215.69	10,545.48	12,274.21	8,978.21	62.63	251.91	17.40
Guyana	29,751.95	35,165.83	24,517.14	10,043.34	11,618.48	8,600.72	61.50	247.96	17.15
Haiti	29,780.38	35,298.55	24,507.23	10,108.15	11,695.70	8,628.47	60.06	252.56	16.27
Honduras	29,713.06	35,290.50	24,423.52	10,099.99	11,726.64	8,630.49	59.77	250.37	16.40
Hungary	37,541.71	43,788.48	31,533.47	12,285.30	14,239.82	10,627.50	95.35	294.43	29.46
Iceland	41,629.74	48,125.58	35,152.54	13,071.01	15,100.89	11,234.78	90.61	313.65	23.99
India	32,365.01	37,164.64	28,034.12	11,357.62	13,007.14	9,850.96	61.91	246.46	16.24
Indonesia	32,128.28	36,638.94	27,894.22	10,889.64	12,572.62	9,444.61	65.52	253.15	17.90
Iran (Islamic Republic of)	35,923.30	40,812.53	31,022.12	11,141.24	12,937.05	9,551.87	98.66	286.44	31.18
Iraq	28,362.01	33,814.75	23,519.23	9,766.25	11,326.16	8,332.27	78.03	225.62	25.62
Ireland	41,617.62	48,146.47	35,339.89	13,020.57	15,068.03	11,212.49	92.84	320.59	25.95
Israel	41,120.74	47,607.16	34,826.08	13,059.93	15,030.38	11,248.51	87.89	305.36	23.54
Italy	45,188.01	50,912.07	39,589.18	14,285.97	16,606.33	12,331.65	100.32	362.71	27.06
Jamaica	29,826.31	35,162.12	24,607.82	10,045.00	11,614.21	8,635.18	63.17	243.08	17.79
Japan	37,487.81	42,771.70	32,692.51	12,721.12	14,783.52	11,056.91	86.00	273.66	25.00
Jordan	28,369.96	33,716.69	23,546.75	9,761.27	11,278.95	8,347.06	78.46	227.18	25.89
Kazakhstan	38,820.78	45,291.48	32,899.94	12,805.85	14,880.61	10,988.23	83.45	277.82	22.57
Kenya	26,632.32	30,789.47	22,868.90	9,487.05	10,923.45	8,114.87	60.58	222.14	18.15
Kiribati	27,858.46	33,330.76	23,013.08	9,532.05	11,074.76	8,108.52	55.28	224.49	15.20
Kuwait	28,736.34	34,212.77	23,958.44	9,698.22	11,323.42	8,311.43	83.33	231.79	27.72
Kyrgyzstan	38,786.24	45,348.90	32,926.80	12,893.54	14,932.61	11,081.00	80.62	276.93	21.47
Lao People's Democratic Republic	30,906.53	36,517.20	25,625.01	10,252.05	11,904.93	8,696.60	59.06	247.51	15.12
Latvia	39,851.74	45,987.81	33,650.03	12,845.29	14,931.54	11,054.58	114.40	336.86	37.88
Lebanon	28,485.66	33,822.10	23,774.55	9,716.67	11,305.65	8,316.65	83.59	231.86	28.26
Lesotho	29,754.23	35,204.79	24,840.76	10,231.57	11,972.08	8,679.46	63.29	235.90	18.15
Liberia	30,687.95	36,272.82	25,354.26	10,502.20	12,222.07	8,955.97	63.03	247.84	17.54
Libya	28,545.24	33,826.23	23,863.05	9,711.34	11,296.67	8,341.86	83.44	235.92	27.75
Lithuania	42,487.20	48,309.14	36,656.08	12,921.57	15,139.30	11,111.02	106.75	338.85	32.84
Luxembourg	41,440.95	47,895.92	35,069.41	13,087.23	15,223.41	11,204.31	101.82	320.64	30.46
Madagascar	25,234.28	30,061.82	20,730.95	8,970.31	10,449.52	7,608.87	56.55	215.71	16.84
Malawi	25,024.93	30,081.30	20,435.67	8,950.12	10,438.97	7,544.76	54.40	211.31	15.54
Malaysia	31,000.48	36,527.19	25,776.86	10,218.69	11,860.68	8,705.28	61.02	240.08	15.72
Maldives	31,281.51	37,297.22	25,951.82	10,274.32	12,119.83	8,581.61	62.66	249.99	16.08
Mali	30,085.92	35,930.91	24,663.40	10,404.01	12,136.22	8,846.28	60.62	252.78	16.55
Malta	42,212.27	48,733.49	35,579.95	13,079.40	15,188.33	11,228.03	94.24	318.05	26.02
Marshall Islands	27,837.56	33,309.64	23,023.67	9,500.99	11,043.68	8,085.38	56.05	219.12	15.03

Back to Top

Mauritania	30,427.19	36,079.96	25,092.20	10,458.09	12,158.50	8,924.35	62.82	243.44	17.31
Mauritius	30,929.22	36,193.52	25,864.80	10,071.12	11,708.05	8,641.17	63.09	241.87	16.56
Mexico	31,161.45	35,456.83	26,759.26	10,694.76	12,242.93	9,291.76	67.52	257.35	19.48
Micronesia (Federated States of)	27,620.67	32,876.48	22,861.09	9,432.25	10,957.51	8,059.74	55.28	219.66	15.10
Monaco	41,726.91	48,366.93	35,347.42	12,915.34	15,056.74	11,127.71	96.13	320.99	27.24
Mongolia	38,934.61	45,503.79	32,989.54	12,820.71	14,900.04	11,018.89	83.62	281.96	22.77
Montenegro	37,612.40	43,850.98	31,884.38	12,403.70	14,360.41	10,727.48	92.55	287.57	28.06
Morocco	28,422.09	33,715.61	23,776.21	9,713.15	11,247.73	8,323.70	81.56	228.08	27.58
Mozambique	25,008.05	30,099.73	20,401.33	8,943.45	10,426.92	7,556.74	54.51	211.14	15.80
Myanmar	30,906.31	36,339.42	25,778.31	10,191.27	11,833.45	8,712.39	60.66	244.34	15.67
Namibia	29,631.71	35,062.53	24,808.40	10,196.20	11,939.14	8,672.63	63.97	234.50	18.51
Nauru	27,788.40	33,315.83	22,922.00	9,523.29	11,066.73	8,095.05	55.06	217.50	14.69
Nepal	32,184.35	37,915.93	26,775.50	10,938.20	12,603.25	9,381.26	60.80	247.67	15.77
Netherlands	44,703.10	51,766.89	37,666.44	13,021.89	15,354.51	11,073.99	89.36	346.07	21.88
New Zealand	34,902.92	39,860.57	30,586.46	12,029.78	13,799.89	10,435.90	81.04	259.28	24.10
Nicaragua	29,772.63	35,239.51	24,599.11	10,085.05	11,687.80	8,614.26	61.22	253.30	16.73
Niger	29,806.08	35,682.40	24,397.05	10,358.43	12,108.98	8,778.92	59.04	252.06	16.16
Nigeria	31,514.99	36,305.66	27,137.52	11,140.54	12,860.47	9,575.77	65.79	256.49	18.74
Niue	27,579.40	32,604.87	22,907.79	9,322.31	10,858.97	8,008.63	58.10	214.49	16.29
North Macedonia	37,748.08	44,035.72	31,900.11	12,407.34	14,378.37	10,712.67	93.78	286.62	28.47
Northern Mariana Islands	27,183.59	32,218.27	22,561.13	9,060.12	10,635.07	7,760.41	59.33	213.99	17.05
Norway	45,354.64	50,900.03	39,776.00	14,357.25	16,637.86	12,176.00	93.61	342.44	24.02
Oman	29,052.34	35,050.71	23,806.00	9,898.88	11,686.18	8,274.22	82.13	230.81	26.61
Pakistan	33,315.50	38,174.46	28,579.64	11,649.15	13,355.45	10,038.64	53.58	247.51	11.08
Palau	27,561.66	32,510.21	22,782.76	9,222.19	10,817.16	7,894.78	60.52	218.05	17.61
Palestine	28,267.57	33,797.88	23,448.59	9,779.35	11,336.25	8,342.41	76.23	221.36	24.78
Panama	29,703.12	34,872.42	24,660.01	10,023.59	11,587.23	8,623.09	62.64	247.71	17.58
Papua New Guinea	27,798.55	33,217.74	22,900.60	9,508.55	11,045.50	8,080.71	54.86	227.68	14.81
Paraguay	33,176.53	39,202.70	27,674.25	10,823.57	12,562.90	9,270.81	61.64	248.68	15.26
Peru	27,279.60	32,103.08	22,613.17	8,962.44	10,438.96	7,632.07	60.29	235.60	17.88
Philippines	32,056.49	36,814.59	27,713.53	10,964.12	12,711.53	9,478.31	63.03	251.78	16.89
Poland	39,540.79	44,809.45	34,536.75	13,217.29	15,260.99	11,380.18	101.03	305.46	31.27
Portugal	41,927.34	48,510.41	35,446.39	12,960.26	15,071.64	11,179.45	95.97	318.71	26.72
Puerto Rico	29,706.73	34,798.42	24,728.31	9,928.83	11,461.64	8,531.22	66.00	239.59	19.14
Qatar	29,143.43	35,443.74	23,717.17	9,882.79	11,748.00	8,229.27	82.49	232.32	26.88
Republic of Korea	40,776.31	46,293.07	35,465.13	12,338.16	14,340.44	10,550.50	85.02	293.89	22.15
Republic of Moldova	40,226.31	46,537.82	34,005.22	12,955.01	15,036.94	11,123.30	113.01	329.77	36.95
Romania	37,403.93	43,574.61	31,447.65	12,286.69	14,277.55	10,627.49	94.74	289.06	29.33

Back to Top

Russian Federation	41,018.41	46,541.32	35,792.71	13,652.96	15,854.93	11,777.12	129.18	367.66	44.34
Rwanda	25,309.56	30,143.39	20,759.16	8,996.99	10,491.58	7,580.07	56.68	208.96	17.04
Saint Kitts and Nevis	29,765.95	34,940.02	24,768.52	9,967.99	11,531.63	8,578.91	65.22	245.96	18.76
Saint Lucia	29,766.19	34,928.64	24,751.98	9,958.60	11,503.46	8,558.10	65.06	246.06	18.79
Saint Vincent and the Grenadines	29,690.81	34,806.95	24,734.16	9,973.40	11,539.84	8,594.87	63.55	247.10	18.41
Samoa	27,474.20	32,867.45	22,727.88	9,437.74	10,971.54	8,056.21	54.01	221.27	14.36
San Marino	41,673.04	48,255.72	35,273.04	12,992.06	15,096.09	11,172.18	94.42	319.49	26.49
Sao Tome and Principe	30,776.31	36,361.05	25,439.09	10,514.88	12,226.08	8,979.19	64.24	248.04	17.97
Saudi Arabia	26,428.09	31,300.59	21,946.90	9,435.32	10,963.32	8,052.61	81.50	220.73	28.24
Senegal	30,498.29	36,127.08	25,146.84	10,478.58	12,190.04	8,940.70	62.54	249.13	17.28
Serbia	37,604.59	43,876.55	31,832.42	12,395.09	14,345.69	10,715.57	92.82	285.92	28.29
Seychelles	30,857.71	36,019.78	25,729.52	10,073.95	11,718.64	8,622.58	63.26	244.37	17.50
Sierra Leone	30,651.11	36,348.84	25,243.61	10,527.53	12,267.08	8,968.73	62.66	252.88	17.31
Singapore	36,736.30	42,987.04	30,625.55	12,215.43	14,341.72	10,411.91	81.64	301.21	23.59
Slovakia	37,749.66	44,083.91	31,774.87	12,371.34	14,353.31	10,659.45	94.90	290.86	28.86
Slovenia	37,554.09	43,860.63	31,681.39	12,301.37	14,290.97	10,612.77	95.51	292.91	29.06
Solomon Islands	27,702.59	33,134.04	22,930.60	9,500.67	11,018.57	8,076.33	54.36	227.05	14.52
Somalia	25,104.05	30,268.94	20,502.34	8,973.61	10,452.32	7,556.30	54.05	218.56	15.72
South Africa	31,530.40	36,364.30	27,111.50	10,919.16	12,631.61	9,412.51	71.59	247.62	21.58
South Sudan	24,913.79	29,909.43	20,317.09	8,891.27	10,427.23	7,558.45	54.66	211.15	16.27
Spain	40,609.25	47,405.69	34,215.95	12,953.35	15,142.15	11,018.83	101.79	326.82	29.55
Sri Lanka	30,889.52	36,173.56	25,892.35	10,144.23	11,741.64	8,689.39	61.96	237.03	16.44
Sudan	28,226.87	33,766.53	23,401.17	9,776.27	11,330.47	8,316.14	76.22	218.64	25.00
Suriname	29,658.37	34,671.65	24,720.40	9,986.60	11,585.01	8,616.54	63.13	246.10	17.73
Sweden	43,270.75	48,937.62	37,808.08	14,098.79	16,383.58	12,162.38	87.40	301.43	22.75
Switzerland	38,696.21	44,842.28	32,559.44	12,902.14	14,966.57	10,986.64	91.07	295.04	26.26
Syrian Arab Republic	28,114.42	33,360.00	23,474.40	9,678.61	11,246.18	8,245.12	78.53	224.82	26.39
Taiwan (Province of China)	21,825.05	26,135.32	18,128.14	7,529.82	8,824.92	6,415.70	55.07	185.28	17.27
Tajikistan	38,779.04	45,537.47	32,789.38	12,940.89	14,973.34	11,106.43	79.02	270.66	20.32
Thailand	30,914.44	36,294.90	25,876.21	10,024.96	11,694.76	8,589.64	65.11	244.69	17.92
Timor-Leste	30,496.11	36,552.79	24,854.07	10,210.39	11,943.54	8,656.68	55.90	247.91	13.80
Togo	30,792.39	36,405.46	25,447.73	10,518.69	12,239.39	8,965.30	64.27	248.38	18.09
Tokelau	27,614.52	32,831.06	22,983.54	9,404.07	10,947.71	8,064.54	56.64	216.10	15.47
Tonga	27,596.10	32,998.88	22,924.52	9,447.01	10,939.00	8,076.80	55.18	216.35	14.89
Trinidad and Tobago	29,860.72	35,190.34	24,751.89	9,998.44	11,545.50	8,636.03	65.36	242.34	18.82
Tunisia	28,498.84	33,746.92	23,937.24	9,682.32	11,260.30	8,358.28	84.67	232.53	28.16

Back to Top

Turkey	26,188.73	30,988.98	22,098.80	9,357.91	10,884.12	8,097.36	83.74	219.46	29.35
Turkmenistan	38,756.04	45,332.18	32,923.52	12,836.20	14,846.46	11,020.15	81.41	277.07	21.36
Tuvalu	27,663.22	32,945.84	22,849.48	9,439.39	11,014.76	8,043.68	55.72	218.14	15.14
Uganda	24,945.55	30,081.24	20,318.06	8,934.68	10,452.50	7,544.47	54.17	209.20	15.64
Ukraine	41,778.31	47,347.82	36,510.18	13,778.70	16,006.80	11,873.01	121.09	350.25	39.33
United Arab Emirates	29,484.01	35,612.23	23,742.54	9,886.42	11,874.34	8,123.08	87.49	242.58	28.11
United Kingdom	42,750.50	48,301.64	37,336.89	13,985.31	16,213.01	12,151.50	95.21	316.56	26.58
United Republic of Tanzania	25,158.91	30,135.37	20,605.56	8,957.68	10,446.60	7,603.21	56.14	209.90	16.83
United States of America	43,153.52	48,854.71	37,609.79	14,200.43	16,294.37	12,253.94	89.13	301.00	23.87
United States Virgin Islands	29,595.64	34,653.11	24,630.72	9,905.12	11,481.86	8,520.04	66.06	240.19	19.54
Uruguay	33,056.22	38,768.28	27,798.69	11,330.76	13,156.54	9,699.93	75.66	246.25	21.84
Uzbekistan	38,823.84	45,462.63	32,979.97	12,884.36	14,941.05	11,066.12	80.84	275.16	21.33
Vanuatu	27,728.18	33,199.06	22,910.72	9,524.88	11,068.98	8,112.75	54.31	221.67	14.55
Venezuela (Bolivarian Republic of)	29,744.10	34,826.01	24,728.14	10,001.13	11,560.68	8,615.90	63.83	251.76	18.39
Viet Nam	31,060.77	36,447.37	25,913.81	10,175.08	11,850.30	8,711.43	63.17	243.07	16.74
Yemen	28,246.44	33,877.80	23,332.81	9,806.05	11,334.46	8,320.19	74.95	215.16	24.01
Zambia	26,621.15	31,954.30	21,966.73	9,170.56	10,698.52	7,776.65	62.95	220.56	19.68
Zimbabwe	29,332.75	34,880.26	24,353.20	10,155.72	11,872.45	8,632.59	62.04	237.38	17.43

Supplementary Table 7. Global absolute numbers and rates of prevalence, incidence, and YLD of overall headache disorders in adolescents and young adults by sex and age group in 2019							
**Headache Disorder**		**Prevalence**					
		**Number (95% UI)**			**Rate (95% UI)**		
**Gender**	**Age**	**Mean**	**Upper**	**Lower**	**Mean**	**Upper**	**Lower**
Male	10-14 years	101,369,810	128,690,465	78,892,924	30,594.43	38,840.08	23,810.68
Male	15-19 years	114,043,972	143,060,177	89,367,914	35,887.47	45,018.32	28,122.39
Male	20-24 years	115,121,636	141,599,513	91,194,412	37,823.15	46,522.44	29,961.87
Male	25-29 years	120,362,694	146,422,208	97,875,544	39,492.05	48,042.40	32,113.82
Male	30-34 years	121,302,739	146,237,122	96,941,306	40,010.85	48,235.28	31,975.40
Male	35-39 years	114,486,617	137,136,141	94,359,649	42,007.24	50,317.76	34,622.28
Male	40-44 years	99,040,624	119,086,857	80,388,172	39,816.19	47,875.16	32,317.56
Male	45-49 years	84,485,830	105,650,321	68,446,671	35,442.82	44,321.57	28,714.20
Male	50-54 years	76,124,505	93,319,259	61,495,585	34,985.94	42,888.46	28,262.66
Female	10-14 years	110,389,943	135,237,152	88,604,270	35,512.00	43,505.25	28,503.64
Female	15-19 years	127,891,071	154,673,329	103,784,062	42,381.88	51,257.26	34,393.04
Female	20-24 years	133,282,788	158,208,672	110,494,804	45,062.03	53,489.31	37,357.57
Female	25-29 years	141,267,939	165,504,251	118,938,173	46,980.78	55,040.93	39,554.68
Female	30-34 years	141,794,854	165,261,477	117,745,440	47,493.26	55,353.25	39,438.07
Female	35-39 years	132,920,684	153,159,306	113,817,446	49,516.73	57,056.19	42,400.23
Female	40-44 years	117,776,867	136,325,506	99,929,365	48,131.41	55,711.61	40,837.74
Female	45-49 years	103,670,805	123,322,871	87,499,998	44,034.88	52,382.23	37,166.22
Female	50-54 years	94,646,504	111,502,819	80,055,761	43,172.07	50,860.91	36,516.65
**Headache Disorder**		**Incidence**					
		**Number (95% UI)**			**Rate (95% UI)**		
**Gender**	**Age**	**Mean**	**Upper**	**Lower**	**Mean**	**Upper**	**Lower**
Male	10-14 years	40,626,965	54,447,036	29,348,673	12,261.63	16,432.66	8,857.73
Male	15-19 years	35,426,279	48,186,400	23,913,628	11,147.98	15,163.35	7,525.16
Male	20-24 years	35,887,137	47,118,617	24,428,309	11,790.70	15,480.79	8,025.91

Back to Top

Male	25-29 years	36,362,009	50,503,811	24,968,524	11,930.69	16,570.74	8,192.39
Male	30-34 years	37,817,455	51,548,922	24,459,840	12,473.82	17,003.05	8,067.90
Male	35-39 years	34,895,258	46,808,105	23,362,425	12,803.71	17,174.75	8,572.10
Male	40-44 years	25,600,052	35,774,072	17,003,031	10,291.70	14,381.85	6,835.54
Male	45-49 years	23,472,366	33,907,828	15,427,066	9846.94	14224.74	6471.84
Male	50-54 years	19,889,153	27,913,328	13,491,950	9140.83	12828.64	6200.74
Female	10-14 years	43,181,290	56,877,828	31,966,164	13,891.25	18,297.37	10,283.39
Female	15-19 years	37,790,330	51,088,450	26,028,226	12,523.35	16,930.22	8,625.50
Female	20-24 years	38,711,386	50,574,601	27,051,897	13,088.06	17,098.94	9,146.07
Female	25-29 years	39,174,953	53,764,437	27,620,493	13,028.22	17,880.17	9,185.61
Female	30-34 years	40,054,683	53,968,735	26,389,095	13,416.06	18,076.48	8,838.86
Female	35-39 years	37,246,195	49,553,105	25,469,693	13,875.27	18,459.94	9,488.18
Female	40-44 years	27,697,422	38,342,587	18,932,387	11,319.00	15,669.31	7,737.02
Female	45-49 years	25,363,851	35,759,903	16,771,610	10773.47	15189.26	7123.86
Female	50-54 years	22,033,020	30,085,986	15,353,159	10050.14	13723.43	7003.19
**Headache Disorder**		**Years Lived with Disability**					
		**Number (95% UI)**			**Rate (95% UI)**		
**Gender**	**Age**	**Mean**	**Upper**	**Lower**	**Mean**	**Upper**	**Lower**
Male	10-14 years	1,157,157	2,803,641	65,948	349.24	846.17	19.9
Male	15-19 years	1,652,949	3,872,517	198,048	520.15	1,218.61	62.32
Male	20-24 years	1,766,918	4,056,036	272,252	580.52	1,332.61	89.45
Male	25-29 years	1,875,180	4,186,804	332,592	615.26	1,373.73	109.13
Male	30-34 years	1,948,269	4,289,320	411,875	642.62	1,414.80	135.85
Male	35-39 years	1,808,901	3,850,593	394,526	663.72	1,412.85	144.76
Male	40-44 years	1,676,233	3,494,439	437,918	673.88	1,404.83	176.05
Male	45-49 years	1,513,418	3,178,669	438,375	634.9	1,333.49	183.9
Male	50-54 years	1,277,954	2,625,118	379,370	587.33	1,206.47	174.35
Female	10-14 years	1,752,682	4,313,883	87,202	563.83	1,387.76	28.05
Female	15-19 years	2,533,899	6,219,507	284,235	839.71	2,061.09	94.19
Female	20-24 years	2,791,735	6,536,356	400,904	943.87	2,209.90	135.54

Back to Top

Female	25-29 years	3,023,977	6,775,018	467,646	1,005.67	2,253.13	155.52
Female	30-34 years	3,155,602	7,148,305	570,160	1,056.95	2,394.28	190.97
Female	35-39 years	2,964,591	6,533,117	548,081	1,104.39	2,433.77	204.18
Female	40-44 years	2,776,127	6,169,090	597,595	1,134.51	2,521.10	244.22
Female	45-49 years	2,520,833	5,676,978	597,845	1,070.74	2,411.33	253.94
Female	50-54 years	2,159,567	4,648,992	525,643	985.07	2,120.59	239.77

Back to Top

Supplementary Table 8. Global absolute numbers and rates of prevalence, incidence, and YLD of migraine in adolescents and young adults by sex and age group in 2019

Migraine		Prevalence					
		Number (95% UI)			Rate (95% UI)		
Gender	Age	Mean	Upper	Lower	Mean	Upper	Lower
Male	10-14 years	29,552,171	42,034,537	20,512,390	8,919.14	12,686.45	6,190.85
Male	15-19 years	40,774,776	54,070,687	30,429,831	12,831.05	17,015.02	9,575.69
Male	20-24 years	43,127,484	54,659,815	33,540,628	14,169.51	17,958.45	11,019.75
Male	25-29 years	45,494,909	56,510,731	36,123,876	14,927.28	18,541.66	11,852.56
Male	30-34 years	46,588,831	58,556,130	37,153,573	15,367.00	19,314.32	12,254.84
Male	35-39 years	42,322,210	52,759,125	34,379,124	15,528.79	19,358.29	12,614.33
Male	40-44 years	38,762,615	48,136,716	31,689,356	15,583.30	19,351.87	12,739.72
Male	45-49 years	34,298,703	42,192,447	27,448,427	14,388.72	17,700.24	11,514.94
Male	50-54 years	28,296,463	34,999,020	22,789,803	13,004.73	16,085.15	10,473.93
Female	10-14 years	45,580,022	63,697,319	32,209,372	14,662.91	20,491.17	10,361.62
Female	15-19 years	63,983,587	83,207,742	48,333,750	21,203.55	27,574.25	16,017.34
Female	20-24 years	70,272,928	87,679,149	55,042,535	23,758.81	29,643.74	18,609.52
Female	25-29 years	76,491,179	94,744,403	61,114,234	25,438.29	31,508.68	20,324.46
Female	30-34 years	79,307,163	98,872,172	63,752,778	26,563.42	33,116.59	21,353.58
Female	35-39 years	73,398,779	89,941,409	59,965,688	27,343.13	33,505.73	22,338.92
Female	40-44 years	68,196,716	83,094,489	56,076,748	27,869.68	33,957.90	22,916.67
Female	45-49 years	61,056,679	74,408,490	49,331,513	25,934.24	31,605.51	20,953.89
Female	50-54 years	51,427,630	62,942,531	41,536,647	23,458.21	28,710.62	18,946.53
Migraine		Incidence					
		Number (95% UI)			Rate (95% UI)		
Gender	Age	Mean	Upper	Lower	Mean	Upper	Lower
Male	10-14 years	5,952,349	7,873,522	4,228,248	1,796.48	2,376.31	1,276.13
Male	15-19 years	3,869,900	5,498,555	2,508,985	1,217.78	1,730.29	789.53
Male	20-24 years	3,276,050	4,955,523	2,055,026	1,076.34	1,628.13	675.18

Back to Top

Male	25-29 years	3,362,349	4,927,470	2,041,209	1,103.22	1,616.75	669.74
Male	30-34 years	3,095,322	4,495,121	1,954,444	1,020.97	1,482.68	644.66
Male	35-39 years	2,780,935	3,971,836	1,784,311	1,020.38	1,457.34	654.7
Male	40-44 years	2,243,357	3,244,780	1,383,181	901.87	1,304.46	556.06
Male	45-49 years	1,559,877	2,228,171	973,691	654.39	934.74	408.48
Male	50-54 years	1,195,501	1,797,580	728,802	549.44	826.15	334.95
Female	10-14 years	9,075,220	11,846,507	6,447,126	2,919.46	3,810.97	2,074.01
Female	15-19 years	6,108,841	8,640,743	3,974,062	2,024.41	2,863.46	1,316.97
Female	20-24 years	5,370,654	8,048,719	3,380,950	1,815.78	2,721.22	1,143.08
Female	25-29 years	5,682,219	8,339,492	3,488,732	1,889.71	2,773.42	1,160.23
Female	30-34 years	5,326,838	7,678,825	3,431,335	1,784.19	2,571.97	1,149.30
Female	35-39 years	4,919,869	7,076,627	3,154,550	1,832.79	2,636.24	1,175.16
Female	40-44 years	3,951,931	5,695,307	2,431,410	1,615.02	2,327.48	993.63
Female	45-49 years	2,662,687	3,848,076	1,673,212	1,130.99	1,634.50	710.71
Female	50-54 years	2,045,449	3,100,356	1,204,941	933.01	1,414.20	549.62
**Migraine**		**Years Lived with Disability**					
		**Number (95% UI)**			**Rate (95% UI)**		
**Gender**	**Age**	**Mean**	**Upper**	**Lower**	**Mean**	**Upper**	**Lower**
Male	10-14 years	1,059,291	2,657,750	44,512	319.7	802.14	13.43
Male	15-19 years	1,509,054	3,701,475	137,829	474.87	1,164.78	43.37
Male	20-24 years	1,603,101	3,806,592	189,675	526.7	1,250.65	62.32
Male	25-29 years	1,688,340	3,875,100	227,188	553.96	1,271.45	74.54
Male	30-34 years	1,744,574	3,970,213	268,235	575.44	1,309.55	88.48
Male	35-39 years	1,603,176	3,644,011	276,821	588.23	1,337.05	101.57
Male	40-44 years	1,483,910	3,328,911	304,756	596.56	1,338.28	122.52
Male	45-49 years	1,332,223	2,922,375	307,803	558.88	1,225.97	129.13
Male	50-54 years	1,109,506	2,403,597	255,714	509.92	1,104.67	117.52
Female	10-14 years	1,647,372	4,205,217	61,167	529.95	1,352.80	19.68
Female	15-19 years	2,365,735	6,123,279	194,251	783.98	2,029.20	64.37
Female	20-24 years	2,591,951	6,140,300	266,125	876.32	2,075.99	89.97

Back to Top

Female	25-29 years	2,797,861	6,589,573	314,332	930.47	2,191.46	104.54
Female	30-34 years	2,911,174	6,751,035	368,034	975.08	2,261.22	123.27
Female	35-39 years	2,714,125	6,316,864	390,513	1,011.09	2,353.21	145.48
Female	40-44 years	2,537,068	5,804,052	418,275	1,036.81	2,371.92	170.93
Female	45-49 years	2,292,676	5,197,757	423,213	973.83	2,207.78	179.76
Female	50-54 years	1,943,747	4,386,975	361,405	886.62	2,001.08	164.85

Back to Top

Supplementary Table 9. Global absolute numbers and rates of prevalence, incidence, and YLD of TTH in adolescents and young adults by sex and age group in 2019							
TTH		Prevalence					
		Number (95% UI)			Rate (95% UI)		
**Gender**	**Age**	**Mean**	**Upper**	**Lower**	**Mean**	**Upper**	**Lower**
Male	10-14 years	84,673,700	115,896,391	59,953,219	25,555.38	34,978.70	18,094.49
Male	15-19 years	91,967,495	130,113,172	61,500,600	28,940.43	40,944.15	19,353.07
Male	20-24 years	92,819,750	128,363,682	63,020,437	30,495.87	42,173.81	20,705.33
Male	25-29 years	98,154,811	133,669,684	68,939,346	32,205.45	43,858.19	22,619.60
Male	30-34 years	98,693,765	132,528,859	66,996,655	32,553.44	43,713.70	22,098.37
Male	35-39 years	95,870,259	128,851,411	69,207,789	35,176.55	47,277.94	25,393.61
Male	40-44 years	79,719,148	107,565,706	55,113,283	32,048.60	43,243.44	22,156.58
Male	45-49 years	64,848,632	93,969,136	44,303,750	27,204.78	39,421.18	18,585.95
Male	50-54 years	60,632,318	83,132,091	42,174,757	27,865.91	38,206.55	19,383.03
Female	10-14 years	83,549,637	113,754,260	59,392,537	26,877.58	36,594.29	19,106.34
Female	15-19 years	92,384,815	129,505,177	62,162,656	30,615.44	42,916.78	20,600.11
Female	20-24 years	95,952,923	131,991,420	65,393,245	32,441.05	44,625.43	22,109.03
Female	25-29 years	101,774,506	138,568,965	71,950,482	33,846.64	46,083.19	23,928.21
Female	30-34 years	100,258,361	134,849,635	68,535,183	33,580.88	45,167.01	22,955.41
Female	35-39 years	96,931,881	129,265,222	69,366,239	36,109.88	48,154.97	25,840.89
Female	40-44 years	81,689,919	109,645,223	57,397,100	33,383.90	44,808.28	23,456.25
Female	45-49 years	67,643,740	97,227,882	46,577,003	28,732.14	41,298.20	19,783.90
Female	50-54 years	65,346,535	89,075,405	45,127,730	29,807.18	40,630.87	20,584.57
TTH		Incidence					
		Number (95% UI)			Rate (95% UI)		
**Gender**	**Age**	**Mean**	**Upper**	**Lower**	**Mean**	**Upper**	**Lower**
Male	10-14 years	34,674,616	48,119,857	23,843,483	10,465.15	14,523.06	7,196.20
Male	15-19 years	31,556,379	44,665,658	19,937,720	9,930.19	14,055.44	6,274.02
Male	20-24 years	32,611,086	43,781,499	21,340,195	10,714.35	14,384.39	7,011.31

Back to Top

Male	25-29 years	32,999,660	47,089,273	21,412,907	10,827.48	15,450.40	7,025.76
Male	30-34 years	34,722,133	48,512,765	21,171,497	11,452.85	16,001.59	6,983.27
Male	35-39 years	32,114,323	43,786,105	20,575,546	11,783.33	16,065.92	7,549.54
Male	40-44 years	23,356,695	33,612,022	14,808,573	9,389.83	13,512.67	5,953.33
Male	45-49 years	21,912,489	32,424,211	13,751,759	9192.55	13602.34	5769.03
Male	50-54 years	18,693,652	26,677,346	12,365,208	8591.39	12260.6	5682.91
Female	10-14 years	34,106,069	47,393,295	23,613,338	10,971.78	15,246.23	7,596.32
Female	15-19 years	31,681,490	44,755,265	20,073,152	10,498.94	14,831.47	6,652.05
Female	20-24 years	33,340,731	44,548,185	21,950,763	11,272.28	15,061.45	7,421.41
Female	25-29 years	33,492,734	47,586,354	21,850,391	11,138.51	15,825.56	7,266.68
Female	30-34 years	34,727,845	48,613,565	20,980,215	11,631.87	16,282.80	7,027.19
Female	35-39 years	32,326,326	44,153,237	20,534,565	12,042.47	16,448.34	7,649.71
Female	40-44 years	23,745,491	34,070,939	14,901,188	9,703.98	13,923.64	6,089.61
Female	45-49 years	22,701,163	33,107,902	14,364,576	9642.47	14062.81	6101.45
Female	50-54 years	19,987,570	28,035,267	13,365,753	9117.13	12788.01	6096.66

**TTH**		**Years Lived with Disability**					
		**Number (95% UI)**			**Rate (95% UI)**		
**Gender**	**Age**	**Mean**	**Upper**	**Lower**	**Mean**	**Upper**	**Lower**
Male	10-14 years	97,866	654,182	8,406	29.54	197.44	2.54
Male	15-19 years	143,894	729,106	26,145	45.28	229.44	8.23
Male	20-24 years	163,818	723,780	35,868	53.82	237.8	11.78
Male	25-29 years	186,840	777,903	41,121	61.3	255.24	13.49
Male	30-34 years	203,694	767,349	48,098	67.19	253.1	15.86
Male	35-39 years	205,725	727,464	51,266	75.48	266.92	18.81
Male	40-44 years	192,323	617,899	53,376	77.32	248.41	21.46
Male	45-49 years	181,195	535,653	56,453	76.01	224.71	23.68
Male	50-54 years	168,448	506,839	48,818	77.42	232.94	22.44
Female	10-14 years	105,310	576,103	11,107	33.88	185.33	3.57
Female	15-19 years	168,165	719,524	38,130	55.73	238.44	12.64
Female	20-24 years	199,784	735,601	51,410	67.55	248.7	17.38

Back to Top

Female	25-29 years	226,116	764,158	56,853	75.2	254.13	18.91
Female	30-34 years	244,428	769,309	66,265	81.87	257.68	22.2
Female	35-39 years	250,466	741,506	69,851	93.31	276.23	26.02
Female	40-44 years	239,059	647,984	73,116	97.7	264.81	29.88
Female	45-49 years	228,157	588,394	78,003	96.91	249.92	33.13
Female	50-54 years	215,820	573,909	69,043	98.44	261.78	31.49

Back to Top

Supplementary Table 10. Global Changing trends of ratios of female YLD rates to male YLD rates of overall headache disorders, migraine, and TTH in adolescents and young adults from 1990 to 2019			
Year	Ratio of female YLD rates to male YLD rates		
	Headache Disorders	Migraine	TTH
1990	1.700	1.751	1.267
1991	1.699	1.751	1.266
1992	1.699	1.750	1.265
1993	1.698	1.750	1.265
1994	1.697	1.749	1.264
1995	1.696	1.748	1.263
1996	1.695	1.746	1.263
1997	1.694	1.745	1.263
1998	1.693	1.744	1.263
1999	1.692	1.743	1.263
2000	1.691	1.742	1.263
2001	1.690	1.741	1.263
2002	1.690	1.740	1.262
2003	1.689	1.741	1.261
2004	1.689	1.740	1.260
2005	1.688	1.739	1.259
2006	1.686	1.737	1.258
2007	1.685	1.735	1.258
2008	1.683	1.734	1.257
2009	1.681	1.732	1.257
2010	1.680	1.730	1.257
2011	1.678	1.728	1.256
2012	1.676	1.726	1.256
2013	1.674	1.723	1.255
2014	1.671	1.721	1.254
2015	1.669	1.719	1.254
2016	1.667	1.716	1.253
2017	1.666	1.715	1.253
2018	1.662	1.710	1.251
2019	1.655	1.702	1.247

Back to Top

Supplementary Table 11. Ratios of female YLD rates to male YLD rates of overall headache disorders, migraine, and TTH in adolescents and young adults by country and territory in 2019

Location	YLD Rate of Headache Disorders			YLD Rate of Migraine			YLD Rate of TTH		
	Female	Male	Ratio	Female	Male	Ratio	Female	Male	Ratio
Afghanistan	1026.96	647.2	1.587	944.78	583.09	1.620	82.18	64.11	1.282
Albania	993.01	544.4	1.824	885.85	468.8	1.890	107.16	75.61	1.417
Algeria	1118.44	671.99	1.664	1024.81	599.3	1.710	93.63	72.69	1.288
American Samoa	854.67	559.15	1.529	794.59	508.13	1.564	60.08	51.02	1.178
Andorra	1359.04	669	2.031	1,239.23	592.9	2.090	119.81	76.1	1.574
Angola	764.37	505.21	1.513	697.03	451.84	1.543	67.33	53.37	1.262
Antigua and Barbuda	956.51	515.71	1.855	881.44	460.31	1.915	75.07	55.4	1.355
Argentina	826.73	474.05	1.744	740.92	410.46	1.805	85.81	63.59	1.349
Armenia	971	547.77	1.773	873.79	474.41	1.842	97.2	73.36	1.325
Australia	941.58	569.47	1.653	852.73	504.03	1.692	88.85	65.44	1.358
Austria	1,305.04	660.5	1.976	1180.64	577.1	2.046	124.41	83.4	1.492
Azerbaijan	961.24	546.33	1.759	865.18	473.31	1.828	96.06	73.03	1.315
Bahamas	946.88	513.79	1.843	873.26	458.68	1.904	73.62	55.11	1.336
Bahrain	1133.19	682.34	1.661	1036.78	602.15	1.722	96.41	80.19	1.202
Bangladesh	893.17	602.83	1.482	828.92	549.31	1.509	64.25	53.52	1.200
Barbados	958.67	516.42	1.856	882.65	460.25	1.918	76.02	56.17	1.353
Belarus	1088.77	544.21	2.001	950.28	453.84	2.094	138.49	90.37	1.532
Belgium	1,659.62	786.78	2.109	1546.7	713.96	2.166	112.93	72.82	1.551
Belize	927.44	507.79	1.826	858.17	455.86	1.883	69.26	51.93	1.334
Benin	905.55	608.61	1.488	836.52	554.04	1.510	69.03	54.57	1.265
Bermuda	970.31	520.55	1.864	891.94	462.07	1.930	78.37	58.48	1.340
Bhutan	893.93	611.14	1.463	829.92	557.11	1.490	64.01	54.03	1.185
Bolivia (Plurinational State of)	760.52	389.08	1.955	694.33	341.94	2.031	66.19	47.14	1.404
Bosnia and Herzegovina	1,008.26	555.69	1.814	898.26	477.01	1.883	110	78.68	1.398
Botswana	803.1	535.88	1.499	729.87	477.42	1.529	73.22	58.46	1.252
Brazil	1234.45	687.63	1.795	1156.68	627.48	1.843	77.77	60.15	1.293
Brunei Darussalam	784.05	450.61	1.740	700.29	381.11	1.838	83.76	69.5	1.205
Bulgaria	1,020.21	563.95	1.809	908.45	483.72	1.878	111.77	80.23	1.393
Burkina Faso	908.45	604.29	1.503	839.02	550.34	1.525	69.43	53.95	1.287
Burundi	574.71	374.59	1.534	513.64	325.98	1.576	61.07	48.61	1.256
Cabo Verde	947.01	640.06	1.480	871.36	579.46	1.504	75.66	60.6	1.249
Cambodia	1,002.90	638.39	1.571	938.56	583.6	1.608	64.34	54.78	1.175
Cameroon	908.55	610.1	1.489	838.93	554.95	1.512	69.61	55.15	1.262
Canada	1306.14	538.65	2.425	1207.33	463	2.608	98.82	75.65	1.306

Back to Top

Central African Republic	767.72	506.66	1.515	700.12	452.75	1.546	67.6	53.91	1.254
Chad	888.78	594.79	1.494	822.26	542.38	1.516	66.53	52.41	1.269
Chile	903.44	451.93	1.999	815.78	387.02	2.108	87.66	64.91	1.350
China	813.25	507.93	1.601	755.48	453.06	1.668	57.77	54.87	1.053
Colombia	1004.93	491.95	2.043	931.68	437.91	2.128	73.25	54.03	1.356
Comoros	610.32	394.53	1.547	543.85	342.62	1.587	66.47	51.9	1.281
Congo	785.35	525.66	1.494	714.6	468.54	1.525	70.75	57.12	1.239
Cook Islands	884.24	577.91	1.530	821.03	524.18	1.566	63.21	53.73	1.176
Costa Rica	986.15	490.22	2.012	912.69	435.69	2.095	73.46	54.54	1.347
Cote d'Ivoire	916.22	617.44	1.484	845.45	560.65	1.508	70.77	56.8	1.246
Croatia	1,007.49	554.78	1.816	897.4	475.92	1.886	110.09	78.87	1.396
Cuba	961.58	517.44	1.858	884.07	460.21	1.921	77.52	57.23	1.355
Cyprus	1368.97	671.85	2.038	1251.91	597.76	2.094	117.06	74.09	1.580
Czechia	1,016.14	561.59	1.809	904.99	481.48	1.880	111.15	80.11	1.387
Democratic People's Republic of Korea	785.97	481.4	1.633	729.98	432.46	1.688	55.99	48.94	1.144
Democratic Republic of the Congo	760.07	507.34	1.498	693.37	453.56	1.529	66.69	53.79	1.240
Denmark	1178.62	595.9	1.978	1,068.69	522.3	2.046	109.92	73.6	1.493
Djibouti	626.25	396.93	1.578	558.05	344.41	1.620	68.2	52.53	1.298
Dominica	939.75	510.67	1.840	867.24	456.24	1.901	72.51	54.43	1.332
Dominican Republic	938.42	512.68	1.830	867.15	459.21	1.888	71.27	53.47	1.333
Ecuador	838.4	440	1.905	774.11	393.91	1.965	64.28	46.09	1.395
Egypt	1160.2	681.78	1.702	1068.02	609.64	1.752	92.18	72.14	1.278
El Salvador	974.67	485.21	2.009	903.13	433.45	2.084	71.54	51.76	1.382
Equatorial Guinea	770.02	503.41	1.530	701.93	451.02	1.556	68.09	52.38	1.300
Eritrea	590.08	382.2	1.544	526.84	332.76	1.583	63.24	49.45	1.279
Estonia	1078.74	543.24	1.986	941.82	452.77	2.080	136.92	90.47	1.513
Eswatini	781.2	514	1.520	711.51	459.09	1.550	69.68	54.92	1.269
Ethiopia	510.41	329.53	1.549	460.17	291.24	1.580	50.24	38.29	1.312
Fiji	874.33	577.51	1.514	812.46	524.52	1.549	61.87	52.99	1.168
Finland	1327.18	657.05	2.020	1,215.15	584.23	2.080	112.04	72.82	1.539
France	1292.83	683.74	1.891	1172.14	606.71	1.932	120.69	77.03	1.567
Gabon	788.97	527.21	1.497	717.88	469.97	1.528	71.08	57.24	1.242
Gambia	901.85	611.31	1.475	833.28	556.33	1.498	68.56	54.98	1.247
Georgia	966.87	547.18	1.767	869.54	473.43	1.837	97.34	73.76	1.320
Germany	1485.41	708.74	2.096	1368.36	634.46	2.157	117.06	74.28	1.576
Ghana	929.05	621.68	1.494	855.88	564.63	1.516	73.17	57.05	1.283
Greece	1381.13	707.25	1.953	1263.79	632.45	1.998	117.35	74.8	1.569

Back to Top

Greenland	1252.6	556.59	2.250	1157.75	481.98	2.402	94.85	74.6	1.271
Grenada	943.89	513.41	1.838	870.77	458.99	1.897	73.12	54.42	1.344
Guam	879.59	581.3	1.513	816.95	527.71	1.548	62.64	53.59	1.169
Guatemala	959.54	484.42	1.981	890.73	434.02	2.052	68.82	50.4	1.365
Guinea	906.48	603.18	1.503	837.43	549.46	1.524	69.04	53.71	1.285
Guinea-Bissau	911.2	610.34	1.493	841.29	555.61	1.514	69.92	54.73	1.278
Guyana	928.4	505.33	1.837	857.93	452.91	1.894	70.47	52.42	1.344
Haiti	917.96	503.7	1.822	849.49	452.76	1.876	68.47	50.94	1.344
Honduras	957.16	484.51	1.976	888.82	433.99	2.048	68.34	50.52	1.353
Hungary	1018.34	562.01	1.812	907.01	482.26	1.881	111.34	79.75	1.396
Iceland	1325.91	655.82	2.022	1215.69	583.78	2.082	110.22	72.04	1.530
India	923.92	604.47	1.528	855.89	548.32	1.561	68.03	56.16	1.211
Indonesia	1,043.33	676.6	1.542	972.75	616	1.579	70.58	60.59	1.165
Iran (Islamic Republic of)	1189.84	725.69	1.640	1084.66	633.32	1.713	105.18	92.37	1.139
Iraq	1077.37	662.23	1.627	989.35	593.63	1.667	88.03	68.6	1.283
Ireland	1324.47	652.27	2.031	1211.49	579.83	2.089	112.98	72.44	1.560
Israel	1303.93	644.96	2.022	1197.5	575.26	2.082	106.43	69.7	1.527
Italy	1547.9	769.23	2.012	1428.46	687.68	2.077	119.44	81.54	1.465
Jamaica	944.96	514.64	1.836	872.73	460.62	1.895	72.23	54.02	1.337
Japan	849.37	470.76	1.804	754.18	393.64	1.916	95.19	77.12	1.234
Jordan	1077.8	669.75	1.609	989.61	599.52	1.651	88.19	70.23	1.256
Kazakhstan	947.68	539.44	1.757	853.21	467.26	1.826	94.47	72.19	1.309
Kenya	623.96	403.7	1.546	555.89	350.69	1.585	68.08	53.01	1.284
Kiribati	857.48	561.46	1.527	797.66	510.93	1.561	59.82	50.53	1.184
Kuwait	1139.77	657.75	1.733	1042.14	586.98	1.775	97.63	70.76	1.380
Kyrgyzstan	929.95	530.72	1.752	838.7	460.74	1.820	91.24	69.98	1.304
Lao People's Democratic Republic	997.9	636.12	1.569	934.37	581.46	1.607	63.53	54.66	1.162
Latvia	1,083.51	544.01	1.992	945.47	453.12	2.087	138.04	90.89	1.519
Lebanon	1123.41	669.03	1.679	1028.52	596.97	1.723	94.89	72.06	1.317
Lesotho	781.61	524.01	1.492	711.37	467.51	1.522	70.23	56.49	1.243
Liberia	904.22	609.02	1.485	834.16	553.03	1.508	70.06	55.99	1.251
Libya	1115.52	670.91	1.663	1021.39	597.39	1.710	94.13	73.52	1.280
Lithuania	1,004.03	467.24	2.149	877.77	380.08	2.309	126.26	87.16	1.449
Luxembourg	1316.27	677.99	1.941	1190.54	599.03	1.987	125.73	78.96	1.592
Madagascar	592.09	381.89	1.550	528.58	332.36	1.590	63.51	49.53	1.282
Malawi	572.98	371.57	1.542	512.29	323.83	1.582	60.68	47.74	1.271
Malaysia	909.34	628.09	1.448	843.62	571.36	1.477	65.72	56.73	1.158
Maldives	1,033.77	685.83	1.507	966.82	625.69	1.545	66.96	60.14	1.113
Mali	897.64	601.61	1.492	830.07	548.28	1.514	67.57	53.33	1.267
Malta	1353.53	669.3	2.022	1238.4	594.82	2.082	115.13	74.48	1.546
Marshall Islands	862.13	571.53	1.508	801.7	519.66	1.543	60.43	51.87	1.165

Back to Top

Mauritania	910.85	610.25	1.493	840.82	555.14	1.515	70.03	55.11	1.271
Mauritius	1028.49	657.93	1.563	960.51	599.66	1.602	67.98	58.26	1.167
Mexico	991.01	508.88	1.947	913.66	451.6	2.023	77.35	57.27	1.351
Micronesia (Federated States of)	855.02	564.97	1.513	795.28	513.93	1.547	59.74	51.04	1.170
Monaco	1336.34	656.82	2.035	1218.98	582.39	2.093	117.36	74.43	1.577
Mongolia	955.23	541.03	1.766	860.26	468.86	1.835	94.96	72.18	1.316
Montenegro	998.6	549.59	1.817	890.32	472.36	1.885	108.28	77.23	1.402
Morocco	1101.79	667.65	1.650	1009.83	596.44	1.693	91.96	71.21	1.291
Mozambique	572.46	368.1	1.555	511.5	320.75	1.595	60.95	47.34	1.287
Myanmar	1,009.22	641	1.574	943.79	585.44	1.612	65.43	55.56	1.178
Namibia	790.73	524.19	1.508	719.5	467.82	1.538	71.23	56.37	1.264
Nauru	858.01	563.66	1.522	798.46	512.98	1.557	59.55	50.67	1.175
Nepal	923.42	632.16	1.461	857.02	577.78	1.483	66.39	54.38	1.221
Netherlands	1274.04	624.5	2.040	1168.21	551.32	2.119	105.84	73.18	1.446
New Zealand	972.37	587.81	1.654	878.92	519.79	1.691	93.45	68.02	1.374
Nicaragua	967.94	486.25	1.991	897.7	434.19	2.068	70.23	52.05	1.349
Niger	886.15	595.98	1.487	820.35	544.01	1.508	65.8	51.97	1.266
Nigeria	925.51	626.86	1.476	852.46	569.14	1.498	73.04	57.72	1.265
Niue	880.41	574.67	1.532	817.15	521.45	1.567	63.26	53.22	1.189
North Macedonia	1,010.44	559.74	1.805	900.83	480.72	1.874	109.61	79.01	1.387
Northern Mariana Islands	885.9	584.59	1.515	821.56	530.08	1.550	64.34	54.51	1.180
Norway	1432.69	709.39	2.020	1320.21	633.6	2.084	112.48	75.79	1.484
Oman	1125.92	688.16	1.636	1032.93	611.19	1.690	92.99	76.97	1.208
Pakistan	830.77	571.12	1.455	772.68	521.9	1.481	58.09	49.22	1.180
Palau	896.57	604.22	1.484	831.22	547.4	1.518	65.35	56.83	1.150
Palestine	1062.34	655.79	1.620	976.27	589.01	1.657	86.07	66.78	1.289
Panama	971.75	488.43	1.990	899.94	434.76	2.070	71.81	53.66	1.338
Papua New Guinea	858.77	561.36	1.530	799.17	510.97	1.564	59.59	50.39	1.183
Paraguay	1171.06	633.12	1.850	1101.76	578.88	1.903	69.3	54.23	1.278
Peru	705.5	352.54	2.001	634.37	303	2.094	71.13	49.55	1.436
Philippines	1,020.39	659.94	1.546	952.5	601.55	1.583	67.89	58.39	1.163
Poland	1,067.64	592.72	1.801	949.36	508.48	1.867	118.28	84.24	1.404
Portugal	1346.74	661.95	2.035	1229.69	587.81	2.092	117.05	74.14	1.579
Puerto Rico	957.06	514.85	1.859	881.33	459.09	1.920	75.73	55.76	1.358
Qatar	1144.85	691.42	1.656	1048.66	612.98	1.711	96.19	78.44	1.226
Republic of Korea	863.21	497.9	1.734	770.91	419.53	1.838	92.3	78.37	1.178
Republic of Moldova	1,080.40	543.33	1.988	944.13	453.33	2.083	136.28	90	1.514
Romania	1,011.49	559.91	1.807	900.93	480.26	1.876	110.56	79.65	1.388

Back to Top

Russian Federation	1185.44	633.23	1.872	1028.36	532.47	1.931	157.08	100.76	1.559
Rwanda	592.77	380.93	1.556	529.13	331.54	1.596	63.64	49.39	1.289
Saint Kitts and Nevis	953.36	515.65	1.849	878.82	459.54	1.912	74.53	56.1	1.329
Saint Lucia	951.43	515.33	1.846	876.93	459.65	1.908	74.5	55.69	1.338
Saint Vincent and the Grenadines	943.6	511.84	1.844	870.73	457.23	1.904	72.87	54.6	1.335
Samoa	843.22	555.51	1.518	784.9	505.52	1.553	58.32	49.99	1.167
San Marino	1334.66	652.95	2.044	1220.37	580.35	2.103	114.29	72.6	1.574
Sao Tome and Principe	919.5	621.36	1.480	848.14	564.16	1.503	71.37	57.21	1.248
Saudi Arabia	1080.08	691.54	1.562	987.1	617.93	1.597	92.97	73.61	1.263
Senegal	911.59	608.38	1.498	841.16	553.77	1.519	70.43	54.61	1.290
Serbia	1,002.82	551.18	1.819	894	473.65	1.887	108.82	77.53	1.404
Seychelles	1027.98	667.75	1.539	959.9	608.55	1.577	68.08	59.2	1.150
Sierra Leone	910.06	612.97	1.485	840.58	557.38	1.508	69.48	55.59	1.250
Singapore	755.07	453.92	1.663	666	379.24	1.756	89.07	74.68	1.193
Slovakia	1019.23	561.27	1.816	908.27	481.77	1.885	110.96	79.5	1.396
Slovenia	1,017.31	563.74	1.805	905.67	483.23	1.874	111.65	80.51	1.387
Solomon Islands	849.91	558.14	1.523	791.1	508.13	1.557	58.81	50.01	1.176
Somalia	574.89	372.94	1.542	514.02	325.29	1.580	60.87	47.65	1.277
South Africa	844.58	568.67	1.485	764.87	505.18	1.514	79.71	63.49	1.255
South Sudan	576.09	363.46	1.585	514.18	316.32	1.626	61.91	47.14	1.313
Spain	1405.54	677.98	2.073	1275.95	603.41	2.115	129.59	74.57	1.738
Sri Lanka	1,018.41	646.31	1.576	951.6	589.42	1.614	66.81	56.89	1.174
Sudan	1068.91	658.83	1.622	982.65	592.68	1.658	86.25	66.14	1.304
Suriname	936.29	508.18	1.842	863.88	454.38	1.901	72.41	53.8	1.346
Sweden	1333.51	637.69	2.091	1226.38	569.03	2.155	107.13	68.66	1.560
Switzerland	1229.46	605.05	2.032	1117.39	534.14	2.092	112.07	70.91	1.580
Syrian Arab Republic	1070.47	651.15	1.644	982.07	583.54	1.683	88.4	67.61	1.307
Taiwan (Province of China)	884.48	522.03	1.694	822.64	473.61	1.737	61.85	48.42	1.277
Tajikistan	918.96	524.04	1.754	829.58	455.13	1.823	89.38	68.91	1.297
Thailand	1,177.83	699.88	1.683	1,107.45	640.13	1.730	70.38	59.75	1.178
Timor-Leste	965.22	608.29	1.587	905.01	556.62	1.626	60.21	51.66	1.166
Togo	923.42	613.16	1.506	851.14	557.31	1.527	72.28	55.85	1.294
Tokelau	871.36	569.26	1.531	809.87	517.23	1.566	61.49	52.03	1.182
Tonga	856.39	556.91	1.538	796.46	506.58	1.572	59.93	50.33	1.191
Trinidad and Tobago	953.73	516.58	1.846	878.95	460.35	1.909	74.78	56.22	1.330
Tunisia	1128.59	670.95	1.682	1032.93	597.47	1.729	95.66	73.47	1.302

Back to Top

Turkey	1148.7	617.01	1.862	1051.91	545.66	1.928	96.79	71.35	1.357
Turkmenistan	936.83	536.76	1.745	844.27	465.47	1.814	92.56	71.29	1.298
Tuvalu	862.15	571.5	1.509	801.99	519.7	1.543	60.17	51.8	1.162
Uganda	574.43	367.72	1.562	513.52	320.64	1.602	60.91	47.09	1.293
Ukraine	1137.53	575.41	1.977	991.72	479.73	2.067	145.8	95.68	1.524
United Arab Emirates	1154.99	687.16	1.681	1055.91	603.62	1.749	99.08	83.54	1.186
United Kingdom	1308.38	645.66	2.026	1193.09	570.29	2.092	115.29	75.37	1.530
United Republic of Tanzania	618.2	368.53	1.677	555.41	319.65	1.738	62.79	48.88	1.285
United States of America	1251.44	602.31	2.078	1150.62	524.75	2.193	100.82	77.56	1.300
United States Virgin Islands	953.02	511.78	1.862	877.35	456.09	1.924	75.67	55.7	1.359
Uruguay	862.22	461.74	1.867	775.3	397.67	1.950	86.92	64.07	1.357
Uzbekistan	929.59	532.53	1.746	838.14	462.23	1.813	91.44	70.3	1.301
Vanuatu	847.4	557.64	1.520	788.9	507.52	1.554	58.5	50.12	1.167
Venezuela (Bolivarian Republic of)	985.22	481.9	2.044	912.28	427.48	2.134	72.94	54.42	1.340
Viet Nam	1,041.01	662.47	1.571	972.76	604.27	1.610	68.25	58.2	1.173
Yemen	1049.44	656.95	1.597	965.31	591.04	1.633	84.13	65.92	1.276
Zambia	631.09	425.51	1.483	560.69	370.22	1.514	70.39	55.3	1.273
Zimbabwe	778.44	514.17	1.514	709.53	459.58	1.544	68.91	54.58	1.263

Back to Top

Supplementary Table 12. The YLD rate of overall headache disorders in adolescents and young adults in five major socio-demographic regions from 1990 to 2019					
**Headache Disorders**	**Socio-demographic Index**				
**Year**	**Low**	**Low-middle**	**Middle**	**High-middle**	**High**
1990	650.05	719.35	703.62	745.5	864.7
1991	649.66	719.98	706.34	745.99	866.7
1992	649.7	720.46	708.89	746.25	867.84
1993	649.58	720.91	711.27	746.46	868.6
1994	649.17	721.46	713.52	746.47	868.9
1995	648.81	721.92	715.51	746.43	869.13
1996	648.27	721.85	716.01	746.57	868.3
1997	647.69	721.09	714.88	747.2	866.23
1998	647.08	720.05	713.2	748.23	863.58
1999	646.72	719.47	712.04	749.4	861.39
2000	646.92	719.83	712.55	750.74	860.04
2001	647.11	720.97	715.7	753.02	859.36
2002	647.1	722.41	720.82	756.72	858.93
2003	647.03	723.91	726.92	761.23	858.64
2004	647.04	725.44	732.69	765.6	858.75
2005	647.2	726.71	737.19	768.55	859.17
2006	647.66	727.79	740.88	771.06	860.42
2007	648.29	729.23	744.7	773.57	862.19
2008	649.19	730.47	748.34	776.23	863.89
2009	649.85	731.72	751.51	778.41	865.52
2010	650.5	732.71	754.05	780.06	866.6
2011	650.53	733.09	755.87	781.33	868.41
2012	650.41	732.74	757.19	782.43	871.67
2013	650.24	732.11	758.05	783.4	875.17
2014	650.08	731.82	758.72	784.05	878.03
2015	650.23	732.16	759.61	784.27	879.14
2016	650.62	733.27	760.66	784.16	876.55
2017	650.95	734.4	761.7	783.95	873.93
2018	652.49	738.55	765.39	785.88	874.08
2019	655.44	746.68	772.22	790.21	875.22

Back to Top

Supplementary Table 13. The YLD rate of migraine in adolescents and young adults in five major socio-demographic regions from 1990 to 2019					
**Migraine**	**Socio-demographic Index**				
**Year**	**Low**	**Low-middle**	**Middle**	**High-middle**	**High**
1990	592.25	661.32	645.57	670.2	779.09
1991	591.9	661.89	648.01	670.71	780.93
1992	591.92	662.31	650.32	671.08	781.91
1993	591.83	662.71	652.46	671.43	782.52
1994	591.46	663.21	654.47	671.59	782.65
1995	591.11	663.63	656.24	671.71	782.69
1996	590.62	663.57	656.64	671.97	781.64
1997	590.11	662.93	655.54	672.71	779.36
1998	589.58	662.03	653.93	673.72	776.53
1999	589.27	661.52	652.77	674.83	774.18
2000	589.47	661.86	653.12	675.99	772.69
2001	589.71	662.86	655.92	678	771.93
2002	589.84	664.19	660.57	681.38	771.59
2003	589.94	665.57	666.13	685.5	771.39
2004	590.08	666.97	671.41	689.53	771.57
2005	590.26	668.07	675.5	692.29	772
2006	590.68	669.01	678.85	694.63	773.2
2007	591.25	670.3	682.36	696.98	774.92
2008	592.08	671.39	685.67	699.47	776.58
2009	592.69	672.49	688.55	701.49	778.15
2010	593.28	673.31	690.81	703.03	779.19
2011	593.27	673.55	692.38	704.22	780.95
2012	593.06	673.04	693.46	705.28	784.11
2013	592.84	672.25	694.13	706.21	787.55
2014	592.64	671.79	694.61	706.86	790.34
2015	592.74	672	695.33	707.08	791.39
2016	593.05	672.91	696.17	706.9	788.76
2017	593.32	673.83	697.01	706.58	786.14
2018	594.67	677.59	700.42	708.53	786.39
2019	597.32	685.06	706.84	712.99	787.67

Back to Top

Supplementary Table 14. The YLD rate of TTH in adolescents and young adults in five major socio-demographic regions from 1990 to 2019					
**TTH**	**Socio-demographic Index**				
**Year**	**Low**	**Low-middle**	**Middle**	**High-middle**	**High**
1990	57.8	58.02	58.05	75.31	85.61
1991	57.76	58.1	58.33	75.28	85.77
1992	57.78	58.15	58.57	75.18	85.93
1993	57.74	58.2	58.81	75.03	86.09
1994	57.71	58.25	59.05	74.88	86.25
1995	57.7	58.29	59.26	74.72	86.44
1996	57.65	58.29	59.37	74.6	86.65
1997	57.58	58.16	59.33	74.48	86.87
1998	57.5	58.03	59.27	74.51	87.05
1999	57.45	57.95	59.27	74.56	87.21
2000	57.45	57.97	59.43	74.75	87.35
2001	57.4	58.11	59.78	75.03	87.43
2002	57.26	58.22	60.25	75.34	87.34
2003	57.1	58.34	60.79	75.73	87.25
2004	56.96	58.47	61.29	76.07	87.18
2005	56.93	58.64	61.69	76.26	87.17
2006	56.98	58.78	62.04	76.42	87.23
2007	57.04	58.93	62.35	76.59	87.27
2008	57.11	59.09	62.67	76.76	87.31
2009	57.16	59.23	62.96	76.93	87.37
2010	57.22	59.4	63.25	77.03	87.41
2011	57.27	59.54	63.49	77.12	87.46
2012	57.34	59.7	63.73	77.15	87.56
2013	57.4	59.86	63.93	77.2	87.62
2014	57.45	60.02	64.1	77.19	87.69
2015	57.5	60.16	64.28	77.19	87.76
2016	57.57	60.36	64.49	77.26	87.78
2017	57.63	60.57	64.69	77.37	87.79
2018	57.83	60.96	64.97	77.35	87.69
2019	58.12	61.61	65.37	77.22	87.56

Back to Top

Supplementary Table 15. The associations between overall headache disorders burden in adolescents and young adults and socio-demographic factor by regions from 1990 to 2019								
Year	Andean Latin America		Australasia		Caribbean		Central Asia	
	YLD Rate	SDI	YLD Rate	SDI	YLD Rate	SDI	YLD Rate	SDI
1990	521.82	0.489	749.13	0.742	719.36	0.517	710.25	0.551
1991	521.82	0.492	750.42	0.746	720.49	0.522	711.04	0.555
1992	522.68	0.496	751.96	0.749	721.26	0.525	711.54	0.557
1993	523.23	0.5	752.72	0.753	721.75	0.528	711.95	0.558
1994	523.99	0.504	753.5	0.757	722.07	0.531	712.06	0.559
1995	524.72	0.509	754.56	0.761	722.12	0.533	711.97	0.559
1996	527.53	0.514	755.55	0.765	722.19	0.536	711.61	0.56
1997	534.38	0.519	756.09	0.769	722.51	0.539	711.49	0.56
1998	541.89	0.524	756.62	0.773	722.72	0.542	711.04	0.561
1999	548.7	0.529	756.66	0.777	722.44	0.547	711.09	0.563
2000	551.85	0.534	756.85	0.781	722.51	0.552	711.86	0.566
2001	552.5	0.538	756.49	0.785	722.67	0.557	712.22	0.569
2002	554.32	0.543	756.59	0.789	723.03	0.563	713.34	0.574
2003	555.94	0.548	756.22	0.793	724.14	0.569	714.92	0.579
2004	557.09	0.553	755.92	0.797	724.67	0.573	716.42	0.585
2005	558.48	0.558	756.67	0.799	725.24	0.579	718.25	0.591
2006	559.4	0.564	756.88	0.799	725.75	0.585	720.35	0.598
2007	560.3	0.569	757.33	0.8	726.26	0.588	722.41	0.605
2008	561.73	0.575	757.76	0.803	726.85	0.591	724.61	0.611
2009	562.55	0.58	757.86	0.807	726.7	0.594	726.68	0.617
2010	563.28	0.585	758.49	0.81	726.89	0.598	728.95	0.622
2011	563.74	0.591	758.79	0.812	726.56	0.601	731.13	0.627
2012	564.47	0.596	759.89	0.816	726.69	0.605	733.28	0.632
2013	564.93	0.602	760.14	0.821	727.17	0.609	734.92	0.637
2014	565.73	0.608	760.72	0.825	726.93	0.612	736.27	0.642
2015	565.86	0.613	761.05	0.828	727.23	0.616	737.25	0.647

Back to Top

2016	566.3	0.618	761.49	0.832	727.37	0.621	737.59	0.651
2017	566.44	0.624	761.13	0.835	727.62	0.625	737.56	0.655
2018	567.29	0.628	760.72	0.837	727.45	0.628	737.42	0.659
2019	567.76	0.632	760.31	0.84	727.42	0.631	736.97	0.663

Supplementary Table 15 (continued).

Year	Central Europe		Central Latin America		Central Sub-Saharan Africa		East Asia	
	YLD Rate	SDI	YLD Rate	SDI	YLD Rate	SDI	YLD Rate	SDI
1990	761.12	0.641	715.38	0.485	628.39	0.269	572.3	0.447
1991	761.69	0.647	716.78	0.49	628.37	0.273	575.64	0.456
1992	762.47	0.652	718.06	0.495	628.75	0.276	578.47	0.464
1993	763.65	0.658	719.42	0.501	628.37	0.279	581.11	0.473
1994	764.79	0.665	720.84	0.507	628.25	0.281	583.33	0.483
1995	766.03	0.672	722.17	0.512	627.95	0.283	584.95	0.492
1996	767.27	0.678	723.35	0.517	627.79	0.286	584.55	0.502
1997	768.22	0.683	724.35	0.523	627.79	0.289	582.04	0.511
1998	769.45	0.689	725.38	0.528	627.34	0.292	578.68	0.52
1999	770.96	0.695	726.21	0.533	627.32	0.295	575.98	0.528
2000	772.42	0.702	727.04	0.538	627.74	0.298	575.53	0.537
2001	774.07	0.709	727.51	0.543	627.69	0.302	579.55	0.545
2002	775.43	0.715	727.91	0.547	627.79	0.308	587.7	0.554
2003	777.04	0.72	728.16	0.551	628.48	0.313	597.71	0.562
2004	778.94	0.726	728.89	0.554	628.94	0.32	606.99	0.571
2005	780.65	0.731	729.35	0.559	629.27	0.328	613.53	0.58
2006	782.51	0.736	730.92	0.564	630.14	0.336	617.9	0.59
2007	784.11	0.74	733.84	0.569	630.62	0.346	622.09	0.6
2008	785.84	0.745	737.34	0.575	631.88	0.357	626.18	0.609
2009	787.43	0.75	740.26	0.579	632.65	0.367	629.56	0.618
2010	788.9	0.756	741.98	0.584	633.38	0.378	632.11	0.628
2011	790.37	0.76	742.74	0.589	634.1	0.389	633.96	0.637
2012	791.53	0.764	743.61	0.594	634.56	0.4	635.52	0.644
2013	792.97	0.768	744.32	0.599	635.1	0.412	636.91	0.652

Back to Top

2014	794.02	0.771	745.17	0.604	635.6	0.423	638.08	0.66
2015	795.08	0.775	746.04	0.609	635.58	0.434	639.06	0.662
2016	796.11	0.778	746.4	0.614	635.78	0.445	639.85	0.665
2017	796.82	0.781	747.12	0.618	636.04	0.454	640.56	0.675
2018	796.73	0.785	747.58	0.623	635.87	0.463	645.69	0.684
2019	796.34	0.788	748.12	0.626	635.93	0.47	656.56	0.691

Supplementary Table 15 (continued).

Year	Eastern Europe		Eastern Sub-Saharan Africa		High-income Asia Pacific		High-income North America	
	YLD Rate	SDI	YLD Rate	SDI	YLD Rate	SDI	YLD Rate	SDI
1990	855.51	0.68	461.27	0.235	637.95	0.767	954.02	0.771
1991	855.68	0.687	460.13	0.239	639.41	0.773	952.77	0.773
1992	854.95	0.697	459.25	0.242	640.22	0.779	950.29	0.777
1993	853.4	0.702	458.72	0.245	641.46	0.785	947.06	0.78
1994	851.78	0.702	458.3	0.249	642.68	0.79	943.9	0.784
1995	850.68	0.705	458.25	0.252	644.47	0.796	940.69	0.787
1996	851.35	0.707	458.28	0.257	645.69	0.801	935.12	0.79
1997	854.67	0.708	458.36	0.261	645.61	0.805	926.27	0.792
1998	860.9	0.709	458.48	0.265	645.39	0.809	916.56	0.794
1999	867.61	0.711	458.71	0.27	645.37	0.813	908.22	0.797
2000	872.87	0.711	459.13	0.275	645.87	0.816	903.41	0.8
2001	876.89	0.713	459.39	0.28	646.2	0.819	900.88	0.805
2002	880.69	0.716	459.55	0.285	645.73	0.823	898.6	0.809
2003	885.17	0.72	459.63	0.29	645.24	0.826	896.58	0.811
2004	889.5	0.727	459.75	0.295	645.03	0.83	895.37	0.814
2005	893.13	0.734	460.15	0.301	645.49	0.833	895.23	0.815
2006	896.95	0.74	460.91	0.307	647.18	0.836	896.33	0.814
2007	900.73	0.745	461.96	0.314	649.61	0.839	897.79	0.817
2008	903.87	0.751	463.27	0.321	651.69	0.842	899.64	0.822
2009	907.01	0.757	464.62	0.328	653.65	0.844	901.57	0.828
2010	909.52	0.762	465.73	0.336	654.51	0.847	903.19	0.834
2011	911.61	0.765	466.47	0.343	655.23	0.85	907.06	0.837

Back to Top

2012	912.94	0.768	467.33	0.351	656.43	0.853	913.8	0.841
2013	913.79	0.772	468.11	0.359	657.5	0.856	921.11	0.844
2014	913.86	0.777	468.9	0.367	658.79	0.859	927.08	0.847
2015	913.68	0.781	469.69	0.375	659.62	0.862	929.34	0.85
2016	913.11	0.785	470.3	0.383	659.93	0.865	928.09	0.854
2017	912.02	0.788	470.97	0.391	659.79	0.868	926.79	0.857
2018	905.05	0.791	471.82	0.399	659.7	0.871	926.13	0.859
2019	891.19	0.793	472.47	0.405	659.6	0.873	925.12	0.86

Supplementary Table 15 (continued).

Year	North Africa and Middle East		Oceania		South Asia		Southeast Asia	
	YLD Rate	SDI	YLD Rate	SDI	YLD Rate	SDI	YLD Rate	SDI
1990	853.73	0.414	688.77	0.383	733.28	0.313	830.13	0.455
1991	854	0.423	689.32	0.385	733.51	0.319	831.37	0.463
1992	854.25	0.432	689.84	0.388	733.46	0.325	832.89	0.471
1993	854.63	0.441	690.42	0.391	733.36	0.331	834.28	0.479
1994	855.24	0.45	690.88	0.394	733.43	0.337	835.64	0.488
1995	856.3	0.46	691.53	0.397	733.49	0.344	837.06	0.496
1996	856.36	0.469	692.5	0.4	732.61	0.351	837.24	0.504
1997	855.4	0.478	693.16	0.403	730.37	0.357	835.51	0.512
1998	854.2	0.487	694.28	0.405	727.59	0.364	833.52	0.517
1999	853.57	0.497	695.26	0.408	725.57	0.371	832.09	0.523
2000	854.15	0.506	695.63	0.41	725.29	0.378	832.26	0.528
2001	856.3	0.515	696.29	0.412	725.97	0.385	833.56	0.533
2002	859.33	0.524	696.41	0.413	726.6	0.391	835.03	0.538
2003	863.12	0.532	696.32	0.414	727.21	0.398	836.52	0.543
2004	866.45	0.541	696.82	0.416	728.04	0.405	837.9	0.548
2005	869.17	0.55	697.61	0.417	728.87	0.413	839.22	0.554
2006	871.01	0.56	697.79	0.419	729.8	0.421	840.67	0.559
2007	872.81	0.569	698.02	0.421	731	0.43	842.44	0.566
2008	874.44	0.578	698.79	0.422	732.1	0.438	843.93	0.572
2009	875.98	0.587	698.66	0.424	733.13	0.447	845.58	0.578

Back to Top

2010	877.26	0.595	699.52	0.426	734.16	0.456	846.59	0.585
2011	878.42	0.603	700.5	0.428	734.07	0.465	847.6	0.592
2012	879.66	0.611	702.28	0.431	732.9	0.475	848.5	0.599
2013	880.7	0.619	703.72	0.433	731.28	0.485	849.43	0.606
2014	881.73	0.626	704.6	0.436	730.12	0.495	849.95	0.612
2015	882.18	0.633	705.72	0.44	730.22	0.505	850.66	0.619
2016	880.98	0.64	706.01	0.443	731.16	0.515	851.3	0.626
2017	879.6	0.647	706.52	0.446	732.05	0.525	851.49	0.632
2018	881.38	0.654	706.98	0.449	738.64	0.535	851.98	0.639
2019	885.05	0.66	707.2	0.452	751.93	0.543	852.31	0.644

Supplementary Table 15 (continued).

Year	Southern Latin America		Southern Sub-Saharan Africa		Tropical Latin America		Western Europe	
	YLD Rate	SDI	YLD Rate	SDI	YLD Rate	SDI	YLD Rate	SDI
1990	617.48	0.584	667.44	0.513	935.09	0.487	1003.3	0.75
1991	617.67	0.589	668.03	0.517	931.51	0.492	1006.7	0.756
1992	618.22	0.597	668.63	0.522	928.6	0.497	1010.1	0.762
1993	618.53	0.602	669.32	0.526	926.73	0.503	1013.4	0.767
1994	619.44	0.608	669.4	0.53	925.7	0.508	1016	0.772
1995	619.86	0.614	669.78	0.535	925.48	0.513	1018.3	0.775
1996	620.27	0.62	669.6	0.539	929.27	0.519	1022.2	0.779
1997	620.68	0.625	669.87	0.544	937.48	0.524	1029.3	0.782
1998	620.8	0.63	669.71	0.548	947.59	0.529	1036.8	0.784
1999	620.68	0.634	669.75	0.553	956.99	0.533	1043.1	0.787
2000	621.2	0.64	669.93	0.557	963.23	0.538	1045.8	0.79
2001	622.02	0.644	670.23	0.562	967.5	0.543	1045.7	0.794
2002	623.51	0.648	670.96	0.567	971.41	0.547	1045.2	0.797
2003	625.32	0.651	671.78	0.571	974.99	0.552	1044.5	0.8
2004	626.84	0.653	672.79	0.576	977.07	0.556	1044.1	0.802
2005	628.42	0.658	673.88	0.581	977.05	0.561	1044.3	0.805
2006	631	0.663	675.26	0.586	975.66	0.566	1045.3	0.807
2007	636.19	0.664	676.94	0.591	973.69	0.572	1047.1	0.81

Back to Top

2008	642.76	0.667	678.71	0.596	971.49	0.578	1048.7	0.812
2009	647.8	0.671	680.66	0.601	969.07	0.584	1049.9	0.815
2010	650.74	0.676	682.42	0.605	966.8	0.59	1050.2	0.817
2011	651.93	0.681	684.05	0.61	964.49	0.597	1050.9	0.821
2012	652.99	0.686	685.73	0.614	961.75	0.604	1052.7	0.824
2013	653.92	0.689	687.22	0.619	958.94	0.61	1054.8	0.827
2014	655.2	0.692	688.33	0.623	956.51	0.617	1055.7	0.83
2015	656.16	0.701	689.4	0.628	954.26	0.622	1054.8	0.832
2016	657.1	0.71	689.94	0.632	958.32	0.627	1047.8	0.835
2017	658.13	0.716	690.67	0.636	963.03	0.632	1040.8	0.838
2018	658.7	0.719	691.21	0.639	962.48	0.636	1037.9	0.841
2019	659.7	0.721	691.58	0.642	962.21	0.64	1035.6	0.843

Supplementary Table 15 (continued).

Year	Western Sub-Saharan Africa	
	**YLD Rate**	**SDI**
1990	770.55	0.268
1991	770.66	0.272
1992	770.6	0.276
1993	770.66	0.28
1994	770.8	0.284
1995	770.85	0.288
1996	771.22	0.292
1997	771.92	0.297
1998	772.94	0.301
1999	773.78	0.306
2000	774.36	0.311
2001	774.34	0.316
2002	773.82	0.322
2003	773.05	0.328
2004	772.49	0.335
2005	772.49	0.343

Back to Top

2006	772.99	0.351
2007	773.68	0.359
2008	774.44	0.367
2009	775.03	0.375
2010	775.44	0.383
2011	775.69	0.391
2012	775.83	0.399
2013	775.75	0.407
2014	775.69	0.415
2015	775.39	0.422
2016	775.23	0.43
2017	775.21	0.437
2018	774.31	0.443
2019	772.4	0.448

Back to Top

Supplementary Table 16. The associations between migraine burden in adolescents and young adults and socio-demographic factor by regions from 1990 to 2019								
Year	Andean Latin America		Australasia		Caribbean		Central Asia	
	YLD Rate	SDI	YLD Rate	SDI	YLD Rate	SDI	YLD Rate	SDI
1990	468.01	0.489	674.22	0.742	659.26	0.517	632.67	0.551
1991	467.92	0.492	675.24	0.746	660.19	0.522	633.41	0.555
1992	468.66	0.496	676.49	0.749	660.79	0.525	633.93	0.557
1993	469.09	0.5	676.86	0.753	661.09	0.528	634.38	0.558
1994	469.78	0.504	677.49	0.757	661.31	0.531	634.54	0.559
1995	470.42	0.509	678.3	0.761	661.24	0.533	634.53	0.559
1996	473.06	0.514	679.1	0.765	661.21	0.536	634.24	0.56
1997	479.74	0.519	679.4	0.769	661.38	0.539	634.2	0.56
1998	487.07	0.524	679.72	0.773	661.51	0.542	633.68	0.561
1999	493.7	0.529	679.61	0.777	661.15	0.547	633.63	0.563
2000	496.67	0.534	679.62	0.781	661.16	0.552	634.27	0.566
2001	497.11	0.538	679.17	0.785	661.21	0.557	634.46	0.569
2002	498.67	0.543	679.18	0.789	661.45	0.563	635.34	0.574
2003	500.03	0.548	678.82	0.793	662.43	0.569	636.67	0.579
2004	501.01	0.553	678.56	0.797	662.81	0.573	637.96	0.585
2005	502.23	0.558	679.2	0.799	663.24	0.579	639.47	0.591
2006	502.98	0.564	679.39	0.799	663.62	0.585	641.23	0.598
2007	503.68	0.569	679.75	0.8	663.98	0.588	642.95	0.605
2008	505.03	0.575	680.08	0.803	664.49	0.591	644.77	0.611
2009	505.65	0.58	680.23	0.807	664.25	0.594	646.57	0.617
2010	506.26	0.585	680.73	0.81	664.32	0.598	648.46	0.622
2011	506.63	0.591	681.08	0.812	663.91	0.601	650.29	0.627
2012	507.13	0.596	682.09	0.816	663.91	0.605	652.15	0.632
2013	507.42	0.602	682.24	0.821	664.34	0.609	653.5	0.637
2014	508.08	0.608	682.79	0.825	664	0.612	654.65	0.642
2015	508.07	0.613	683.16	0.828	664.26	0.616	655.45	0.647
2016	508.41	0.618	683.64	0.832	664.26	0.621	655.7	0.651

Back to Top

2017	508.5	0.624	683.39	0.835	664.42	0.625	655.59	0.655
2018	509.18	0.628	682.99	0.837	664.2	0.628	655.45	0.659
2019	509.55	0.632	682.56	0.84	664.13	0.631	655	0.663

Supplementary Table 16 (continued).

Year	Central Europe		Central Latin America		Central Sub-Saharan Africa		East Asia	
	YLD Rate	SDI	YLD Rate	SDI	YLD Rate	SDI	YLD Rate	SDI
1990	670.78	0.641	655.18	0.485	568.85	0.269	522.42	0.447
1991	671.26	0.647	656.41	0.49	568.84	0.273	525.4	0.456
1992	671.94	0.652	657.51	0.495	569.18	0.276	527.95	0.464
1993	672.95	0.658	658.63	0.501	568.8	0.279	530.29	0.473
1994	673.94	0.665	659.85	0.507	568.7	0.281	532.25	0.483
1995	674.98	0.672	660.96	0.512	568.41	0.283	533.69	0.492
1996	675.98	0.678	661.93	0.517	568.3	0.286	533.33	0.502
1997	676.72	0.683	662.74	0.523	568.32	0.289	531.17	0.511
1998	677.67	0.689	663.58	0.528	567.88	0.292	528.22	0.52
1999	678.84	0.695	664.25	0.533	567.82	0.295	525.83	0.528
2000	679.94	0.702	664.91	0.538	568.19	0.298	525.31	0.537
2001	681.26	0.709	665.25	0.543	568.16	0.302	528.89	0.545
2002	682.31	0.715	665.53	0.547	568.21	0.308	536.32	0.554
2003	683.66	0.72	665.68	0.551	568.85	0.313	545.49	0.562
2004	685.19	0.726	666.25	0.554	569.28	0.32	554.04	0.571
2005	686.6	0.731	666.58	0.559	569.54	0.328	560.05	0.58
2006	688.2	0.736	667.99	0.564	570.32	0.336	564.09	0.59
2007	689.55	0.74	670.75	0.569	570.68	0.346	568	0.6
2008	691.01	0.745	674.1	0.575	571.81	0.357	571.84	0.609
2009	692.38	0.75	676.84	0.579	572.51	0.367	574.99	0.618
2010	693.63	0.756	678.38	0.584	573.15	0.378	577.33	0.628
2011	694.92	0.76	678.96	0.589	573.83	0.389	578.98	0.637
2012	695.9	0.764	679.63	0.594	574.19	0.4	580.36	0.644
2013	697.14	0.768	680.18	0.599	574.67	0.412	581.54	0.652
2014	697.98	0.771	680.85	0.604	575.1	0.423	582.53	0.66

Back to Top

2015	698.83	0.775	681.55	0.609	575.07	0.434	583.33	0.662
2016	699.74	0.778	681.76	0.614	575.26	0.445	583.96	0.665
2017	700.29	0.781	682.3	0.618	575.5	0.454	584.52	0.675
2018	700.12	0.785	682.62	0.623	575.33	0.463	589.55	0.684
2019	699.6	0.788	683	0.626	575.41	0.47	600.37	0.691

Supplementary Table 16 (continued).

Year	Eastern Europe		Eastern Sub-Saharan Africa		High-income Asia Pacific		High-income North America	
	YLD Rate	SDI	YLD Rate	SDI	YLD Rate	SDI	YLD Rate	SDI
1990	736.06	0.68	408.01	0.235	558.61	0.767	864.31	0.771
1991	736.25	0.687	406.96	0.239	559.88	0.773	862.77	0.773
1992	735.73	0.697	406.14	0.242	560.54	0.779	860.04	0.777
1993	734.65	0.702	405.65	0.245	561.56	0.785	856.58	0.78
1994	733.56	0.702	405.27	0.249	562.52	0.79	853.23	0.784
1995	732.85	0.705	405.21	0.252	564.01	0.796	849.81	0.787
1996	733.37	0.707	405.24	0.257	565.02	0.801	844.05	0.79
1997	735.89	0.708	405.29	0.261	564.68	0.805	835.1	0.792
1998	740.62	0.709	405.38	0.265	564.16	0.809	825.34	0.794
1999	745.74	0.711	405.56	0.27	563.82	0.813	816.99	0.797
2000	749.71	0.711	405.96	0.275	563.96	0.816	812.2	0.8
2001	752.81	0.713	406.3	0.28	563.99	0.819	809.84	0.805
2002	755.9	0.716	406.71	0.285	563.45	0.823	807.95	0.809
2003	759.47	0.72	407.09	0.29	562.85	0.826	806.39	0.811
2004	762.99	0.727	407.46	0.295	562.57	0.83	805.53	0.814
2005	765.94	0.734	407.92	0.301	562.93	0.833	805.54	0.815
2006	769.04	0.74	408.66	0.307	564.34	0.836	806.62	0.814
2007	772.14	0.745	409.62	0.314	566.56	0.839	808.09	0.817
2008	774.67	0.751	410.87	0.321	568.33	0.842	809.93	0.822
2009	777.2	0.757	412.14	0.328	570.03	0.844	811.88	0.828
2010	779.24	0.762	413.2	0.336	570.64	0.847	813.54	0.834
2011	780.92	0.765	413.84	0.343	571.12	0.85	817.46	0.837
2012	782.02	0.768	414.63	0.351	572.08	0.853	824.25	0.841

Back to Top

2013	782.82	0.772	415.33	0.359	572.98	0.856	831.65	0.844
2014	782.94	0.777	416.03	0.367	574.03	0.859	837.67	0.847
2015	782.85	0.781	416.74	0.375	574.61	0.862	840.01	0.85
2016	782.46	0.785	417.24	0.383	574.77	0.865	838.84	0.854
2017	781.61	0.788	417.8	0.391	574.53	0.868	837.66	0.857
2018	776.12	0.791	418.51	0.399	574.3	0.871	837.11	0.859
2019	765.18	0.793	419.07	0.405	574.1	0.873	836.17	0.86

Supplementary Table 16 (continued).

Year	North Africa and Middle East		Oceania		South Asia		Southeast Asia	
	YLD Rate	SDI	YLD Rate	SDI	YLD Rate	SDI	YLD Rate	SDI
1990	777.72	0.414	635.73	0.383	674.89	0.313	771.15	0.455
1991	777.92	0.423	636.21	0.385	675.08	0.319	772.25	0.463
1992	778.1	0.432	636.67	0.388	675.01	0.325	773.59	0.471
1993	778.4	0.441	637.16	0.391	674.92	0.331	774.83	0.479
1994	778.87	0.45	637.6	0.394	675.02	0.337	776.01	0.488
1995	779.76	0.46	638.2	0.397	675.1	0.344	777.23	0.496
1996	779.64	0.469	639.04	0.4	674.31	0.351	777.22	0.504
1997	778.55	0.478	639.65	0.403	672.31	0.357	775.3	0.512
1998	777.16	0.487	640.65	0.405	669.79	0.364	773.09	0.517
1999	776.32	0.497	641.6	0.408	667.95	0.371	771.45	0.523
2000	776.65	0.506	641.89	0.41	667.74	0.378	771.45	0.528
2001	778.54	0.515	642.47	0.412	668.36	0.385	772.55	0.533
2002	781.24	0.524	642.55	0.413	669	0.391	773.84	0.538
2003	784.68	0.532	642.33	0.414	669.61	0.398	775.12	0.543
2004	787.72	0.541	642.72	0.416	670.42	0.405	776.29	0.548
2005	790.13	0.55	643.55	0.417	671.16	0.413	777.42	0.554
2006	791.64	0.56	643.63	0.419	671.98	0.421	778.72	0.559
2007	793.12	0.569	643.87	0.421	673.08	0.43	780.31	0.566
2008	794.44	0.578	644.61	0.422	674.06	0.438	781.62	0.572
2009	795.66	0.587	644.36	0.424	674.99	0.447	783.09	0.578
2010	796.64	0.595	645.14	0.426	675.89	0.456	783.91	0.585

Back to Top

2011	797.55	0.603	646.1	0.428	675.69	0.465	784.76	0.592
2012	798.51	0.611	647.72	0.431	674.4	0.475	785.5	0.599
2013	799.29	0.619	649.05	0.433	672.66	0.485	786.29	0.606
2014	800.13	0.626	649.78	0.436	671.39	0.495	786.69	0.612
2015	800.41	0.633	650.78	0.44	671.36	0.505	787.29	0.619
2016	799.01	0.64	651.02	0.443	672.21	0.515	787.8	0.626
2017	797.5	0.647	651.52	0.446	672.98	0.525	787.9	0.632
2018	798.9	0.654	651.91	0.449	679.01	0.535	788.28	0.639
2019	802.03	0.66	652.12	0.452	691.28	0.543	788.53	0.644
**Supplementary Table 16 (continued).**								
**Year**	**Southern Latin America**		**Southern Sub-Saharan Africa**		**Tropical Latin America**		**Western Europe**	
	**YLD Rate**	**SDI**	**YLD Rate**	**SDI**	**YLD Rate**	**SDI**	**YLD Rate**	**SDI**
1990	546.43	0.584	602.92	0.513	870.56	0.487	911.62	0.75
1991	546.51	0.589	603.4	0.517	867.39	0.492	915.06	0.756
1992	546.94	0.597	603.88	0.522	864.93	0.497	918.36	0.762
1993	547.19	0.602	604.44	0.526	863.4	0.503	921.51	0.767
1994	547.9	0.608	604.41	0.53	862.55	0.508	923.97	0.772
1995	548.29	0.614	604.72	0.535	862.32	0.513	925.95	0.775
1996	548.59	0.62	604.48	0.539	866.08	0.519	929.41	0.779
1997	548.9	0.625	604.67	0.544	874.48	0.524	935.83	0.782
1998	548.96	0.63	604.46	0.548	884.84	0.529	942.68	0.784
1999	548.85	0.634	604.4	0.553	894.44	0.533	948.39	0.787
2000	549.24	0.64	604.5	0.557	900.71	0.538	950.64	0.79
2001	550	0.644	604.7	0.562	904.81	0.543	950.33	0.794
2002	551.37	0.648	605.31	0.567	908.56	0.547	949.58	0.797
2003	553.05	0.651	606.01	0.571	911.93	0.552	948.65	0.8
2004	554.35	0.653	606.83	0.576	913.79	0.556	948.17	0.802
2005	555.71	0.658	607.76	0.581	913.63	0.561	948.15	0.805
2006	558.19	0.663	608.92	0.586	912.05	0.566	949.01	0.807
2007	563.2	0.664	610.39	0.591	909.92	0.572	950.48	0.81
2008	569.56	0.667	611.92	0.596	907.5	0.578	951.91	0.812

Back to Top

2009	574.41	0.671	613.62	0.601	904.92	0.584	952.88	0.815
2010	577.2	0.676	615.1	0.605	902.5	0.59	953.01	0.817
2011	578.16	0.681	616.47	0.61	900.05	0.597	953.6	0.821
2012	579.08	0.686	617.89	0.614	897.18	0.604	955.25	0.824
2013	579.85	0.689	619.15	0.619	894.23	0.61	957.25	0.827
2014	580.89	0.692	620.05	0.623	891.65	0.617	958.03	0.83
2015	581.63	0.701	620.89	0.628	889.3	0.622	957.15	0.832
2016	582.39	0.71	621.33	0.632	891.75	0.627	950.15	0.835
2017	583.24	0.716	621.9	0.636	894.81	0.632	943.23	0.838
2018	583.68	0.719	622.3	0.639	894	0.636	940.79	0.841
2019	584.45	0.721	622.51	0.642	893.38	0.64	939.14	0.843

Supplementary Table 16 (continued).

Year	Western Sub-Saharan Africa	
	YLD Rate	SDI
1990	707.23	0.268
1991	707.35	0.272
1992	707.3	0.276
1993	707.36	0.28
1994	707.5	0.284
1995	707.56	0.288
1996	707.96	0.292
1997	708.74	0.297
1998	709.86	0.301
1999	710.78	0.306
2000	711.31	0.311
2001	711.24	0.316
2002	710.65	0.322
2003	709.78	0.328
2004	709.14	0.335
2005	709.09	0.343
2006	709.54	0.351

Back to Top

2007	710.17	0.359
2008	710.85	0.367
2009	711.4	0.375
2010	711.76	0.383
2011	711.99	0.391
2012	712.12	0.399
2013	712.07	0.407
2014	712.03	0.415
2015	711.77	0.422
2016	711.66	0.43
2017	711.66	0.437
2018	710.62	0.443
2019	708.41	0.448

Back to Top

Supplementary Table 17. The associations between TTH burden in adolescents and young adults and socio-demographic factor by regions from 1990 to 2019								
Year	Andean Latin America		Australasia		Caribbean		Central Asia	
	YLD Rate	SDI	YLD Rate	SDI	YLD Rate	SDI	YLD Rate	SDI
1990	53.81	0.489	74.91	0.742	60.1	0.517	77.58	0.551
1991	53.89	0.492	75.17	0.746	60.3	0.522	77.63	0.555
1992	54.03	0.496	75.47	0.749	60.47	0.525	77.61	0.557
1993	54.14	0.5	75.86	0.753	60.65	0.528	77.56	0.558
1994	54.2	0.504	76	0.757	60.76	0.531	77.52	0.559
1995	54.3	0.509	76.26	0.761	60.88	0.533	77.44	0.559
1996	54.47	0.514	76.45	0.765	60.97	0.536	77.37	0.56
1997	54.64	0.519	76.7	0.769	61.13	0.539	77.29	0.56
1998	54.82	0.524	76.9	0.773	61.21	0.542	77.36	0.561
1999	55.01	0.529	77.04	0.777	61.29	0.547	77.46	0.563
2000	55.18	0.534	77.24	0.781	61.35	0.552	77.59	0.566
2001	55.39	0.538	77.32	0.785	61.46	0.557	77.75	0.569
2002	55.65	0.543	77.41	0.789	61.58	0.563	78	0.574
2003	55.9	0.548	77.4	0.793	61.71	0.569	78.25	0.579
2004	56.09	0.553	77.35	0.797	61.86	0.573	78.46	0.585
2005	56.25	0.558	77.47	0.799	62	0.579	78.79	0.591
2006	56.42	0.564	77.49	0.799	62.13	0.585	79.12	0.598
2007	56.62	0.569	77.59	0.8	62.27	0.588	79.46	0.605
2008	56.7	0.575	77.67	0.803	62.36	0.591	79.84	0.611
2009	56.9	0.58	77.64	0.807	62.46	0.594	80.11	0.617
2010	57.02	0.585	77.76	0.81	62.58	0.598	80.48	0.622
2011	57.12	0.591	77.71	0.812	62.65	0.601	80.84	0.627
2012	57.34	0.596	77.8	0.816	62.79	0.605	81.14	0.632
2013	57.51	0.602	77.9	0.821	62.83	0.609	81.42	0.637
2014	57.65	0.608	77.93	0.825	62.93	0.612	81.63	0.642
2015	57.79	0.613	77.89	0.828	62.97	0.616	81.8	0.647
2016	57.89	0.618	77.85	0.832	63.11	0.621	81.89	0.651

Back to Top

2017	57.94	0.624	77.73	0.835	63.2	0.625	81.97	0.655
2018	58.11	0.628	77.72	0.837	63.25	0.628	81.96	0.659
2019	58.21	0.632	77.76	0.84	63.29	0.631	81.97	0.663
**Supplementary Table 17 (continued).**								
**Year**	**Central Europe**		**Central Latin America**		**Central Sub-Saharan Africa**		**East Asia**	
	**YLD Rate**	**SDI**	**YLD Rate**	**SDI**	**YLD Rate**	**SDI**	**YLD Rate**	**SDI**
1990	90.34	0.641	60.2	0.485	59.54	0.269	49.88	0.447
1991	90.43	0.647	60.37	0.49	59.54	0.273	50.24	0.456
1992	90.53	0.652	60.56	0.495	59.57	0.276	50.53	0.464
1993	90.7	0.658	60.8	0.501	59.58	0.279	50.82	0.473
1994	90.85	0.665	61	0.507	59.55	0.281	51.08	0.483
1995	91.05	0.672	61.21	0.512	59.54	0.283	51.26	0.492
1996	91.29	0.678	61.42	0.517	59.49	0.286	51.23	0.502
1997	91.49	0.683	61.61	0.523	59.47	0.289	50.88	0.511
1998	91.78	0.689	61.8	0.528	59.47	0.292	50.46	0.52
1999	92.12	0.695	61.96	0.533	59.5	0.295	50.15	0.528
2000	92.48	0.702	62.14	0.538	59.55	0.298	50.22	0.537
2001	92.81	0.709	62.25	0.543	59.54	0.302	50.66	0.545
2002	93.12	0.715	62.38	0.547	59.58	0.308	51.38	0.554
2003	93.37	0.72	62.48	0.551	59.62	0.313	52.22	0.562
2004	93.75	0.726	62.64	0.554	59.66	0.32	52.95	0.571
2005	94.06	0.731	62.77	0.559	59.73	0.328	53.47	0.58
2006	94.31	0.736	62.93	0.564	59.82	0.336	53.81	0.59
2007	94.56	0.74	63.09	0.569	59.94	0.346	54.08	0.6
2008	94.83	0.745	63.24	0.575	60.07	0.357	54.35	0.609
2009	95.05	0.75	63.43	0.579	60.14	0.367	54.58	0.618
2010	95.27	0.756	63.6	0.584	60.22	0.378	54.79	0.628
2011	95.45	0.76	63.78	0.589	60.27	0.389	54.98	0.637
2012	95.62	0.764	63.98	0.594	60.36	0.4	55.16	0.644
2013	95.83	0.768	64.14	0.599	60.43	0.412	55.38	0.652
2014	96.03	0.771	64.33	0.604	60.5	0.423	55.55	0.66

Back to Top

2015	96.24	0.775	64.5	0.609	60.51	0.434	55.73	0.662
2016	96.37	0.778	64.64	0.614	60.52	0.445	55.89	0.665
2017	96.53	0.781	64.82	0.618	60.53	0.454	56.04	0.675
2018	96.61	0.785	64.96	0.623	60.54	0.463	56.14	0.684
2019	96.74	0.788	65.11	0.626	60.53	0.47	56.19	0.691

Supplementary Table 17 (continued).

Year	Eastern Europe		Eastern Sub-Saharan Africa		High-income Asia Pacific		High-income North America	
	YLD Rate	SDI	YLD Rate	SDI	YLD Rate	SDI	YLD Rate	SDI
1990	119.44	0.68	53.26	0.235	79.34	0.767	89.71	0.771
1991	119.43	0.687	53.16	0.239	79.53	0.773	90	0.773
1992	119.22	0.697	53.11	0.242	79.68	0.779	90.25	0.777
1993	118.75	0.702	53.07	0.245	79.9	0.785	90.47	0.78
1994	118.22	0.702	53.03	0.249	80.16	0.79	90.67	0.784
1995	117.83	0.705	53.03	0.252	80.46	0.796	90.88	0.787
1996	117.98	0.707	53.04	0.257	80.67	0.801	91.07	0.79
1997	118.78	0.708	53.07	0.261	80.93	0.805	91.17	0.792
1998	120.28	0.709	53.1	0.265	81.23	0.809	91.22	0.794
1999	121.87	0.711	53.14	0.27	81.55	0.813	91.23	0.797
2000	123.16	0.711	53.16	0.275	81.9	0.816	91.21	0.8
2001	124.08	0.713	53.09	0.28	82.21	0.819	91.05	0.805
2002	124.79	0.716	52.84	0.285	82.27	0.823	90.64	0.809
2003	125.7	0.72	52.53	0.29	82.38	0.826	90.19	0.811
2004	126.52	0.727	52.3	0.295	82.47	0.83	89.85	0.814
2005	127.19	0.734	52.23	0.301	82.56	0.833	89.69	0.815
2006	127.9	0.74	52.26	0.307	82.83	0.836	89.71	0.814
2007	128.59	0.745	52.34	0.314	83.05	0.839	89.7	0.817
2008	129.2	0.751	52.4	0.321	83.36	0.842	89.71	0.822
2009	129.81	0.757	52.48	0.328	83.63	0.844	89.68	0.828
2010	130.28	0.762	52.54	0.336	83.87	0.847	89.65	0.834
2011	130.69	0.765	52.62	0.343	84.1	0.85	89.61	0.837
2012	130.92	0.768	52.7	0.351	84.35	0.853	89.55	0.841

Back to Top

2013	130.97	0.772	52.79	0.359	84.52	0.856	89.46	0.844
2014	130.92	0.777	52.88	0.367	84.76	0.859	89.41	0.847
2015	130.83	0.781	52.95	0.375	85.01	0.862	89.34	0.85
2016	130.64	0.785	53.07	0.383	85.16	0.865	89.26	0.854
2017	130.41	0.788	53.17	0.391	85.25	0.868	89.12	0.857
2018	128.93	0.791	53.31	0.399	85.4	0.871	89.02	0.859
2019	126.01	0.793	53.4	0.405	85.5	0.873	88.95	0.86

Supplementary Table 17 (continued).

Year	North Africa and Middle East		Oceania		South Asia		Southeast Asia	
	YLD Rate	SDI	YLD Rate	SDI	YLD Rate	SDI	YLD Rate	SDI
1990	76.01	0.414	53.04	0.383	58.39	0.313	58.99	0.455
1991	76.07	0.423	53.1	0.385	58.43	0.319	59.12	0.463
1992	76.16	0.432	53.17	0.388	58.45	0.325	59.3	0.471
1993	76.23	0.441	53.26	0.391	58.44	0.331	59.45	0.479
1994	76.38	0.45	53.28	0.394	58.42	0.337	59.63	0.488
1995	76.54	0.46	53.32	0.397	58.4	0.344	59.83	0.496
1996	76.72	0.469	53.46	0.4	58.3	0.351	60.02	0.504
1997	76.85	0.478	53.51	0.403	58.06	0.357	60.21	0.512
1998	77.04	0.487	53.63	0.405	57.81	0.364	60.42	0.517
1999	77.25	0.497	53.67	0.408	57.62	0.371	60.64	0.523
2000	77.49	0.506	53.74	0.41	57.56	0.378	60.81	0.528
2001	77.76	0.515	53.82	0.412	57.61	0.385	61.02	0.533
2002	78.09	0.524	53.86	0.413	57.6	0.391	61.19	0.538
2003	78.43	0.532	53.99	0.414	57.6	0.398	61.4	0.543
2004	78.74	0.541	54.1	0.416	57.62	0.405	61.61	0.548
2005	79.04	0.55	54.06	0.417	57.72	0.413	61.8	0.554
2006	79.37	0.56	54.16	0.419	57.82	0.421	61.94	0.559
2007	79.69	0.569	54.15	0.421	57.92	0.43	62.13	0.566
2008	80	0.578	54.18	0.422	58.03	0.438	62.31	0.572
2009	80.32	0.587	54.29	0.424	58.15	0.447	62.49	0.578
2010	80.62	0.595	54.38	0.426	58.27	0.456	62.69	0.585

Back to Top

2011	80.87	0.603	54.4	0.428	58.38	0.465	62.84	0.592
2012	81.15	0.611	54.56	0.431	58.5	0.475	63	0.599
2013	81.4	0.619	54.67	0.433	58.62	0.485	63.14	0.606
2014	81.6	0.626	54.82	0.436	58.73	0.495	63.26	0.612
2015	81.77	0.633	54.93	0.44	58.85	0.505	63.37	0.619
2016	81.98	0.64	54.99	0.443	58.95	0.515	63.5	0.626
2017	82.1	0.647	54.99	0.446	59.07	0.525	63.59	0.632
2018	82.48	0.654	55.07	0.449	59.63	0.535	63.7	0.639
2019	83.02	0.66	55.08	0.452	60.65	0.543	63.78	0.644

Supplementary Table 17 (continued).

Year	Southern Latin America		Southern Sub-Saharan Africa		Tropical Latin America		Western Europe	
	YLD Rate	SDI	YLD Rate	SDI	YLD Rate	SDI	YLD Rate	SDI
1990	71.05	0.584	64.53	0.513	64.53	0.487	91.64	0.75
1991	71.16	0.589	64.63	0.517	64.12	0.492	91.63	0.756
1992	71.28	0.597	64.75	0.522	63.67	0.497	91.74	0.762
1993	71.34	0.602	64.88	0.526	63.33	0.503	91.86	0.767
1994	71.54	0.608	64.98	0.53	63.15	0.508	92.05	0.772
1995	71.57	0.614	65.06	0.535	63.15	0.513	92.32	0.775
1996	71.68	0.62	65.12	0.539	63.18	0.519	92.76	0.779
1997	71.79	0.625	65.2	0.544	63.01	0.524	93.43	0.782
1998	71.84	0.63	65.26	0.548	62.76	0.529	94.11	0.784
1999	71.83	0.634	65.35	0.553	62.55	0.533	94.68	0.787
2000	71.96	0.64	65.43	0.557	62.51	0.538	95.12	0.79
2001	72.03	0.644	65.53	0.562	62.69	0.543	95.39	0.794
2002	72.14	0.648	65.66	0.567	62.85	0.547	95.57	0.797
2003	72.27	0.651	65.78	0.571	63.06	0.552	95.79	0.8
2004	72.48	0.653	65.96	0.576	63.28	0.556	95.94	0.802
2005	72.7	0.658	66.13	0.581	63.42	0.561	96.15	0.805
2006	72.81	0.663	66.33	0.586	63.6	0.566	96.34	0.807
2007	72.99	0.664	66.56	0.591	63.77	0.572	96.57	0.81
2008	73.19	0.667	66.79	0.596	63.99	0.578	96.78	0.812

Back to Top

2009	73.39	0.671	67.04	0.601	64.15	0.584	97.04	0.815
2010	73.54	0.676	67.32	0.605	64.3	0.59	97.17	0.817
2011	73.76	0.681	67.58	0.61	64.44	0.597	97.26	0.821
2012	73.91	0.686	67.84	0.614	64.58	0.604	97.44	0.824
2013	74.07	0.689	68.08	0.619	64.71	0.61	97.59	0.827
2014	74.3	0.692	68.28	0.623	64.85	0.617	97.65	0.83
2015	74.53	0.701	68.51	0.628	64.96	0.622	97.66	0.832
2016	74.71	0.71	68.61	0.632	66.57	0.627	97.62	0.835
2017	74.89	0.716	68.77	0.636	68.22	0.632	97.57	0.838
2018	75.03	0.719	68.91	0.639	68.48	0.636	97.14	0.841
2019	75.25	0.721	69.07	0.642	68.83	0.64	96.47	0.843

Supplementary Table 17 (continued).

Year	Western Sub-Saharan Africa	
	**YLD Rate**	**SDI**
1990	63.32	0.268
1991	63.31	0.272
1992	63.3	0.276
1993	63.3	0.28
1994	63.3	0.284
1995	63.29	0.288
1996	63.26	0.292
1997	63.18	0.297
1998	63.07	0.301
1999	63	0.306
2000	63.05	0.311
2001	63.1	0.316
2002	63.17	0.322
2003	63.27	0.328
2004	63.35	0.335
2005	63.4	0.343
2006	63.45	0.351

Back to Top

2007	63.51	0.359
2008	63.59	0.367
2009	63.64	0.375
2010	63.68	0.383
2011	63.7	0.391
2012	63.71	0.399
2013	63.67	0.407
2014	63.66	0.415
2015	63.61	0.422
2016	63.56	0.43
2017	63.56	0.437
2018	63.69	0.443
2019	64	0.448

Back to Top

Supplementary Table 18. The associations between burden of overall headaches, migraine, and TTH in adolescents and young adults and socio-demographic factor by country and territory in 2019				
Location	SDI	YLD Rate per 100,000 Population		
		Headache Disorders	Migraine	TTH
Afghanistan	0.343	831.0602	758.2009	72.85929
Albania	0.681	766.2946	675.0805	91.2141
Algeria	0.652	893.4067	810.3325	83.07415
American Samoa	0.712	705.8142	650.2994	55.5148
Andorra	0.894	1003.077	905.8187	97.25801
Angola	0.47	639.9905	579.3595	60.63101
Antigua and Barbuda	0.743	740.9132	675.4616	65.45153
Argentina	0.708	651.9995	577.1956	74.80392
Armenia	0.689	762.1398	676.7028	85.43703
Australia	0.839	755.9928	678.82	77.17278
Austria	0.849	977.1428	873.5953	103.5475
Azerbaijan	0.683	751.9118	667.4747	84.43713
Bahamas	0.796	735.6455	671.058	64.58742
Bahrain	0.751	846.8581	760.7452	86.11291
Bangladesh	0.483	752.946	693.8765	59.06953
Barbados	0.742	741.9548	675.6585	66.29624
Belarus	0.745	817.8006	703.2571	114.5435
Belgium	0.851	1218.923	1126.245	92.67781
Belize	0.603	720.599	659.8777	60.72124
Benin	0.352	761.4527	699.4402	62.0125
Bermuda	0.813	745.9112	677.4659	68.44531
Bhutan	0.455	745.2731	686.5089	58.76419
Bolivia (Plurinational State of)	0.566	574.1233	517.4944	56.6288
Bosnia and Herzegovina	0.718	780.0048	685.8012	94.20357
Botswana	0.634	670.2821	604.3962	65.88584
Brazil	0.64	964.3145	895.2498	69.06475
Brunei Darussalam	0.823	606.9178	530.7317	76.1861
Bulgaria	0.764	785.715	690.1587	95.5563
Burkina Faso	0.257	763.1118	701.0783	62.03348
Burundi	0.284	476.1015	421.171	54.9305
Cabo Verde	0.525	788.4399	720.5624	67.87757
Cambodia	0.469	821.0694	761.4955	59.57391
Cameroon	0.49	760.9362	698.4769	62.4593
Canada	0.873	922.7151	835.472	87.24311
Central African Republic	0.274	640.3912	579.467	60.92417
Chad	0.238	746.3207	686.6316	59.68908
Chile	0.759	678.0121	601.7108	76.30134
China	0.686	656.4058	600.1263	56.27954
Colombia	0.633	751.8322	688.0623	63.7699

Back to Top

Comoros	0.455	501.4269	442.3081	59.11882
Congo	0.568	656.8104	592.8062	64.00417
Cook Islands	0.764	738.4602	679.7601	58.70006
Costa Rica	0.68	745.4471	681.1717	64.27538
Cote d'Ivoire	0.408	761.9018	698.3486	63.5532
Croatia	0.794	777.5437	683.3129	94.23077
Cuba	0.668	736.3433	669.1165	67.22678
Cyprus	0.841	1024.126	928.3209	95.8048
Czechia	0.828	782.8688	687.6462	95.22261
Democratic People's Republic of Korea	0.558	627.5044	575.1847	52.31965
Democratic Republic of the Congo	0.382	632.6463	572.4622	60.18413
Denmark	0.89	882.5435	791.0771	91.46634
Djibouti	0.459	504.9859	445.0724	59.9135
Dominica	0.729	719.5688	656.3356	63.23316
Dominican Republic	0.592	723.2881	661.0097	62.27834
Ecuador	0.64	639.8207	584.6042	55.21644
Egypt	0.658	913.4974	831.6512	81.84619
El Salvador	0.573	746.0162	683.7176	62.29863
Equatorial Guinea	0.685	623.0752	563.6445	59.43075
Eritrea	0.396	484.3021	428.0792	56.22293
Estonia	0.835	805.3647	692.1577	113.207
Eswatini	0.577	650.1305	587.6918	62.43873
Ethiopia	0.343	419.8375	375.5838	44.25372
Fiji	0.664	722.4455	665.1169	57.32855
Finland	0.856	984.0625	892.1057	91.95682
France	0.834	988.8393	889.9371	98.90212
Gabon	0.656	663.8109	599.3476	64.46323
Gambia	0.399	760.672	698.7067	61.96525
Georgia	0.702	755.1646	669.7225	85.44209
Germany	0.898	1084.22	989.26	94.96033
Ghana	0.557	780.7621	715.3682	65.39387
Greece	0.794	1044.787	948.6773	96.10977
Greenland	0.761	889.526	805.2386	84.28741
Grenada	0.669	720.7011	657.279	63.42211
Guam	0.813	723.3812	665.4791	57.90217
Guatemala	0.526	729.7641	669.8532	59.91088
Guinea	0.325	764.3674	702.5065	61.86089
Guinea-Bissau	0.355	766.8347	704.205	62.62962
Guyana	0.618	718.2346	656.733	61.50162
Haiti	0.432	719.0916	659.0352	60.05636
Honduras	0.496	729.7853	670.0164	59.76893
Hungary	0.791	787.3544	692.0086	95.34576
Iceland	0.869	981.8557	891.2421	90.61363
India	0.566	759.2193	697.3113	61.90796

Back to Top

Indonesia	0.66	857.4009	791.8828	65.51809
Iran (Islamic Republic of)	0.67	953.5525	854.8947	98.65784
Iraq	0.671	863.7349	785.7056	78.02938
Ireland	0.867	990.5077	897.6694	92.83828
Israel	0.803	971.3425	883.4521	87.89044
Italy	0.801	1155.089	1054.765	100.3244
Jamaica	0.684	730.7561	667.5866	63.16955
Japan	0.87	656.8397	570.8376	86.00217
Jordan	0.731	856.6458	778.1884	78.45745
Kazakhstan	0.723	745.8073	662.3551	83.4522
Kenya	0.508	514.3337	453.7556	60.5781
Kiribati	0.527	712.9032	657.62	55.28313
Kuwait	0.851	883.1459	799.8187	83.32723
Kyrgyzstan	0.596	730.5238	649.9037	80.62012
Lao People's Democratic Republic	0.49	815.8497	756.7854	59.06423
Latvia	0.82	812.9737	698.577	114.3968
Lebanon	0.708	898.4135	814.8277	83.58577
Lesotho	0.507	651.3462	588.0604	63.28573
Liberia	0.37	756.6262	693.5975	63.02876
Libya	0.709	884.9955	801.5507	83.44475
Lithuania	0.843	736.1498	629.4016	106.7482
Luxembourg	0.895	989.9282	888.1128	101.8154
Madagascar	0.396	487.48	430.9271	56.55289
Malawi	0.384	475.147	420.7496	54.39735
Malaysia	0.737	762.4689	701.4455	61.02334
Maldives	0.562	814.8185	752.1544	62.66406
Mali	0.263	753.1084	692.4911	60.61727
Malta	0.801	1001.853	907.616	94.23737
Marshall Islands	0.544	713.4131	657.3639	56.04922
Mauritania	0.496	765.6688	702.8449	62.82389
Mauritius	0.705	841.8055	778.718	63.08752
Mexico	0.649	754.9726	687.4539	67.51872
Micronesia (Federated States of)	0.58	706.3693	651.0885	55.28072
Monaco	0.902	1000.274	904.148	96.1261
Mongolia	0.606	748.9954	665.377	83.61839
Montenegro	0.791	771.1102	678.5613	92.54895
Morocco	0.548	884.1724	802.615	81.55747
Mozambique	0.307	475.7366	421.2253	54.51132
Myanmar	0.521	831.2514	770.589	60.66238
Namibia	0.612	660.6127	596.6389	63.97378
Nauru	0.618	709.1233	654.063	55.06026
Nepal	0.422	787.8666	727.0627	60.80383
Netherlands	0.883	946.2376	856.8793	89.35833

Back to Top

New Zealand	0.84	784.6702	703.6299	81.04028
Nicaragua	0.517	729.0864	667.8668	61.21966
Niger	0.162	744.3928	685.3512	59.04152
Nigeria	0.515	784.2109	718.417	65.79393
Niue	0.711	723.327	665.2229	58.10409
North Macedonia	0.744	777.1874	683.4113	93.77613
Northern Mariana Islands	0.771	732.3646	673.0325	59.3321
Norway	0.913	1060.596	966.9909	93.60521
Oman	0.783	829.1035	746.9732	82.13033
Pakistan	0.449	698.591	645.0144	53.57658
Palau	0.738	730.8	670.2821	60.51789
Palestine	0.588	854.944	778.7166	76.22744
Panama	0.686	727.3363	664.7007	62.63552
Papua New Guinea	0.394	705.9368	651.0728	54.86397
Paraguay	0.638	897.4471	835.8091	61.63805
Peru	0.648	528.2495	467.9564	60.29309
Philippines	0.623	836.0465	773.0149	63.0316
Poland	0.802	826.9577	725.9289	101.0288
Portugal	0.743	1010.378	914.4078	95.97036
Puerto Rico	0.814	741.6614	675.6578	66.0036
Qatar	0.83	794.8388	712.3487	82.49018
Republic of Korea	0.878	672.4501	587.4264	85.0237
Republic of Moldova	0.696	810.368	697.3578	113.0102
Romania	0.76	780.3474	685.6098	94.7376
Russian Federation	0.805	911.9108	782.7265	129.1842
Rwanda	0.429	489.3974	432.7128	56.68459
Saint Kitts and Nevis	0.746	732.1612	666.9423	65.21896
Saint Lucia	0.67	732.6163	667.5552	65.06108
Saint Vincent and the Grenadines	0.627	723.2972	659.7473	63.54984
Samoa	0.641	694.4032	640.3958	54.00738
San Marino	0.884	1009.813	915.3887	94.42441
Sao Tome and Principe	0.502	769.5532	705.3087	64.24449
Saudi Arabia	0.805	849.8291	768.3279	81.50118
Senegal	0.389	760.4145	697.8711	62.54341
Serbia	0.767	771.8118	678.9962	92.8156
Seychelles	0.724	832.5616	769.2973	63.26433
Sierra Leone	0.347	764.2143	701.5523	62.66199
Singapore	0.861	599.5796	517.9382	81.64136
Slovakia	0.812	785.5151	690.6113	94.90382
Slovenia	0.84	782.3189	686.8056	95.51332
Solomon Islands	0.407	702.3974	648.0396	54.35777
Somalia	0.081	470.7498	416.6984	54.05143
South Africa	0.678	706.5234	634.9294	71.59396
South Sudan	0.363	471.783	417.1209	54.66216

Back to Top

Spain	0.767	1037.981	936.1877	101.7929
Sri Lanka	0.69	836.4714	774.5113	61.96012
Sudan	0.515	864.3438	788.1221	76.22172
Suriname	0.636	722.7778	659.6465	63.13134
Sweden	0.872	976.548	889.1522	87.39583
Switzerland	0.929	910.8273	819.7611	91.06616
Syrian Arab Republic	0.619	871.4172	792.8849	78.53239
Taiwan (Province of China)	0.868	701.5347	646.4658	55.06889
Tajikistan	0.539	719.1829	640.1596	79.02324
Thailand	0.687	940.8928	875.7807	65.11211
Timor-Leste	0.514	785.1203	729.2231	55.89724
Togo	0.417	772.1985	707.9274	64.27103
Tokelau	0.626	716.2374	659.6009	56.63647
Tonga	0.636	708.2647	653.0815	55.1832
Trinidad and Tobago	0.757	731.8577	666.4943	65.3634
Tunisia	0.672	901.9963	817.3241	84.6722
Turkey	0.748	875.9964	792.2573	83.73908
Turkmenistan	0.67	727.1814	645.7699	81.41147
Tuvalu	0.589	707.7296	652.0084	55.72128
Uganda	0.404	473.6845	419.5111	54.17338
Ukraine	0.736	860.3457	739.258	121.0877
United Arab Emirates	0.88	806.0908	718.6004	87.49043
United Kingdom	0.847	975.0191	879.8089	95.21016
United Republic of Tanzania	0.423	498.7576	442.6225	56.13503
United States of America	0.859	925.3803	836.2459	89.13437
United States Virgin Islands	0.799	740.6461	674.5896	66.0565
Uruguay	0.697	664.7728	589.1174	75.65544
Uzbekistan	0.631	730.5233	649.6797	80.84355
Vanuatu	0.485	702.7101	648.3964	54.31371
Venezuela (Bolivarian Republic of)	0.607	737.6375	673.8078	63.82975
Viet Nam	0.617	849.6318	786.4644	63.16731
Yemen	0.412	851.5709	776.6224	74.94841
Zambia	0.505	529.6808	466.7337	62.94711
Zimbabwe	0.476	651.6015	589.5659	62.03559

Back to Top
